# Supplementary material for: The Combination of Both Heat and Water Stresses May Worsen Botryosphaeria Dieback Symptoms in Grapevine
Source: Plants (Basel). 2023 Feb 8;12(4):753. doi: 10.3390/plants12040753 (PMC9961737; doi:10.3390/plants12040753)
Supplement: Supplementary file 1 [file plants-12-00753-s001.zip › Supp Figure_Fernandez et al_GTD_Stress_Dec_2022.pptx]

## Slide 1
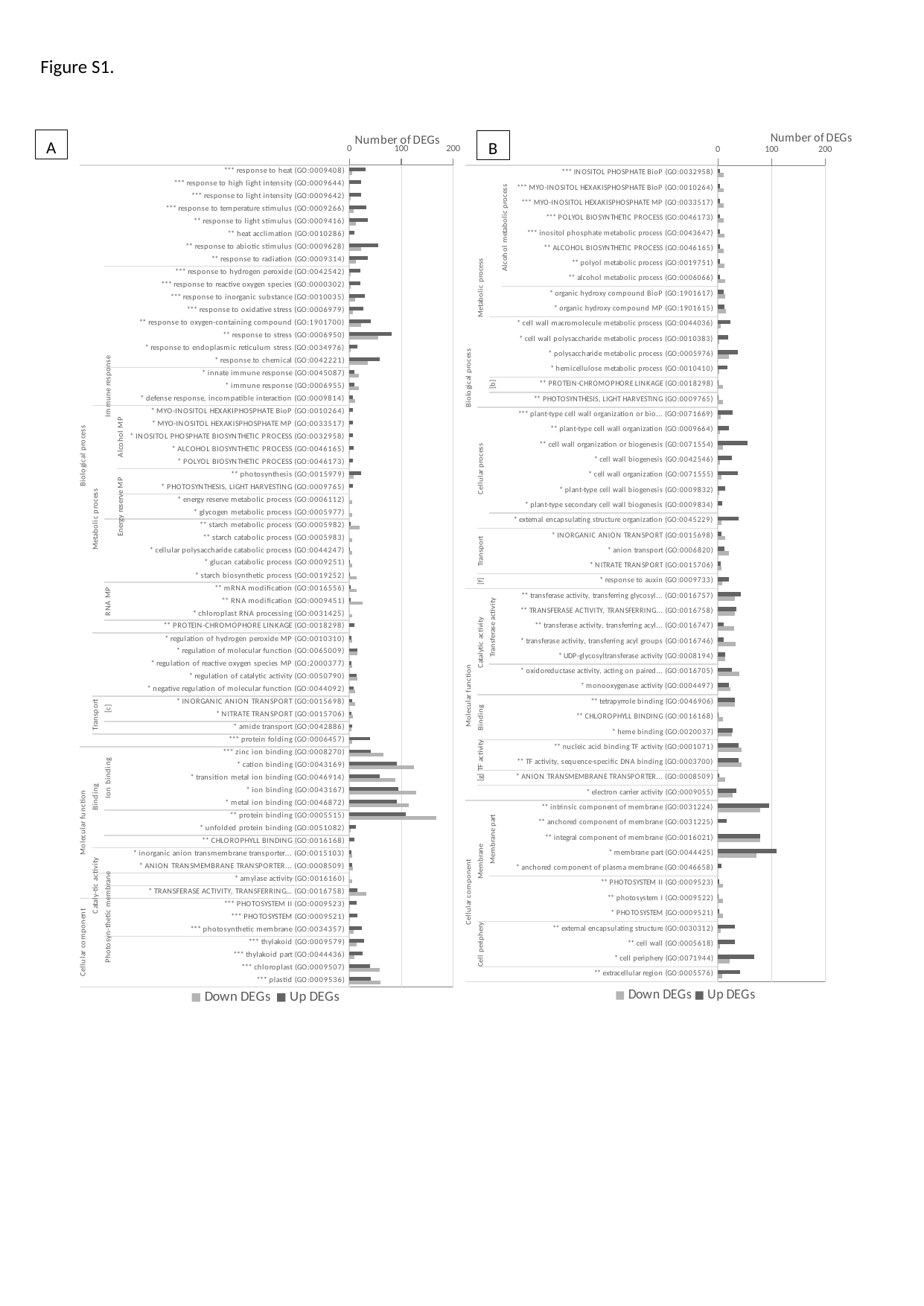

Figure S1.
### Chart
| Category | | |
|---|---|---|
| *** INOSITOL PHOSPHATE BioP (GO:0032958) | 4.0 | 11.0 |
| *** MYO-INOSITOL HEXAKISPHOSPHATE BioP (GO:0010264) | 4.0 | 11.0 |
| *** MYO-INOSITOL HEXAKISPHOSPHATE MP (GO:0033517) | 4.0 | 11.0 |
| *** POLYOL BIOSYNTHETIC PROCESS (GO:0046173) | 4.0 | 11.0 |
| *** inositol phosphate metabolic process (GO:0043647) | 4.0 | 12.0 |
| ** ALCOHOL BIOSYNTHETIC PROCESS (GO:0046165) | 4.0 | 11.0 |
| ** polyol metabolic process (GO:0019751) | 4.0 | 12.0 |
| ** alcohol metabolic process (GO:0006066) | 4.0 | 13.0 |
| * organic hydroxy compound BioP (GO:1901617) | 11.0 | 13.0 |
| * organic hydroxy compound MP (GO:1901615) | 12.0 | 15.0 |
| * cell wall macromolecule metabolic process (GO:0044036) | 24.0 | 5.0 |
| * cell wall polysaccharide metabolic process (GO:0010383) | 19.0 | 3.0 |
| * polysaccharide metabolic process (GO:0005976) | 37.0 | 21.0 |
| * hemicellulose metabolic process (GO:0010410) | 18.0 | 2.0 |
| ** PROTEIN-CHROMOPHORE LINKAGE (GO:0018298) | 1.0 | 9.0 |
| ** PHOTOSYNTHESIS, LIGHT HARVESTING (GO:0009765) | 1.0 | 9.0 |
| *** plant-type cell wall organization or bio... (GO:0071669) | 27.0 | 5.0 |
| ** plant-type cell wall organization (GO:0009664) | 20.0 | 4.0 |
| ** cell wall organization or biogenesis (GO:0071554) | 55.0 | 9.0 |
| * cell wall biogenesis (GO:0042546) | 26.0 | 4.0 |
| * cell wall organization (GO:0071555) | 37.0 | 6.0 |
| * plant-type cell wall biogenesis (GO:0009832) | 14.0 | 2.0 |
| * plant-type secondary cell wall biogenesis (GO:0009834) | 8.0 | 0.0 |
| * external encapsulating structure organization (GO:0045229) | 38.0 | 6.0 |
| * INORGANIC ANION TRANSPORT (GO:0015698) | 7.0 | 13.0 |
| * anion transport (GO:0006820) | 12.0 | 20.0 |
| * NITRATE TRANSPORT (GO:0015706) | 5.0 | 7.0 |
| * response to auxin (GO:0009733) | 20.0 | 8.0 |
| ** transferase activity, transferring glycosyl... (GO:0016757) | 43.0 | 32.0 |
| ** TRANSFERASE ACTIVITY, TRANSFERRING... (GO:0016758) | 35.0 | 31.0 |
| ** transferase activity, transferring acyl... (GO:0016747) | 11.0 | 30.0 |
| * transferase activity, transferring acyl groups (GO:0016746) | 11.0 | 33.0 |
| * UDP-glycosyltransferase activity (GO:0008194) | 13.0 | 13.0 |
| * oxidoreductase activity, acting on paired... (GO:0016705) | 26.0 | 40.0 |
| * monooxygenase activity (GO:0004497) | 20.0 | 23.0 |
| ** tetrapyrrole binding (GO:0046906) | 32.0 | 32.0 |
| ** CHLOROPHYLL BINDING (GO:0016168) | 1.0 | 9.0 |
| * heme binding (GO:0020037) | 28.0 | 26.0 |
| ** nucleic acid binding TF activity (GO:0001071) | 39.0 | 44.0 |
| ** TF activity, sequence-specific DNA binding (GO:0003700) | 39.0 | 44.0 |
| * ANION TRANSMEMBRANE TRANSPORTER... (GO:0008509) | 2.0 | 13.0 |
| * electron carrier activity (GO:0009055) | 34.0 | 27.0 |
| ** intrinsic component of membrane (GO:0031224) | 95.0 | 79.0 |
| ** anchored component of membrane (GO:0031225) | 16.0 | 0.0 |
| ** integral component of membrane (GO:0016021) | 79.0 | 79.0 |
| * membrane part (GO:0044425) | 109.0 | 72.0 |
| * anchored component of plasma membrane (GO:0046658) | 7.0 | 0.0 |
| ** PHOTOSYSTEM II (GO:0009523) | 3.0 | 10.0 |
| ** photosystem I (GO:0009522) | 1.0 | 9.0 |
| * PHOTOSYSTEM (GO:0009521) | 3.0 | 10.0 |
| ** external encapsulating structure (GO:0030312) | 32.0 | 5.0 |
| ** cell wall (GO:0005618) | 32.0 | 4.0 |
| * cell periphery (GO:0071944) | 68.0 | 22.0 |
| ** extracellular region (GO:0005576) | 41.0 | 8.0 |
### Chart
| Category | | |
|---|---|---|
| *** response to heat (GO:0009408) | 31.0 | 5.0 |
| *** response to high light intensity (GO:0009644) | 23.0 | 1.0 |
| *** response to light intensity (GO:0009642) | 23.0 | 3.0 |
| *** response to temperature stimulus (GO:0009266) | 33.0 | 8.0 |
| ** response to light stimulus (GO:0009416) | 36.0 | 12.0 |
| ** heat acclimation (GO:0010286) | 9.0 | 0.0 |
| ** response to abiotic stimulus (GO:0009628) | 55.0 | 23.0 |
| ** response to radiation (GO:0009314) | 36.0 | 12.0 |
| *** response to hydrogen peroxide (GO:0042542) | 21.0 | 2.0 |
| *** response to reactive oxygen species (GO:0000302) | 21.0 | 2.0 |
| *** response to inorganic substance (GO:0010035) | 30.0 | 11.0 |
| *** response to oxidative stress (GO:0006979) | 27.0 | 7.0 |
| ** response to oxygen-containing compound (GO:1901700) | 41.0 | 23.0 |
| ** response to stress (GO:0006950) | 81.0 | 55.0 |
| * response to endoplasmic reticulum stress (GO:0034976) | 15.0 | 2.0 |
| * response to chemical (GO:0042221) | 58.0 | 35.0 |
| * innate immune response (GO:0045087) | 10.0 | 18.0 |
| * immune response (GO:0006955) | 10.0 | 18.0 |
| * defense response, incompatible interaction (GO:0009814) | 7.0 | 11.0 |
| * MYO-INOSITOL HEXAKIPHOSPHATE BioP (GO:0010264) | 7.0 | 1.0 |
| * MYO-INOSITOL HEXAKISPHOSPHATE MP (GO:0033517) | 7.0 | 1.0 |
| * INOSITOL PHOSPHATE BIOSYNTHETIC PROCESS (GO:0032958) | 7.0 | 1.0 |
| * ALCOHOL BIOSYNTHETIC PROCESS (GO:0046165) | 8.0 | 1.0 |
| * POLYOL BIOSYNTHETIC PROCESS (GO:0046173) | 7.0 | 1.0 |
| ** photosynthesis (GO:0015979) | 23.0 | 8.0 |
| * PHOTOSYNTHESIS, LIGHT HARVESTING (GO:0009765) | 7.0 | 0.0 |
| * energy reserve metabolic process (GO:0006112) | 0.0 | 5.0 |
| * glycogen metabolic process (GO:0005977) | 0.0 | 5.0 |
| ** starch metabolic process (GO:0005982) | 2.0 | 20.0 |
| ** starch catabolic process (GO:0005983) | 0.0 | 6.0 |
| * cellular polysaccharide catabolic process (GO:0044247) | 1.0 | 6.0 |
| * glucan catabolic process (GO:0009251) | 1.0 | 6.0 |
| * starch biosynthetic process (GO:0019252) | 1.0 | 14.0 |
| ** mRNA modification (GO:0016556) | 2.0 | 14.0 |
| ** RNA modification (GO:0009451) | 3.0 | 25.0 |
| * chloroplast RNA processing (GO:0031425) | 0.0 | 5.0 |
| ** PROTEIN-CHROMOPHORE LINKAGE (GO:0018298) | 9.0 | 0.0 |
| * regulation of hydrogen peroxide MP (GO:0010310) | 4.0 | 6.0 |
| * regulation of molecular function (GO:0065009) | 16.0 | 16.0 |
| * regulation of reactive oxygen species MP (GO:2000377) | 4.0 | 6.0 |
| * regulation of catalytic activity (GO:0050790) | 14.0 | 16.0 |
| * negative regulation of molecular function (GO:0044092) | 8.0 | 11.0 |
| * INORGANIC ANION TRANSPORT (GO:0015698) | 6.0 | 11.0 |
| * NITRATE TRANSPORT (GO:0015706) | 4.0 | 7.0 |
| * amide transport (GO:0042886) | 5.0 | 4.0 |
| *** protein folding (GO:0006457) | 40.0 | 6.0 |
| *** zinc ion binding (GO:0008270) | 41.0 | 65.0 |
| * cation binding (GO:0043169) | 92.0 | 124.0 |
| * transition metal ion binding (GO:0046914) | 58.0 | 88.0 |
| * ion binding (GO:0043167) | 94.0 | 129.0 |
| * metal ion binding (GO:0046872) | 92.0 | 115.0 |
| ** protein binding (GO:0005515) | 108.0 | 167.0 |
| * unfolded protein binding (GO:0051082) | 12.0 | 2.0 |
| ** CHLOROPHYLL BINDING (GO:0016168) | 9.0 | 0.0 |
| * inorganic anion transmembrane transporter... (GO:0015103) | 4.0 | 6.0 |
| * ANION TRANSMEMBRANE TRANSPORTER... (GO:0008509) | 5.0 | 7.0 |
| * amylase activity (GO:0016160) | 0.0 | 6.0 |
| * TRANSFERASE ACTIVITY, TRANSFERRING… (GO:0016758) | 15.0 | 32.0 |
| *** PHOTOSYSTEM II (GO:0009523) | 14.0 | 1.0 |
| *** PHOTOSYSTEM (GO:0009521) | 16.0 | 1.0 |
| *** photosynthetic membrane (GO:0034357) | 24.0 | 8.0 |
| *** thylakoid (GO:0009579) | 28.0 | 14.0 |
| *** thylakoid part (GO:0044436) | 25.0 | 9.0 |
| *** chloroplast (GO:0009507) | 40.0 | 58.0 |
| *** plastid (GO:0009536) | 41.0 | 60.0 |A
B

## Slide 2
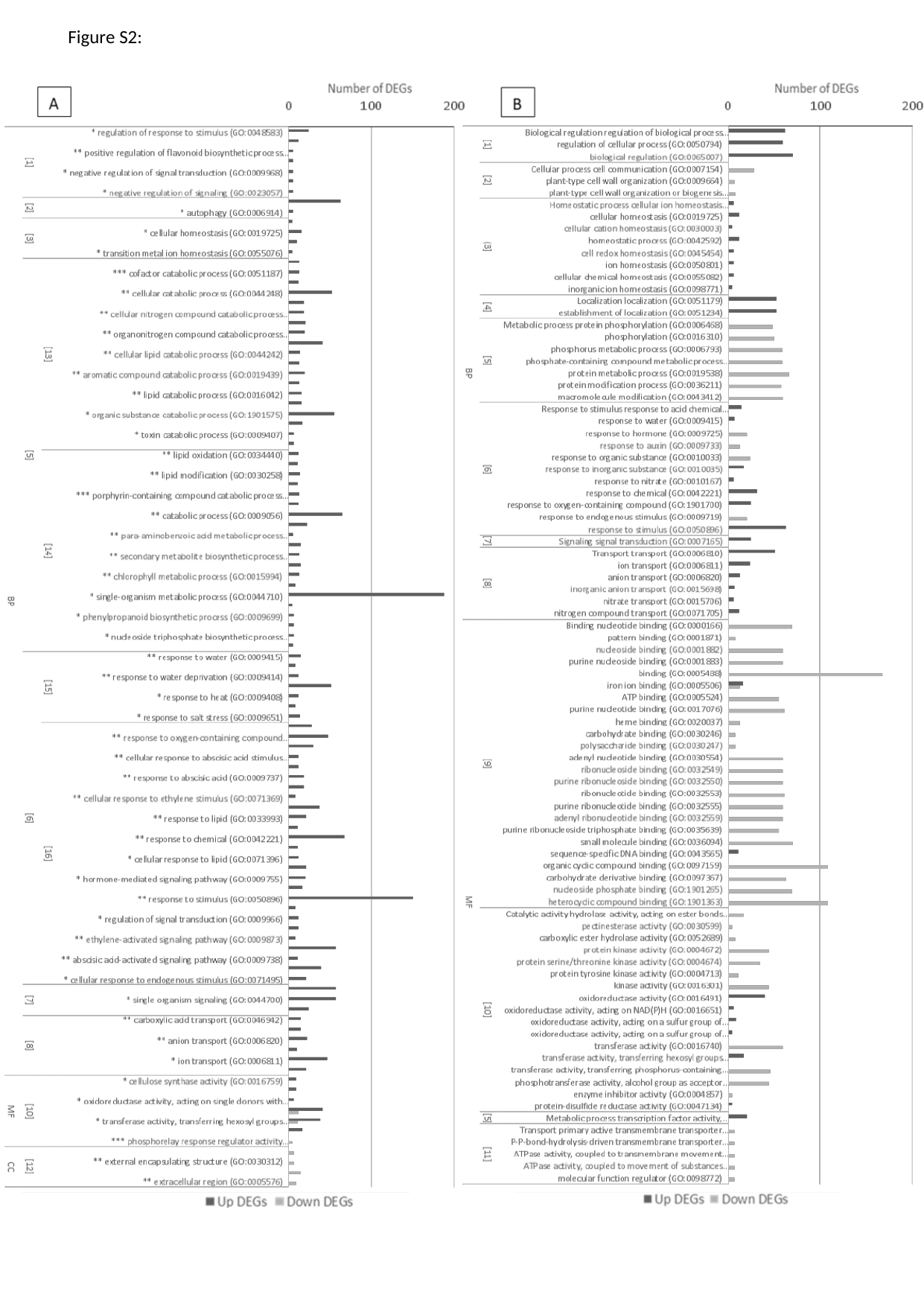

Figure S2:

## Slide 3
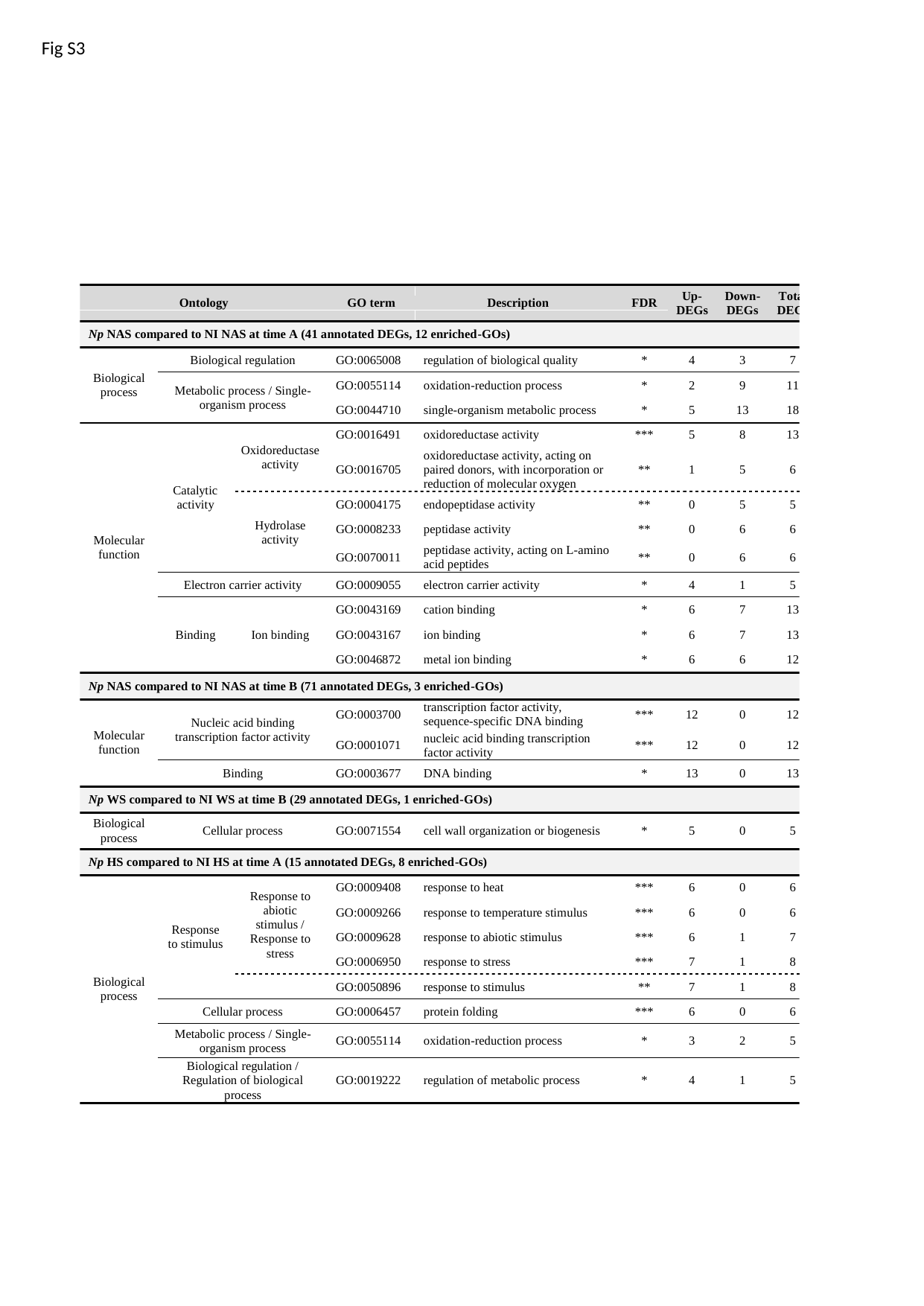

Fig S3

## Slide 4
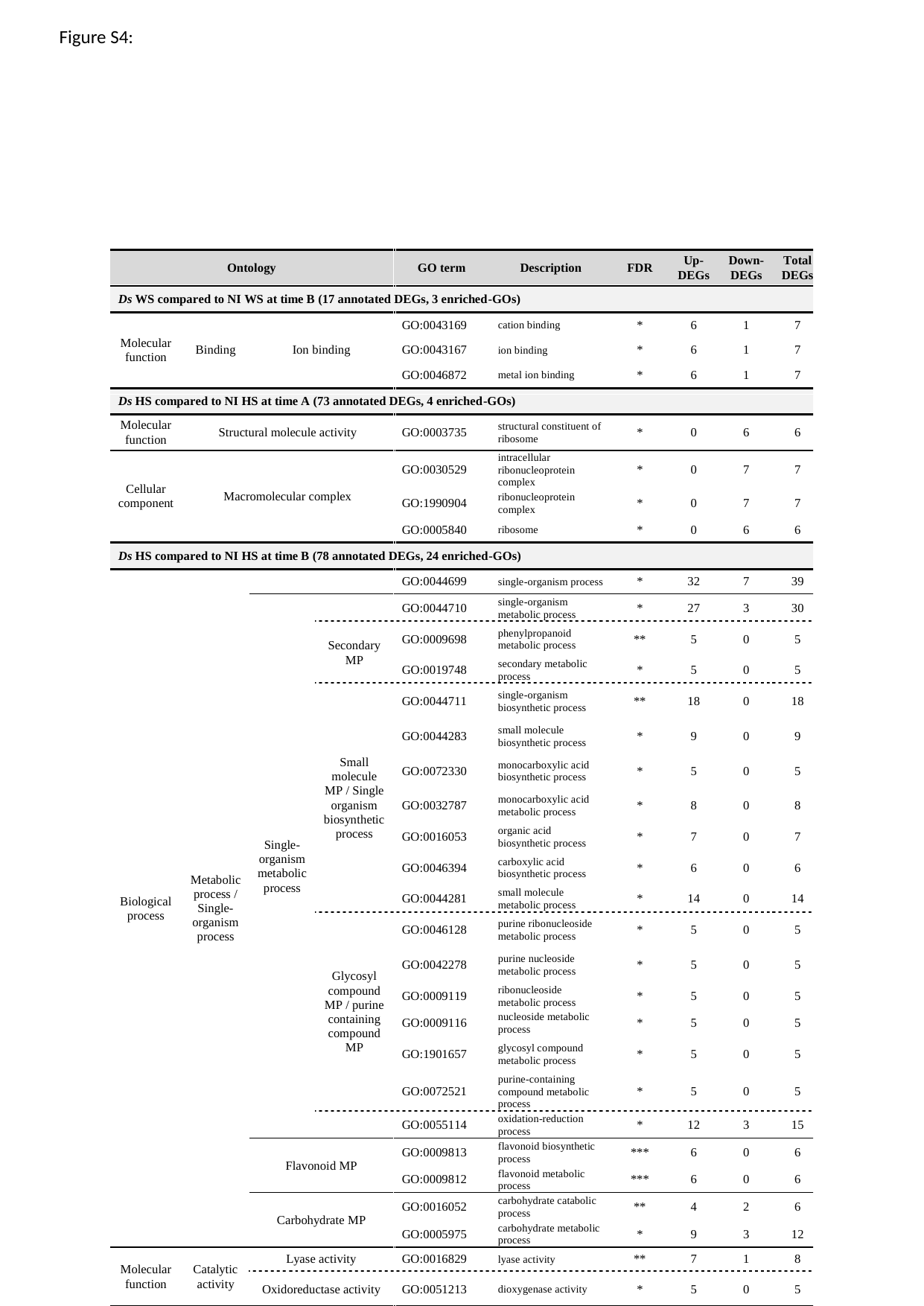

Figure S4:

## Slide 5
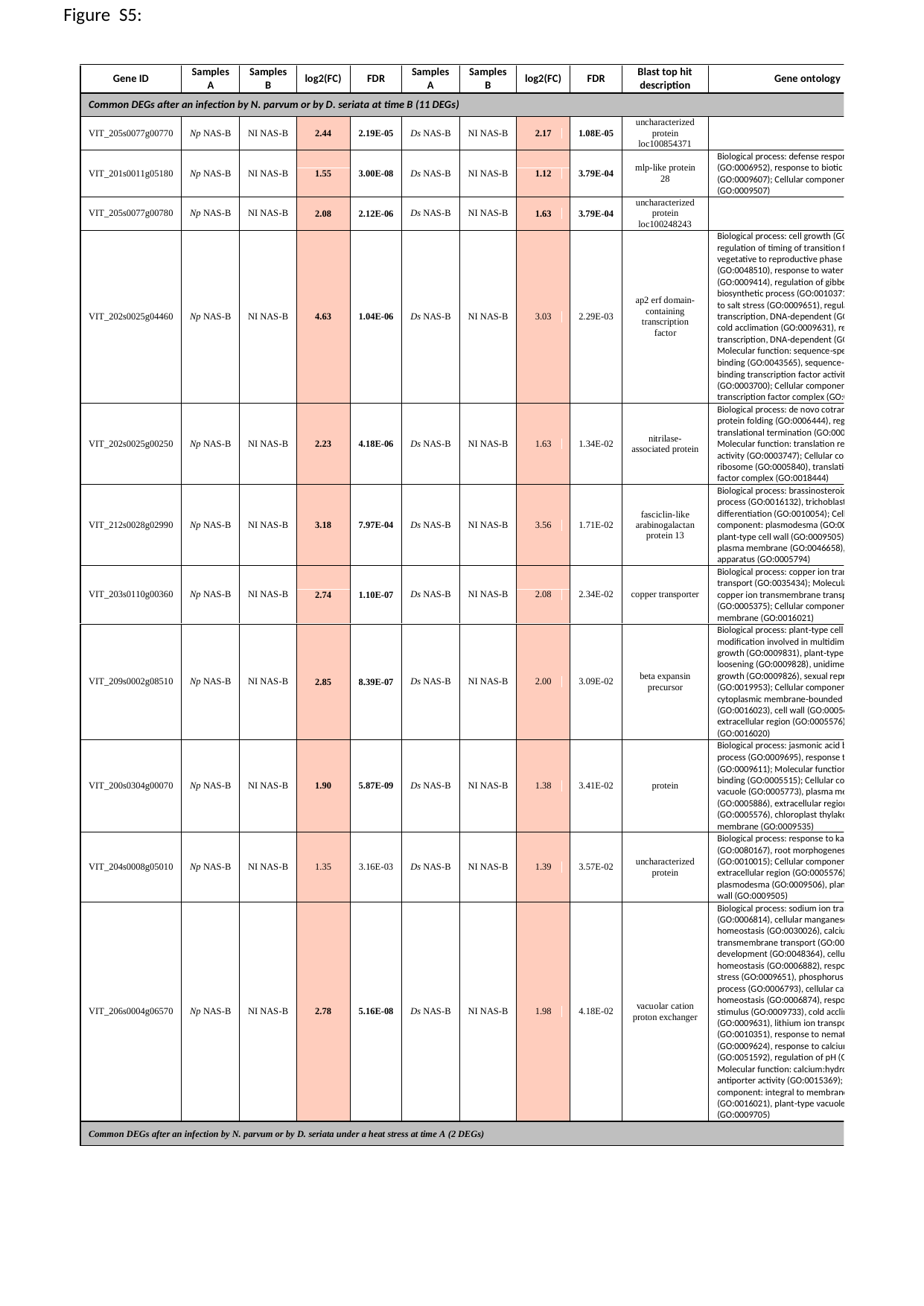

Figure S5:

## Slide 6
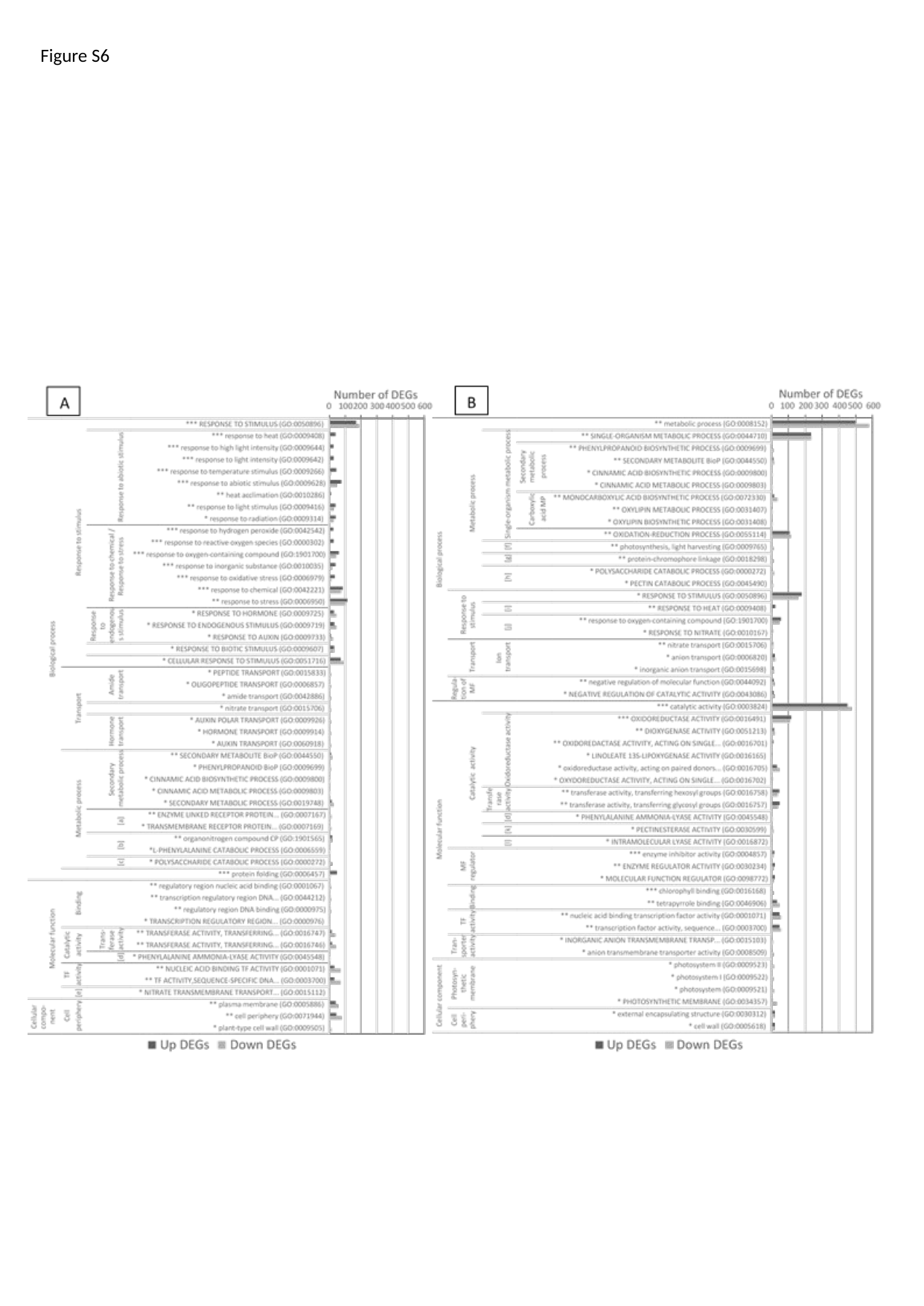

Figure S6

## Slide 7
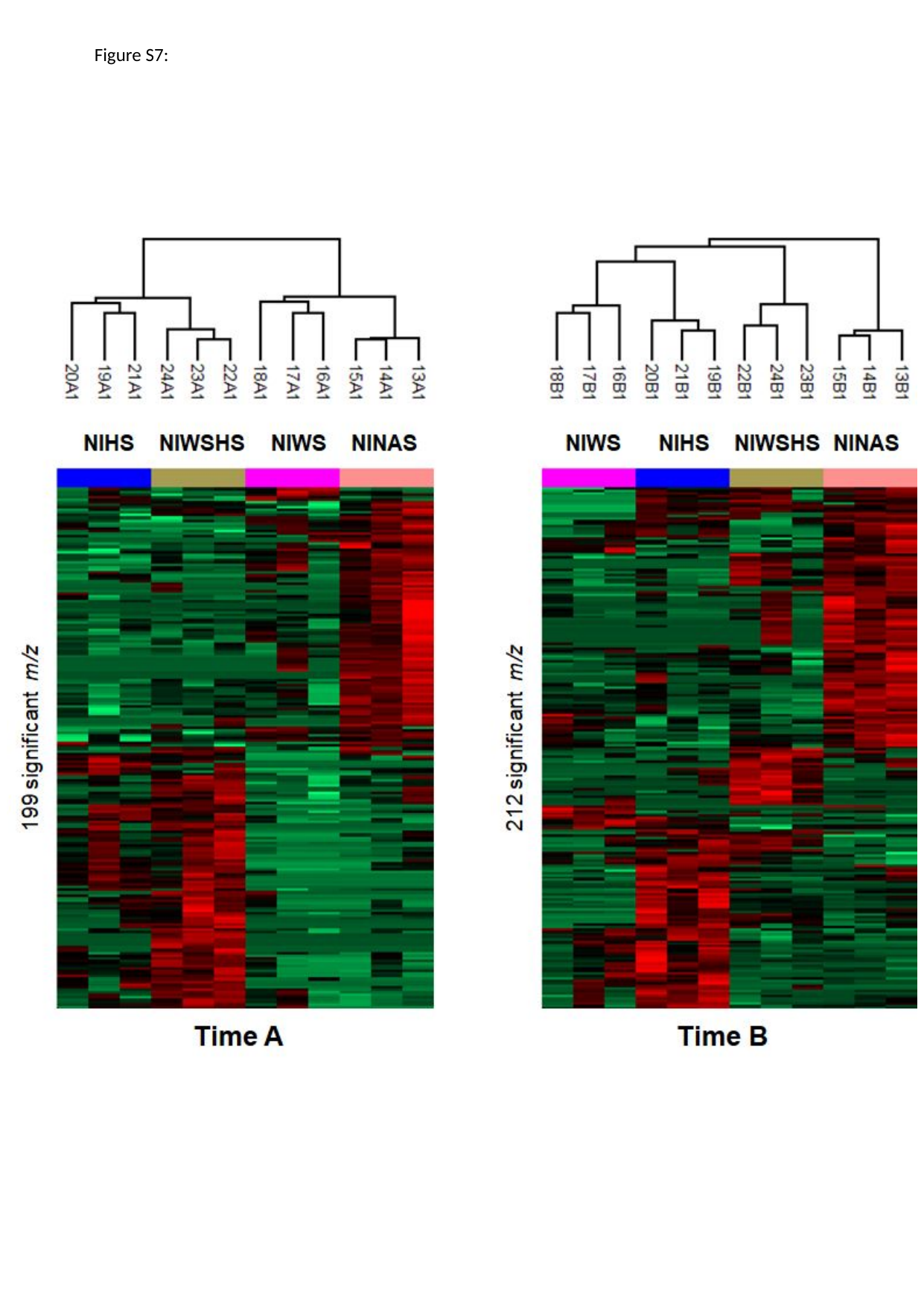

Figure S7:

## Slide 8
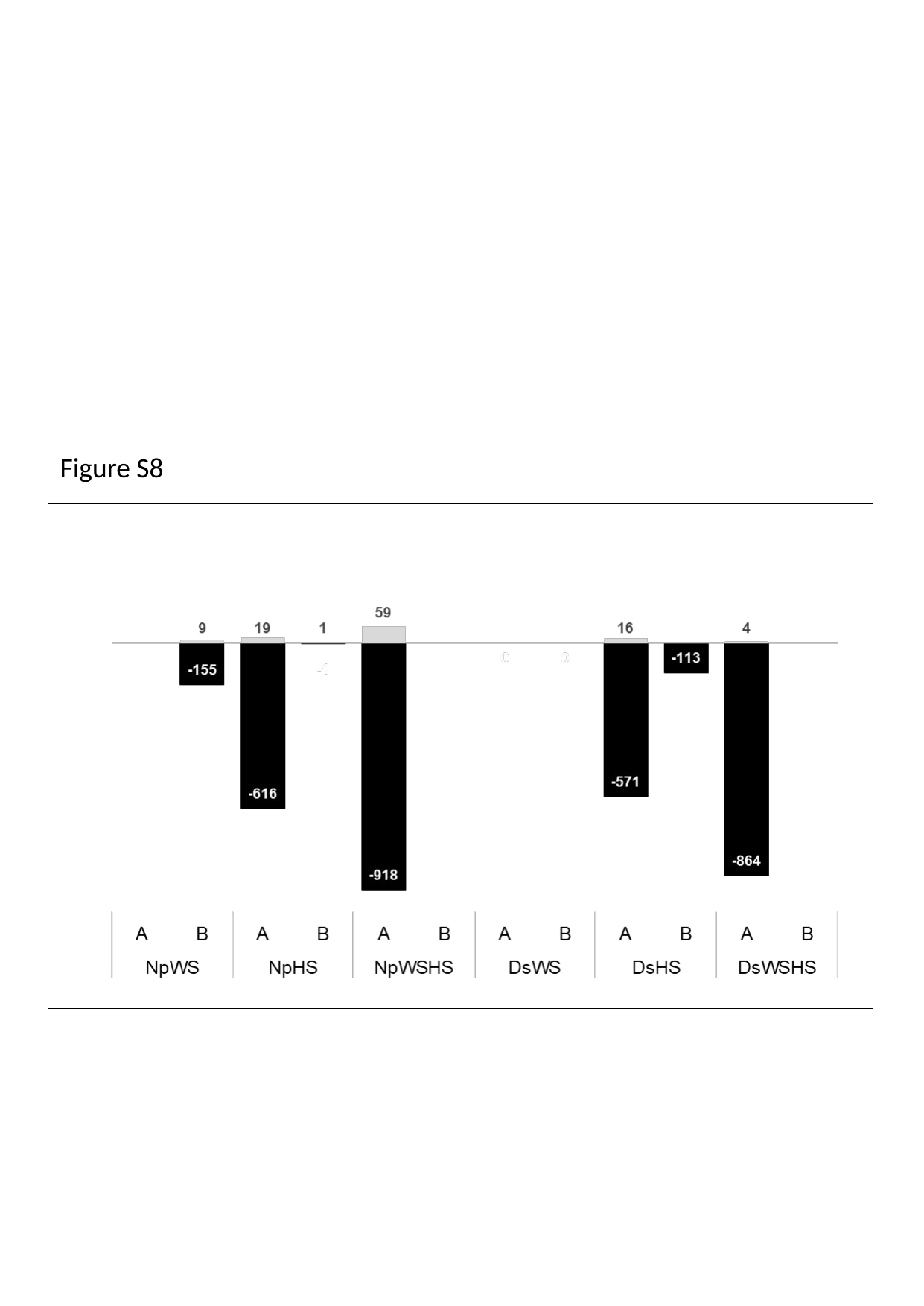

Figure S8
