# Supplementary material for: The Combination of Both Heat and Water Stresses May Worsen Botryosphaeria Dieback Symptoms in Grapevine
Source: Plants (Basel). 2023 Feb 8;12(4):753. doi: 10.3390/plants12040753 (PMC9961737; doi:10.3390/plants12040753)
Supplement: Supplementary file 1 [file plants-12-00753-s001.zip › Supplemental File S4.pdf]

Supplemental File 4 : List of annotated compounds regulated in Time A and B on the non-inoculated samples (NI) under the 3 abiotic stress, water (WS), heat (HS) and combined water and heat (WSHS) stresses. Up- and down-regulated compounds under stresses are indicated with the + and – signs, together with their attributed identification number (ID) and structural categories (Cat.); Phytochemicals (Ph), Lipids (Lp), Carbohydrates (Cb), Peptides (Pp), Amino sugars (AmS) and undetermined or drug (NM).

Table a): List the 14 annotated compounds from the 156 *m/z* regulated in Time A

ID	WS A	HS A	WSHS A	Cat.	Name
3213			+	Ph	Nagilactone C
1750			+	Pp	Glutathione
7699			+	Ph	Corilagin
3099			+	Ph	Gentiopicrin (isotope)
8238			-	Lp	PA(14:1(9Z)/22:4(7Z,10Z,13Z,16Z))
2983		-	-	Ph	2-Caffeoylisocitrate
1347		-	-	NM	Oxazepam
2482		-	-	Ph	3-Epihydroxymugineic acid
8306		-	-	Ph	Hexaacetylpyracanthoside
8254			-	Lp	PA(16:/20:4(5Z,8Z,11Z,14Z))
2215		-	-	Ph	Sinapoyl tartronate
8177			-	Lp	PA(13:/22:2(13Z,16Z))
2804		-	-	NM	Compound VII

Table b: List of the 36 annotated compounds from the 173 *m/z* regulated in Time B

ID	WS B	HS B	WSHS B	Cat.	Name
15	+	+		Ph	Phenylpyruvate
2127		+		Pp	Tetraneurin A
4380		+		Ph	Khellol glucoside
1219		+		Ph	Lactucin
2074		+		Cb	Valproic acid glucuronide
1512	+	+		Ph	Phaseolic acid
4953		+		Ph	Ginkgolide C
1191		+		Lp	3-Hydroxytetradecanedioic acid
66		+		Cb	D-Glucono-1,5-lactone
2413	+	+		Ph	3,5,6,7,2',3',4'-Heptahydroxyflavone
156		+		Ph	3-(3,4-Dihydroxyphenyl) lactate
9		+		Lp	2-Oxoadipate
1392			+	Pp	Ophthalmic acid
2579			+	Cb	Methyl-2-alpha-L-fucopyranosyl-beta-D-galactoside
487			+	AmS	Linamarin
3963	-	+		Ph	Monotropein
187		+	-	Ph	Oxaloglutarate
8451	-	-	-	Ph	Yamogenin 3-O-neohesperidoside
5361	-	-		Cb	Patrinoside
5002	-	-		Lp	PG(6:/6:); 1,2-dihexanoyl-sn-glycero-3-phospho-(1'-sn-glycerol)
199	-	-	-	Ph	(R)-2-Hydroxybutane-1,2,4-tricarboxylate; Homocitrate
3597	-	-		Lp	17beta-Hydroxy-7alpha-mercaptoandrost-4-en-3-one 7-propionate
2486	-	-		Lp	Steroid O-sulfate
2931	-	-		Lp	Estradiol-17beta 3-sulfate
3664	-	-		AmS	4-Deoxy-beta-D-gluc-4-enuronosyl-(1,3)-N-acetyl-D-galactosamine
7463	-	-	-	Ph	Atalanine
3413	-			Lp	Tetracosanoic acid
8600	-	-	-	Lp	1-Hexadecanoyl-2-(9Z-octadecenoyl)-sn-glycero-3-phospho-sn-glycerol
120	-	-	-	Pp	N-Acetyl-L-glutamate
6097	-			Cb	Raffinose

2681			-	Cb	Sucrose (isotope)
8293	-	-		Lp	PA(18:/18:2(9Z,12Z)); 1-octadecanoyl-2-(9Z,12Z-octadecadienoyl)-sn-glycero-3-phosphate
5540	-	-		Ph	Proteacin
1590	-	-		Cb	Salidroside
2614			-	Cb	Sucrose

Table c) List of 21 *m/z* common to A and B time-points

ID	WS A	WS B	HS A	HS B	WSHS A	WSHS B	Cat.	Name
5304					+	+		
6229		-		-	+			
6769					+	+		
3091			-	-	-	-		
2840		-		-	+			
3810			+		+	+		
214			+	+				
1844					+	+		
5098			-	-	-	-		
5544					+	+		
5268					+	+		
3112			-	+	-			
5136	-		-	-	-	-		
3378	-		-	+	-			
191					+	+	Pp	L-Tryptophan
4247		-	+		+			
4783			-	-	-	-		
8217		-			+			
3204		+	-	+	-	+		
1538					+	+		
7891			-	+	-			
