# Supplementary material for: The Combination of Both Heat and Water Stresses May Worsen Botryosphaeria Dieback Symptoms in Grapevine
Source: Plants (Basel). 2023 Feb 8;12(4):753. doi: 10.3390/plants12040753 (PMC9961737; doi:10.3390/plants12040753)
Supplement: Supplementary file 1 [file plants-12-00753-s001.zip › Supplemental Table 1.pdf]

Supplemental Table 1

	Genes	Genes ID	Primers sequences	Genbank no.	Efficacity
Housekeeping genes	*EF1-α* (elongation factor 1-α)	VIT_206s0004g03240	5’-AACCAAAATATCCGGAGTAAAAGA-3’<br>5’-GAACTGGGTGCTTGATAGGC-3’	XM_002284888.3	100 %
	39SRP (39S ribosomal proteinL41-A)	VIT_218s0001g06410	5’-GACTGACTTCAAGCTTAAACC-3’<br>5’-GATATAACAGGGAATACAGCAC-3’	XM_002285709.4	100 %
Photosynthesis	*CAB1* (chlorophyll a-b binding protein)	VIT_207s0005g02220	5’-GGAAATCAAGAACGGGCGTC-3'<br>5’-GCGTTGTTGTTCACTGGGTC-3’	XM_002273070.4	96.1 %
Defense	*CHIT1B* (chitinase class I basic)	VIT_203s0038g03400	5’-CCCAAGCCTTCCTGCCATA-3’<br>5’-TGTGATAACACCAAAACCGGG-3’	NM_001280962.1	101.7 %
	PR5 (thaumatin-like protein)	VIT_202s0025g04270	5’-TTTGATCATCCTTAGGGTAGCTGTAA-3’<br>5’-ACCAATGAGTACTGTTGCAATTCC-3’	XM_002282994.3	99.6 %
Secondary cell wall biogenesis	*FLA11* (fasciclin-like arabinogalactan protein 11)	VIT_208s0040g01970	5’-CGAAACCCCAAAGCCTAAGAA-3’<br>5’-GAAAACACAAAGGGGTTGCA-3’	XM_002280793.2	98.7 %
Detoxification	*APX* (cytosolic ascorbate peroxidase)	VIT_208s0040g03150	5’-GGATTTGCTGATGCTGAGGAAC-3’<br>5’-ACCCAAATCACCGAGAACTTCC-3’	EU280159.1	97.2 %
	GSTF9 (Glutathione S-transferase F9)	VIT_212s0028g00930	5’-GGAGCAGTGGCTAGAGGTTGA-3’<br>5’-CATCTGCAGGAAACCCCATCT-3’	XM_002283173.3	101.6 %
Stress tolerance	*sHSP* (class I small heat shock protein)	VIT_206s0004g05770	5’-TCCTCGTGAAATGTGGGACC-3’<br>5’-TCTTCAGCCCAGGAAGGTCT-3’	XM_002281470.3	90.4 %
	PIP2-2 (plasma membrane 2-2 aquaporin)	VIT_203s0038g02520	5’-TCTCTTATCCGGGCTTTGGC-3’<br>5’-GCTGTGCCTTTGTTGTAGCC-3’	NM_001281028.1	93.4 %
Flavonoid/ isoflavonoid biosynthesis pathways	*CHS* (chalcone synthase)	VIT_214s0068g00920	5’-CGAAGGAGCAATCGACGGA-3’<br>5’-GTCGCTGATGCCTATCGGAG-3’	NM_001280950.1	99.6 %
Transcription factor	*CRF2* (ethylene-responsive transcription factor CRF2)	VIT_202s0025g01055	5’-ACCTATGATACGGCGGAGGA-3’<br>5’-CACCGTCACCGGCTCTATTT-3’	XM_002271490.3	99.5 %
