# Supplementary material for: The Combination of Both Heat and Water Stresses May Worsen Botryosphaeria Dieback Symptoms in Grapevine
Source: Plants (Basel). 2023 Feb 8;12(4):753. doi: 10.3390/plants12040753 (PMC9961737; doi:10.3390/plants12040753)
Supplement: Supplementary file 1 [file plants-12-00753-s001.zip › Supplemental_File_S3.pdf]

LIST OF STRESS MODULATED IDENTIFIED COMPOUNDS

Lists of annotated significant *m/z* between fungus *Neofusicoccum parvum* and *Diplodia seriata* (*Np* or *Ds*) inoculated-grapevines and non-inoculated ones under abiotic stresses: none (NAS), water (WS), heat (HS) or both stresses (WSHS). Comparisons were performed with Perseus software (Student T-test FDR < 0.05 and S0 = 0). Some lists are reduced to TOPs modulated compounds and the most specific ones selected by increasing the T-test stringency parameters (FDR and S0, specified in table legends) are highlighted in bold characters. Each compound is identified with an ID number. Measured *m/z* and calculated KEGG theoretical Mass and error (ppm) are indicated and the number of possible MassTrix identifications is indicated in the "n" column but only the first three ones are listed. Functional categories (Cat.) were manually determined according to KEGG annotations: Carbohydrates (Cb); Lipids (Lp); Phytochemicals (Ph); Peptides (Pp); Amino sugars (AmS); Nucleotides (Nu) and no match (NM). Functional categories counts are indicated in the table legends (Total and TOPs). Compounds are ranked according to their normalized value differences (up in green gradient and down in red gradient) between inoculated and non-inoculated samples. Identified isotopic compounds are indicated in italic characters.

IDENTIFIED *Np* COMPOUNDS

a) List of 33 identified "No abiotic stress" (NAS) *Np* vs NI (non-inoculated as control) from the significant 209 *m/z* at time B selected with T-test (FDR=0.05 and S0=0).
Total 33 functional category counts: Ph:15; Lp:5; Cb:6; Pp:1; AmS:2; Nu:2; NM:2.

ID	T-test *p-value*	NAS *Np*-NI	*m/z*	Mass	error (ppm)	n	Cat.	Formula	Identifications		
7463	0.002902	-2.882	609.188462	610.195738	0.997565	1	Ph	C34H30N2O9	Atalanine		
5540	0.001066	-2.815	472.146076	473.153353	0.058057	1	Ph	C20H27NO12	Proteacin		
1590	0.016934	-2.706	299.113594	300.120870	-0.109047	1	Cb	C14H20O7	Salidroside; p-Hydroxyphenethyl alcohol 1-O-β-D-glucoside; Tyrosol glucoside		
2931	0.031813	-2.544	351.127125	352.134401	-0.124492	3	Lp	C18H24O5S	Estradiol-17β 3-sulfate	Estradiol-17β 3-sulfate (see KEGG C08357); (8S,9S,13S,14S,17S)-17-hydroxy-13-methyl-3-sulfooxy-6,7,8,9,11,12,14,15,16,17-decahydrocyclopenta[a]phenanthrene [secondary alcohol]	Estradiol-17β 3-sulfate (see KEGG C08357); estra-1,3,5(10)-triene-3,17β-diol 3-sulfate [Sulfates [ST0502]]
1194	0.006606	-2.456	274.056804	275.064080	-0.131780	1	Ph	C10H13NO8	3-β-D-Glucopyranuronosyloxy-5-methylisoxazole		
2347	0.000273	-2.287	330.119400	331.126676	-0.122722	1	AmS	C14H21NO8	5'-O-β-D-Glucosylpyridoxine		
149	0.025849	-2.240	195.033221	196.040497	-0.242029	2	Cb	C6H12O5S	Thioglucoside	Thiogalactoside	
8451	0.018576	-2.217	721.416962	722.424238	0.153962	2	Ph	C39H62O12	Yamogenin 3-O-neohesperidoside	Ophiopogonin B	
3153	0.033093	-2.118	359.134719	360.141996	-0.102120	2	Ph	C16H24O9	7-Deoxyloganate	8-Epideoxyloganic acid	
2999	*0.011394*	-2.076	*353.124149*	*354.131425*	*0.362582*	*1*	*Nu*	*C16H20N2O7*	*O18 isotope of Cotinineglucuronide; 3,4,5-*		

									trihydroxy-6-[5-(1-methyl-5-oxo-pyrrolidin-2-yl)pyridin-1-yl]-oxane-2-carboxylic acid [cation]		
5002	0.007963	-1.848	441.189684	442.196960	0.399232	1	Lp	C18H35O10P	PG(6:/6:); 1,2-dihexanoyl-sn-glycero-3-phospho-(1'-sn-glycerol) [Diacylglycerophosphoglycerols [GP0401]]		
3775	0.049025	-1.846	382.100408	383.107685	-0.072174	1	Nu	C14H17N5O8	Succinyladenosine; (2S)-2-[[9-[(2S,3R,4S,5R)-3,4-dihydroxy-5-(hydroxymethyl)oxolan-2-yl]purin-6-yl]amino]butanedioic acid [primary alcohol]		
3454	0.022085	-1.838	369.082692	370.089969	-0.075560	1	Ph	C16H18O10	Fraxin		
2745	0.003371	-1.828	345.119057	346.126333	-0.142585	4	Lp	C15H22O9	Aucubin	Deutzioside	Aucubin (see KEGG C09771) [C10 isoprenoids (monoterpenes) [PR0102]]
1864	0.019281	-1.826	311.098334	312.105610	-0.117249	6	Cb	C11H20O10	Vicianose; O-α-L-Arabinopyranosyl-(1-6)-D-glucopyranose	Arabino-galactose	6-O-β-D-Xylopyranosyl-D-glucose; Primeverose
1635	*0.045664*	*-1.784*	*301.056465*	*302.063741*	*0.451381*	*1*	*NM*	*C16H13ClN2O2*	*Cl37 isotope of 3-(4-Chloro-benzyl)-5-(2-methoxy-phenyl)-[1,2,4]oxadiazole*		
3591	0.048769	-1.771	375.129648	376.136925	-0.059642	3	Ph	C16H24O10	Loganate; Loganic acid	7-Epiloganic acid	
2925	*0.039102*	*-1.713*	*351.085174*	*352.092450*	*0.281489*	*3*	*Lp*	*C18H23BrO2*	*O18 isotope of 16α-Bromo-17β-estradiol; 16α-Bromo-1,3,5(10)-estratriene-3,17β-diol*	*O18 isotope of 18-bromo-9E,17E-Octadecadiene-7,15-diynoic acid [Unsaturated fatty acids [FA0103]]*	*O18 isotope of 18-bromo-9Z,17E-Octadecadiene-7,15-diynoic acid [Unsaturated fatty acids [FA0103]]*
5361	0.000000	-1.692	461.202845	462.210121	0.020421	2	Cb	C21H34O11	Patrinoside	Valerosidatum; Valerosidate	
2804	0.020608	-1.688	347.116953	348.124230	0.459549	1	NM	C18H21ClN2O3	Compound VII; 5-(5-(6-Chloro-4-(4,5-dihydro-2-oxazolyl)phenoxy)pentyl)-3-methylisoxazole		
1730	*0.064316*	*-1.542*	*305.087764*	*306.095041*	*0.422086*	*2*	*Pp*	*C11H16N2O8*	*O18 isotope of N-Acetyl-aspartyl-glutamate; NAAG*	*O18 isotope of N-Acetylaspartylglutamic acid (see KEGG C12270); 2-[(2-acetamido-3-carboxy-propanoyl)amino]pentanedioic acid [carboxylic acid]*	
4335	0.009150	-1.527	405.119098	406.126375	-0.018591	7	Ph	C20H22O9	Astringin	Trichocarpin	Anadanthoside [Flavans, Flavanols and Leucoanthocyanidins [PK1202]]
1197	0.060528	-1.524	274.093187	275.100463	-0.141722	2	AmS	C11H17NO7	Cardiospermin	Sarmentosin; 2-Butenenitrile,4-(β-D-glucopyranosyloxy)-2-(hydroxymethyl)-,(E)-	
254	0.002153	-1.485	215.032742	216.040018	0.600053	1	Cb	C5H13O7P	2-C-Methyl-D-erythritol 4-phosphate		

3851	0.043273	-1.480	385.077611	386.084888	-0.061436	1	Ph	C16H18O11	O-Feruloylgalactarate; O-Feruloylgalactaric acid; 2'-(E)-O-Feruloylgalactaric acid		
3918	0.019872	-1.461	387.129645	388.136921	-0.066108	5	Cb	C17H24O10	Secologanin; (-)-Secologanin	Geniposide	Verbenalin; Cornin (glycoside)
3972	0.063208	-1.458	389.181684	390.188961	-0.055764	1	Lp	C18H30O9	(-)-11-hydroxy-9,10-dihydrojasmonic acid 11-β-D-glucoside; {(1R,2R)-2-[4-(β-D-glucopyranosyloxy)pentyl]-3-oxocyclopentyl}acetic acid [Jasmonic acids [FA0202]]		
199	0.009689	-1.402	205.035328	206.042604	-0.236504	7	Ph	C7H10O7	(R)-2-Hydroxybutane-1,2,4-tricarboxylate; Homocitrate; Homocitric acid; 3-Hydroxy-3-carboxyadipic acid; (R)-2-Hydroxy-1,2,4-butanetricarboxylic acid	2-Methylcitrate; 2-Hydroxybutane-1,2,3-tricarboxylate; (2R,3S)-2-Hydroxybutane-1,2,3-tricarboxylate	(2S,3R)-3-Hydroxybutane-1,2,3-tricarboxylate; Methylisocitrate; Methylisocitric acid
631	0.027726	-1.267	261.007409	262.014685	-0.147911	1	Ph	C9H10O7S	Homovanillic acid sulfate (see KEGG C05582) [alkyl aryl ether]		
3963	0.039111	-1.216	389.108911	390.116188	-0.061532	2	Ph	C16H22O11	Monotropein	Monotropein (see KEGG C09788) [C10 isoprenoids (monoterpenes) [PR0102]]	
2668	0.037964	-1.101	343.067029	344.074305	-0.120136	1	Ph	C14H16O10	Theogallin		
2127	0.067159	-0.433	321.134320	322.141596	-0.131584	2	Ph	C17H22O6	Tetraneurin A	epi-Tulipinolide diepoxide	
1219	0.042524	-0.349	275.092466	276.099742	-0.114667	5	Ph	C15H16O5	Lactucin	Vernolepin	Vernomenin

b) List of 33 identified WS *Np vs* NI (non-inoculated as control) from the significant 157 *m/z* at time B selected with T-test (FDR=0.05 and S0=0). Total 33 functional category counts: Ph:12; Lp:5; Cb:12; Pp:0; AmS:0; Nu:0; NM:4.

ID	T-test *p-value*	WS *Np*-NI	*m/z*	Mass	error (ppm)	n	Cat.	Formula	Identifications		
9	0.012805	+1.811	159.029884	160.037160	-0.082384	14	Lp	C6H8O5	2-Oxoadipate; 2-Oxoadipic acid	3-Oxoadipate; 3-Oxoadipic acid; 3-Keto-adipate	3D-(3,5/4)-Trihydroxycyclohexane-1,2-dione; D-2,3-Diketo-4-deoxy-epi-inositol; (3R,4S,5R)-3,4,5-Trihydroxy-1,2-cyclohexanedione; 3,5/4-Trihydroxycyclohexa-1,2-dione
66	0.002355	+1.802	177.040444	178.047720	-0.101633	28	Cb	C6H10O6	D-Glucono-1,5-lactone; Gluconic lactone; Gluconic acid lactone; 1,5-Gluconolactone; delta-Gluconolactone; D-Gluconolactone; Gluconolactone	2-Dehydro-3-deoxy-D-gluconate; 2-Keto-3-deoxy-D-gluconate	5-Dehydro-D-fructose
1191	0.000000	+1.674	273.170718	274.177995	-0.106789	2	Lp	C14H26O5	3-Hydroxytetradecanedioic acid; 3-hydroxy-tetradecanedioic acid [secondary alcohol]	3-hydroxy-tetradecanedioic acid; 3-hydroxy-tetradecanedioic acid [Dicarboxylic acids [FA0117]]	

8272	0.054397	+1.081	697.481468	698.488745	0.127128	14	Lp	C39H71O8P	PA(14:1(9Z)/22:2(13Z,16Z)); 1-(9Z-tetradecenoyl)-2-(13Z,16Z-docosadienoyl)-glycero-3-phosphate [Diacylglycerophosphates [GP1001]]	PA(16:1(9Z)/20:2(11Z,14Z)); 1-(9Z-hexadecenoyl)-2-(11Z,14Z-eicosadienoyl)-glycero-3-phosphate [Diacylglycerophosphates [GP1001]]	PA(17:2(9Z,12Z)/19:1(9Z)); 1-(9Z,12Z-heptadecadienoyl)-2-(9Z-nonadecenoyl)-glycero-3-phosphate [Diacylglycerophosphates [GP1001]]
631	0.002411	-2.932	261.007409	262.014685	-0.147911	1	Ph	C9H10O7S	Homovanillic acid sulfate (see KEGG C05582) [alkyl aryl ether]		
2804	0.005229	-2.512	347.116953	348.124230	0.459549	1	NM	C18H21ClN2O3	Compound VII; 5-(5-(6-Chloro-4-(4,5-dihydro-2-oxazolyl)phenoxy)pentyl)-3-methylisoxazole		
6424	0.013195	-2.455	523.145770	524.153046	0.105903	1	Ph	C24H28O13	Barbatoflavan [Flavans, Flavanols and Leucoanthocyanidins [PK1202]]		
2668	0.047706	-2.182	343.067029	344.074305	-0.120136	1	Ph	C14H16O10	Theogallin		
1635	*0.000450*	*-2.136*	*301.056465*	*302.063741*	*0.451381*	*1*	*NM*	*C16H13ClN2O2*	*Cl37 isotope of 3-(4-Chloro-benzyl)-5-(2-methoxy-phenyl)-[1,2,4]oxadiazole*		
3594	*0.000056*	*-2.029*	*375.166033*	*376.173310*	*0.410054*	*1*	*NM*	*C21H27ClN2O2*	*Cl37 isotope of Hydroxyzine*		
517	0.015029	-1.987	249.080184	250.087460	-0.138795	2	Lp	C10H18O5S	2-(5'-Methylthio)pentylmalic acid; 2-(5'-Methylthio)pentylmalate	3-(5'-Methylthio)pentylmalic acid; 3-(5'-Methylthio)pentylmalate	
1194	0.007014	-1.939	274.056804	275.064080	-0.131780	1	Ph	C10H13NO8	3-β-D-Glucopyranuronosyloxy-5-methylisoxazole		
7463	0.004680	-1.736	609.188462	610.195738	0.997565	1	Ph	C34H30N2O9	Atalanine		
3500	0.000835	-1.727	371.134722	372.141998	-0.092343	1	Ph	C17H24O9	Syringin; Eleutheroside B		
3454	0.003527	-1.708	369.082692	370.089969	-0.075560	1	Ph	C16H18O10	Fraxin		
2222	0.039798	-1.654	325.113981	326.121258	-0.120525	6	Cb	C12H22O10	α-L-Fucosyl-1,2-β-D-galactoside; Glycoprotein α-L-fucosyl-(1,2)-D-galactose; Glycolipid α-L-fucosyl-1,2-β-D-galactose; α-L-Fucosyl-(1->2)-D-galactosyl-R	2-O-α-L-Rhamnopyranosyl-D-glucopyranose; Neohesperidose	Robinobiose
3101	0.013537	-1.646	357.119069	358.126345	-0.103262	3	Cb	C16H22O9	Tarennoside	10-Deoxygeniposidic acid	Sweroside
2389	0.052927	-1.636	331.176183	332.183459	-0.132727	2	Lp	C16H28O7	(2S,3S)-2-Hydroxytridecane-1,2,3-tricarboxylate	(2S,3S)-2-hydroxytridecane-1,2,3-tricarboxylic acid (see KEGG C04655); (2S,3S)-2-hydroxytridecane-1,2,3-tricarboxylic acid [Dicarboxylic acids [FA0117]]	
2224	0.002050	-1.630	325.129232	326.136508	-0.138273	1	NM	C16H22O7	Hinokitiol glucoside; β-Thujaplicin 2-O-β-D-glucoside		
2373	0.013486	-1.628	331.067028	332.074304	-0.128768	1	Cb	C13H16O10	1-O-Galloyl-β-D-glucose; 1-Galloyl-β-glucose; β-Glucogallin; 1-Galloyl-β-D-glucose		
2074	0.027394	-1.610	319.139804	320.147080	-0.116812	2	Cb	C14H24O8	Valproic acid glucuronide (see KEGG C03033); 3,4,5-trihydroxy-6-(2-	Octanoylglucuronide (see KEGG C03033) [secondary alcohol]	

									propylpentanoyloxy)oxane-2-carboxylic acid [secondary alcohol]		
4335	0.004527	-1.559	405.119098	406.126375	-0.018591	7	Ph	C20H22O9	Astringin	Trichocarpin	Anadanthoside [Flavans, Flavanols and Leucoanthocyanidins [PK1202]]
3591	0.010757	-1.521	375.129648	376.136925	-0.059642	3	Ph	C16H24O10	Loganate; Loganic acid	7-Epiloganic acid	
5934	0.013027	-1.485	493.098797	494.106073	0.065861	31	Ph	C22H22O13	Pleurostimin 7-glucoside; 5,6,7,3',4'-Pentahydroxy-8-methoxyflavone 7-glucoside [Flavones and Flavonols [PK1211]]	Laricitrin 3-galactoside [Flavones and Flavonols [PK1211]]	Laricitrin 3-glucoside [Flavones and Flavonols [PK1211]]
2920	0.008095	-1.456	351.056857	352.064133	-0.122128	2	Cb	C12H16O12	4-(4-Deoxy-β-D-gluc-4-enuronosyl)-D-galacturonate	4-(4-Deoxy-α-D-gluc-4-enuronosyl)-D-galacturonate; 4-(4-Deoxy-α-D-galact-4-enuronosyl)-D-galacturonate; Unsaturated digalacturonate	
3977	0.001828	-1.236	389.218068	390.225344	-0.061128	2	Cb	C19H34O8	Rehmaionoside A	Rehmaionoside B	
7699	0.012660	-1.208	633.073451	634.080728	0.180016	1	Ph	C27H22O18	Corilagin		
3540	0.039047	-1.067	373.113993	374.121269	-0.074217	4	Ph	C16H22O10	Secologanate	Swertiamarin	Geniposidic acid
1864	0.039079	-1.066	311.098334	312.105610	-0.117249	6	Cb	C11H20O10	Vicianose; O-α-L-Arabinopyranosyl-(1-6)-D-glucopyranose	Arabino-galactose	6-O-β-D-Xylopyranosyl-D-glucose; Primeverose
5732	0.053416	-1.039	483.078040	484.085316	0.022519	1	Cb	C20H20O14	1-O,6-O-Digalloyl-β-D-glucose		
2811	0.043362	-1.020	347.171101	348.178377	-0.116791	1	Cb	C16H28O8	Schizonepetoside E		
3926	0.002504	-0.928	387.202414	388.209691	-0.070880	5	Cb	C19H32O8	Rehmaionoside C	methyl 9-hydroperoxy-10,12,13,15-bisepidioxy-16E-octadecenoate [Hydroperoxy fatty acids [FA0104]]	methyl 10,12,13,15-bisepidioxy-16-hydroperoxy-8E-octadecenoate [Hydroperoxy fatty acids [FA0104]]
1590	0.028032	-0.798	299.113594	300.120870	-0.109047	1	Cb	C14H20O7	Salidroside; p-Hydroxyphenethyl alcohol 1-O-β-D-glucoside; Tyrosol glucoside		

c) List of TOP 41 compounds identified HS *Np vs* NI (non-inoculated as control) from the significant 583 *m/z* at time A selected with T-test (FDR=0.01 and S0=1). Total: 131 annotated compounds (128-/3+): Total 131 structure category counts: Ph:16; Lp:20; Cb:2; Pp:0; AmS:1; Nu:0; NM:2. The most specific compounds selected with higher stringency (FDR=0.08 and S0=1) are indicated in bolded lines. TOPs 41 functional category counts: Ph:66; Lp:18; Cb:33; Pp:3; AmS:3; Nu:1; NM:7.

**ID**	**T-test *p-value***	**HS *Np*-NI**	***m/z***	**Mass**	**error (ppm)**	**n**	**Cat.**	**Formula**	**Identifications**		
**93**	0.000183	**+1.920**	**181.050603**	**182.057880**	**-0.160171**	**17**	**Ph**	**C9H10O4**	**3-(4-Hydroxyphenyl)lactate; 4-Hydroxyphenyllactate; p-Hydroxyphenyllactate; 2-Hydroxy-3-(4-hydroxyphenyl)propanoate**	**(R)-3-(4-Hydroxyphenyl)lactate**	**3-(2,3-Dihydroxyphenyl)propanoate; 2,3-Dihydroxyphenylpropanoate**
8177	0.010349	+1.845	685.481404	686.488680	0.035433	14	Lp	C38H71O8P	PA(13:0/22:2(13Z,16Z)); 1-tridecanoyl-2-(13Z,16Z-	PA(15:0/20:2(11Z,14Z)); 1-pentadecanoyl-2-(11Z,14Z-	PA(15:1(9Z)/20:1(11Z)); 1-(9Z-pentadecenoyl)-2-(11Z-

									docosadienoyl)-glycero-3-phosphate [Diacylglycerophosphates [GP1001]]	eicosadienoyl)-glycero-3-phosphate [Diacylglycerophosphates [GP1001]]	eicosenoyl)-glycero-3-phosphate [Diacylglycerophosphates [GP1001]]
**2579**	0.001213	**-2.455**	**339.129643**	**340.136919**	**-0.081507**	**1**	**Cb**	**C13H24O10**	**Methyl-2-α-L-fucopyranosyl-β-D-galactoside**		
**1677**	0.002185	**-2.438**	**303.050993**	**304.058270**	**-0.108829**	**7**	**Ph**	**C15H12O7**	**Taxifolin; Dihydroquercetin; (+)-Dihydroquercetin; (+)-Taxifolin; *trans*-Dihydroquercetin**	**Pentahydroxyflavanone; Dihydrotricetin**	***cis*-Dihydroquercetin**
2347	0.044133	-2.354	330.119400	331.126676	-0.122722	1	AmS	C14H21NO8	5'-O-β-D-Glucosylpyridoxine		
5361	0.034364	-2.318	461.202845	462.210121	0.020421	2	Cb	C21H34O11	Patrinoside	Valerosidatum; Valerosidate	
1158	0.011871	-2.272	271.061181	272.068458	-0.058348	37	Ph	C15H12O5	Naringenin; 4',5,7-Trihydroxyflavanone	(6aS,11aS)-3,6a,9-Trihydroxypterocarpan; 3,6,9-Trihydroxypterocarpan; (-)-Glycinol	Licodione
**4814**	0.004251	**-2.258**	**431.155905**	**432.163181**	**0.044900**	**1**	**Cb**	**C19H28O11**	**Zizybeoside I**		
11	0.007951	-2.180	161.045532	162.052809	-0.091525	30	Cb	C6H10O5	Lichenin; Lichenan	(R)-3,3-Dimethylmalate	3-Ethylmalate
65	0.013675	-2.147	177.019326	178.026603	-0.033633	4	Ph	C9H6O4	7,8-Dihydroxycoumarin; Daphnetin	5,7-Dihydroxychromone	Esculetin; Aesculetin
5431	0.008132	-2.144	465.103882	466.111159	0.069908	6	Ph	C21H22O12	Quercetin 7-O-glucoside	Plantagoside	3,4,5,2',4',6'-Hexahydroxychalcone 2'-glucoside [Chalcones and dihydrochalcones [PK1212]]
51	0.011332	-2.107	173.009145	174.016422	-0.092226	6	Ph	C6H6O6	*cis*-Aconitate; *cis*-Aconitic acid	*trans*-Aconitate; *trans*-Aconitic acid	Dehydroascorbate; Dehydroascorbic acid; L-Dehydroascorbate; L-Dehydroascorbic acid
**2893**	0.000054	**-2.104**	**350.306446**	**351.313723**	**-0.019531**	**1**	**Lp**	**C22H41NO2**	**Anandamide (20:2, n-6); N-(11Z,14Z-eicosadienoyl)-ethanolamine [N-acyl ethanolamines (endocannabinoids) [FA0804]]**		
928	0.013838	-2.092	263.128852	264.136128	-0.116493	33	Ph	C15H20O4	Abscisate; Abscisic acid; (+)-Abscisic acid	Amaralin	Artemisiifolin
**4627**	0.002328	**-2.091**	**421.114055**	**422.121331**	**0.081091**	**7**	**Ph**	**C20H22O10**	**Catechin 7-O-β-D-xyloside**	**Plicatic acid**	**Catechin 7-O-β-D-xyloside [Flavans, Flavanols and Leucoanthocyanidins [PK1202]]**
4027	*0.005346*	*-2.088*	*391.160971*	*392.168248*	*0.453424*	*1*	*NM*	*C21H27ClN2O3*	*Cl37 isotope of Compound II(R/S); 5-(7-(6-Chloro-4-(5-*		

									hydro-4-methyl-2-oxazolyl)phenoxy)heptyl)-3-methylisoxazole		
3049	0.023822	-2.081	355.103413	356.110690	-0.119119	3	Ph	C16H20O9	Gentiopicrin; Gentiopicroside	1-O-Feruloyl-β-D-glucose; 1-Feruloyl-D-glucose	Gentiopicrin (see KEGG C09782) [C10 isoprenoids (monoterpenes) [PR0102]]
2277	0.020385	-2.081	327.163515	328.170791	-0.119791	1	Lp	C17H28O4S	Steryl sulfate		
4847	0.021465	-2.075	433.114041	434.121318	0.048061	32	Ph	C21H22O10	Naringenin 7-O-β-D-glucoside; Prunin	Phlorizin chalcone; Chalcone 2'-O-glucoside	2',4,4',6'-Tetrahydroxychalcone 4'-O-glucoside; THC 4'-glucoside
2506	0.012550	-2.069	336.129968	337.137244	-0.110462	3	AmS	C13H23NO9	Streptobiosamine		
3153	0.016177	-2.052	359.134719	360.141996	-0.102120	2	Ph	C16H24O9	7-Deoxyloganate	8-Epideoxyloganic acid	
**5064**	0.001825	**-2.048**	**445.190118**	**446.197394**	**-0.100135**	**1**	**Lp**	**C21H34O8S**	**Ptilosteroid A; 5α-pregn-20-en-2β,3β,4β,15β,16β-pentol 2-sulfate [C21 steroids (gluco/mineralocorticoids, progestogins) and derivatives [ST0203]]**		
3922	0.003605	-2.042	387.166034	388.173310	-0.057526	2	Lp	C18H28O9	Tuberonic acid glucoside	Tuberonic acid glucoside (see KEGG C08558); (1R,2S)-3-oxo-2-(5'-O-β-D-glucopyranosyloxy-2'Z-pentenyl)-cyclopentaneacetic acid [Fatty acyl glycosides of mono- and disaccharides [FA1301]]	
5133	0.007019	-2.027	449.108952	450.116229	0.038519	25	Ph	C21H22O11	Neoastilbin; (2S,3S)-Taxifolin 3-rhamnoside	2',3,4,4',6'-Peptahydroxychalcone 4'-O-glucoside; PHC 4'-O-glucoside	Astilbin
**80**	0.000435	**-1.984**	**179.056091**	**180.063368**	**-0.114407**	**69**	**Cb**	**C6H12O6**	**D-Glucose; Grape sugar; Dextrose**	**D-Fructose; Levulose; Fruit sugar; D-arabino-Hexulose**	**D-Galactose**
4511	0.005081	-1.967	413.218120	414.225397	0.069673	3	Lp	C21H34O8	methyl 5-hydroperoxy-6,8,9,11-bisepidioxy-12,14-eicosadienoate [Hydroperoxy fatty acids [FA0104]]	methyl 9,11-epidioxy-12,15-dihydroperoxy-5,7,13-eicosatrienoate [Hydroxy/hydroperoxyeicosatrienoic acids [FA0305]]	methyl 6,8-epidioxy-5,15-dihydroperoxy-9,11,13-eicosatrienoate [Hydroxy/hydroperoxyeicosatrienoic acids [FA0305]]
8870	0.005483	-1.918	833.518322	834.525598	-0.277170	23	Lp	C43H79O13P	PI(16:0/18:2(9Z,12Z)) (see KEGG C00626); 1-hexadecanoyl-2-(9Z,12Z-octadecadienoyl)-sn-glycero-3-phosphoinositol [secondary alcohol]	PI(16:1(9Z)/18:1(11Z)) (see KEGG C00626); 1-(9Z-hexadecenoyl)-2-(11Z-octadecenoyl)-sn-glycero-3-phosphoinositol [secondary alcohol]	PI(16:1(9Z)/18:1(9Z)) (see KEGG C00626); 1-(9Z-hexadecenoyl)-2-(9Z-octadecenoyl)-sn-glycero-3-phosphoinositol [secondary alcohol]
**7603**	0.000011	**-1.906**	**623.255794**	**624.263070**	**0.216834**	**1**	**Cb**	**C27H44O16**	**Kanokoside D**		

6367	0.010584	-1.879	519.187226	520.194503	0.078545	2	Ph	$C_{26}H_{32}O_{11}$	Brusatol	(-)-Pinoresinol glucoside	
1303	0.006311	-1.835	281.139425	282.146702	-0.078970	6	Ph	$C_{15}H_{22}O_{5}$	Hymenoxon	Qing Hau Sau; Artemisinin	Dihydrophaseic acid; (-)-Dihydrophaseic acid
3972	0.001427	-1.830	389.181684	390.188961	-0.055764		Lp	$C_{18}H_{30}O_{9}$	(-)-11-hydroxy-9,10-dihydrojasmonic acid 11-β-D-glucoside; {(1R,2R)-2-[4-(β-D-glucopyranosyloxy)pentyl]-3-oxocyclopentyl}acetic acid [Jasmonic acids [FA0202]]		
4021	0.007441	-1.828	391.124562	392.131838	-0.059958	1	Ph	$C_{16}H_{24}O_{11}$	Shanzhiside		
2745	0.009898	-1.822	345.119057	346.126333	-0.142585	4	Lp	$C_{15}H_{22}O_{9}$	Aucubin	Deutzioside	Aucubin (see KEGG C09771) [C10 isoprenoids (monoterpenes) [PR0102]]
449	0.009663	-1.808	243.066250	244.073527	-0.131492	10	Ph	$C_{14}H_{12}O_{4}$	3,3',4'5-Tetrahydroxystilbene; Piceatannol	Wyerone acid	Eriobofuran
**6042**	0.000000	**-1.751**	**499.291301**	**500.298577**	**0.088261**	**1**	**NM**	**$C_{26}H_{44}O_{9}$**	**Mupirocin**		
3508	0.001932	-1.751	371.243903	372.251179	-0.025932	5	Lp	$C_{20}H_{36}O_{6}$	8,8a-Deoxyoleandolide	19(R)-hydroxy-PGF1α; 9S,11R,15S,19R-tetrahydroxy-13E-prostaenoic acid [Prostaglandins [FA0301]]	13,14-dihydro-19(R)-hydroxyPGE1; 9-oxo-11R,15S,19R-trihydroxy-prostanoic acid [Prostaglandins [FA0301]]
4022	0.007294	*-1.742*	*391.128449*	*392.135726*	*0.030165*	*5*	*Ph*	*$C_{20}H_{22}O_{8}$*	*O18 isotope of Piceid; Polydatin; 3,4,5-Trihydroxystilbene-3-β-monoglucoside*	*O18 isotope of Populin*	*O18 isotope of Apigeniflavan 5-O-xyloside [Flavans, Flavanols and Leucoanthocyanidins [PK1202]]*
5691	0.006981	-1.739	481.098785	482.106061	0.042399	1	Ph	$C_{21}H_{22}O_{13}$	3,4,2',3',4',6',α-Heptahydroxychalcone 2'-glucoside [Chalcones and dihydrochalcones [PK1212]]		
4329	0.001465	-1.712	405.082709	406.089985	-0.028041	7	Ph	$C_{19}H_{18}O_{10}$	Lancerin; 4-C-Glucosyl-1,3,7-trihydroxyxanthone	5,7,3',5'-Tetrahydroxy-3,6,8,4'-tetramethoxyflavone [Flavones and Flavonols [PK1211]]	5,7,3',4'-Tetrahydroxy-3,6,8,5'-tetramethoxyflavone [Flavones and Flavonols [PK1211]]
4594	0.004359	-1.690	419.134784	420.142061	0.067492	10	Ph	$C_{21}H_{24}O_{9}$	Melampodin A	Glycyphyllin; Phloretin 2'-O-rhamnoside	Isorhapontin
2276	0.002669	-1.688	327.144911	328.152188	-0.047159	3	Ph	$C_{16}H_{24}O_{7}$	Rhododendrin	Tetranor-PGEM; 11R-hydroxy-9,15-dioxo-2,3,4,5-tetranor-prostan-1,20-dioic acid [Prostaglandins [FA0301]]	Prostaglandin M; 7R-hydroxy-5,11-dioxotetranorprostane-1,16-dioic acid [Prostaglandins [FA0301]]

d) List of TOP 39 identified WSHS *Np vs* NI (non-inoculated as control) from the significant 882 *m/z* at time A selected with T-test (FDR=0.0001 and S0=3). Total: 187 annotated compounds (181-/6+): Total 187 functional category counts: Ph:102; Lp:25; Cb:37; Pp:6; AmS:6; Nu:1; NM:10. TOPs 39 functional category counts: Ph:19; Lp:5; Cb:9; Pp:2; AmS:0; Nu:0; NM:4.

ID	T-test *p-value*	WSHS *Np*-NI	*m/z*	Mass	error (ppm)	n	Cat.	Formula	Identifications		
502	*0.00252*	*+3.023*	*247.047368*	*248.054644*	*-0.228030*	*1*	*Lp*	*C6H15O8P*	*O18 isotope of Glycerophosphoglycerol*		
2474	*0.008504*	*+2.930*	*335.063417*	*336.070694*	*-0.151416*	*5*	*Cb*	*C9H19O11P*	*O18 isotope of sn-glycero-3-Phospho-1-inositol; 1-(sn-glycero-3-Phospho)-1D-myo-inositol*	*O18 isotope of 2-(α-D-Galactosyl)-sn-glycerol 3-phosphate*	*O18 isotope of α-D-Galactosyl-(1,1')-sn-glycerol 3-phosphate; 1-O-α-D-Galactosyl-sn-glycerol 3-phosphate*
7699	0.000388	-2.997	633.073451	634.080728	0.180016	1	Ph	C27H22O18	Corilagin		
5781	0.001096	-2.818	485.108961	486.116238	0.054370	2	Ph	C24H22O11	Flavonol 3-O-(6-O-malonyl-β-D-glucoside)	Epigallocatechin 3-O-(3,5-di-O-methylgallate) [Flavans, Flavanols and Leucoanthocyanidins [PK1202]]	
4393	0.014393	-2.795	407.171139	408.178415	-0.005805	2	Ph	C21H28O8	Vernoflexuoside		
4717	0.032512	-2.791	425.087827	426.095103	0.049636	2	Ph	C22H18O9	Epiafzelechin 3-O-gallate [Flavans, Flavanols and Leucoanthocyanidins [PK1202]]	Epigallocatechin 3-O-(4-hydroxybenzoate) [Flavans, Flavanols and Leucoanthocyanidins [PK1202]]	
1087	*0.000557*	*-2.766*	*265.144542*	*266.151818*	*0.677275*	*6*	*Pp*	*C14H20N2O3*	*O18 isotope of Subaphyllin; Feruloylputrescine*	*Mukaadial*	*Rugosal*
4985	0.015744	-2.728	441.082837	442.090114	0.264769	10	Ph	C22H18O10	Robinetinidol 3-O-gallate [Flavans, Flavanols and Leucoanthocyanidins [PK1202]]	ent-Robinetinidol 3-O-gallate [Flavans, Flavanols and Leucoanthocyanidins [PK1202]]	Catechin 3-O-gallate [Flavans, Flavanols and Leucoanthocyanidins [PK1202]]
5457	0.022641	-2.645	467.098394	468.105671	0.051411	2	Ph	C24H20O10	Epigallocatechin 3-O-caffeate [Flavans, Flavanols and Leucoanthocyanidins [PK1202]]	Apocynin A [Flavans, Flavanols and Leucoanthocyanidins [PK1202]]	
291	0.007195	-2.619	221.066628	222.073904	-0.220142	4	Cb	C8H14O7	6-Acetyl-D-glucose	6-Acetyl-β-D-galactoside	Ethyl glucuronide; (2S,3S,4S,5R)-6-ethoxy-3,4,5-trihydroxyoxane-2-carboxylic acid [acetal]
2222	0.002763	-2.601	325.113981	326.121258	-0.120525	6	Cb	C12H22O10	α-L-Fucosyl-1,2-β-D-galactoside; Glycoprotein α-L-fucosyl-(1,2)-D-galactose; Glycolipid α-L-fucosyl-1,2-β-D-galactose; α-L-Fucosyl-(1->2)-D-galactosyl-R	2-O-α-L-Rhamnopyranosyl-D-glucopyranose; Neohesperidose	Robinobiose
1130	0.000774	-2.584	269.045564	270.052841	0.064429	42	Ph	C15H10O5	Apigenin; 4',5,7-Trihydroxyflavone; 5,7,4'-Trihydroxyflavone	2'-Hydroxydaidzein	Genistein; 5,7,4'-Trihydroxyisoflavone
1622	0.011095	-2.589	300.998953	302.006230	-0.124868	2	Ph	C14H6O8	Ellagic acid	Ellagic acid (see KEGG C10788) [phenol or hydroxyhetarene]	
1135	0.002842	-2.546	269.081904	270.089181	-0.104475	35	Lp	C16H14O4	(+)-Medicarpin; 3-Hydroxy-9-methoxypterocarpan	Vignafuran	Alloimperatorin
4289	0.007513	-2.539	403.124570	404.131847	-0.036646	2	Ph	C17H24O11	Gardenoside	Scandoside methyl ester	

233	0.008054	-2.480	211.133944	212.141220	-0.115861	15	Lp	C12H20O3	Cucurbic acid	Putaminoxin	Traumatin; (10E)-12-Oxododecenoic acid
1843	0.000007	-2.474	310.093192	311.100468	-0.108516	4	Ph	C14H17NO7	Taxiphyllin; (R)-4-Hydroxymandelonitrile β-D-glucoside	Dhurrin; (S)-4-Hydroxymandelonitrile β-D-glucoside	p-Glucosyloxymandelonitrile
5778	*0.000002*	*-2.458*	*485.082492*	*486.089768*	*0.446498*	*1*	*Cb*	*C20H20O14*	*O18 isotope of 1-O,6-O-Digalloyl-β-D-glucose*		
3121	0.005300	-2.451	358.114330	359.121606	-0.070431	1	Pp	C15H21NO9	Epinephrine glucuronide (see KEGG C03033); (2S,3S,4S,6S)-3,4,5,5-tetrahydroxy-3-[(1R)-1-hydroxy-2-methylaminoethyl]-6-(phenoxy)oxane-2-carboxylic acid [acetal]		
4441	0.001599	-2.412	409.150412	410.157688	0.014166	2	Ph	C20H26O9	Bruceine D	C20H26O9	
132	0.000032	-2.410	191.034952	192.042228	-0.159394	10	Ph	C10H8O4	Scopoletin	Naphthazarin; 5,8-Dihydroxy-1,4-naphthoquinone	3,4-Dehydro-6-hydroxymellein
2684	0.000005	-2.383	343.124543	344.131820	-0.121434	4	Cb	C12H24O11	Melibiitol; 6-O-α-D-Galactosyl-D-glucitol	Clusianose	Maltitol; 4-[3,4,5-trihydroxy-6-(hydroxymethyl)oxan-2-yl]oxyhexane-1,2,3,5,6-pentol [acetal]
3969	*0.007159*	*-2.378*	*389.145306*	*390.152582*	*0.415595*	*4*	*NM*	*C21H25ClN2O3*	*Cl37 isotope of Cetirizine*	*Cl37 isotope of Cetirizine (see KEGG C07778); 2-[2-[4-[(4-chlorophenyl)-phenyl-methyl]piperazin-1-yl]ethoxy]acetic acid [dialkyl ether]*	*Loganin*
3099	*0.000347*	*-2.369*	*357.107697*	*358.114974*	*-0.013607*	*4*	*Ph*	*C16H20O9*	*O18 isotope of Gentiopicrin; Gentiopicroside*	*O18 isotope of 1-O-Feruloyl-β-D-glucose; 1-Feruloyl-D-glucose*	*O18 isotope of Gentiopicrin (see KEGG C09782) [C10 isoprenoids (monoterpenes) [PR0102]]*
6042	0.000214	-2.337	499.291301	500.298577	0.088261	1	NM	C26H44O9	Mupirocin		
4635	0.002498	-2.320	421.186827	422.194104	0.084740	4	Ph	C22H30O8	Valtratum; Valtrate	Isovaltrate	Picrasin F
3088	0.001545	-2.319	357.061579	358.068856	-0.032332	10	Ph	C18H14O8	5,3'-Dihydroxy-4',5'-dimethoxy-6,7-methylenedioxyisoflavone [Isoflavonoids [PK1205]]	Dalpalatin [Isoflavonoids [PK1205]]	Luteolin 7-lactate [Flavones and Flavonols [PK1211]]
6216	0.003172	-2.313	511.218562	512.225838	0.149097	1	Ph	C25H36O11	gibberellin A2 O-β-D-glucoside [C20 isoprenoids (diterpenes) [PR0104]]		
1138	0.001137	-2.284	269.175813	270.183090	-0.072009	1	Lp	C15H26O4	(10S)-Juvenile hormone III acid diol		
2893	0.001210	-2.258	350.306446	351.313723	-0.019531	1	Lp	C22H41NO2	Anandamide (20:2, n-6); N-(11Z,14Z-eicosadienoyl)-ethanolamine [N-acyl ethanolamines (endocannabinoids) [FA0804]]		
4730	0.000106	-2.237	425.181737	426.189014	0.073384	2	Ph	C21H30O9	Abscisic acid glucose ester; abscisic acid-β-D-glucopyranosyl ester	γ-CEHC Glc; γ-carboxyethyl-hydroxychroman β-D-glucoside [Vitamin E [PR0202]]	
11	0.000069	-2.234	161.045532	162.052809	-0.091525	30	Cb	C6H10O5	Lichenin; Lichenan	(R)-3,3-Dimethylmalate	3-Ethylmalate
3918	0.000909	-2.229	387.129645	388.136921	-0.066108	5	Cb	C17H24O10	Secologanin; (-)-Secologanin	Geniposide	Verbenalin; Cornin (glycoside)

306	0.000004	-2.204	223.133928	224.141204	-0.180408	8	Ph	C13H20O3	Vomifoliol; (6S,9R)-6-hydroxy-3-oxo-α-ionol	(+/-)-6-Hydroxy-3-oxo-α-ionol	Methyl jasmonate; (-)-Methyl jasmonate
6299	0.000011	-2.172	515.286219	516.293495	0.092665	2	Lp	C26H44O10	L-Olivosyl-oleandolide	L-Olivosyl-oleandolide (see KEGG C11991) [Macrolides and lactone polyketides [PK04]]	
1869	0.000018	-2.162	313.147866	314.155142	0.528679	1	NM	C19H23ClN2	Clomipramine		
1252	0.000042	-2.153	277.144506	278.151783	-0.095744	7	NM	C16H22O4	2-Ethylhexyl phthalate	Dibutyl phthalate; Di-n-butyl phthalate; Dibutyl 1,2-benzenedicarboxylate	Diisobutyl phthalate; DIBP
4597	0.000028	-2.126	419.155889	420.163166	0.009385	2	Ph	C18H28O11	Lamioside	Lamioside (see KEGG C11645) [C10 isoprenoids (monoterpenes) [PR0102]]	
3598	0.000034	-2.111	375.202440	376.209716	-0.005151	1	Cb	C18H32O8	2,3-di--hexanoyl-α-glucopyranose [Other acyl sugars [SL05]]		

TABLES IDENTIFIED *Ds* COMPOUNDS

e) List of 25 identified "No abiotic stress" (NAS) *Ds* vs NI (non-inoculated as control) from the significant 135 *m/z* at time B selected with T-test (FDR=0.05 and S0=0). Total 25 functional category counts: Ph:12; Lp:4; Cb:7; Pp:0; AmS:1; Nu:1; NM:0.

ID	T-test *p-value*	NAS *Ds*-NI	*m/z*	Mass	error (ppm)	n	Cat.	Formula	Identifications		
7463	0.004622	-2.686	609.188462	610.195738	0.997565	1	Ph	C34H30N2O9	Atalanine		
3591	0.022328	-2.367	375.129648	376.136925	-0.059642	3	Ph	C16H24O10	Loganate; Loganic acid	7-Epiloganic acid	
5540	0.019670	-2.302	472.146076	473.153353	0.058057	1	Ph	C20H27NO12	Proteacin		
3851	0.001480	-2.301	385.077611	386.084888	-0.061436	1	Ph	C16H18O11	O-Feruloylgalactarate; O-Feruloylgalactaric acid; 2'-(E)-O-Feruloylgalactaric acid		
2347	0.000273	-2.287	330.119400	331.126676	-0.122722	1	AmS	C14H21NO8	5'-O-β-D-Glucosylpyridoxine		
3153	0.022575	-2.279	359.134719	360.141996	-0.102120	2	Ph	C16H24O9	7-Deoxyloganate	8-Epideoxyloganic acid	
1590	0.035859	-2.233	299.113594	300.120870	-0.109047	1	Cb	C14H20O7	Salidroside; p-Hydroxyphenethyl alcohol 1-O-β-D-glucoside; Tyrosol glucoside		
2681	*0.042487*	*-2.173*	*343.113128*	*344.120404*	*-0.156273*	*45*	*Cb*	*C12H22O11*	*O18 isotope of Sucrose; Cane sugar; Saccharose; 1-α-D-Glucopyranosyl-2-β-D-fructofuranoside*	*O18 isotope of Cellobiose; 1-β-D-Glucopyranosyl-4-D-glucopyranose*	*O18 isotope of Maltose; Malt sugar; α-D-Glucopyranosyl-(1->4)-D-glucopyranose*
2745	0.004061	-2.156	345.119057	346.126333	-0.142585	4	Lp	C15H22O9	Aucubin	Deutzioside	Aucubin (see KEGG C09771) [C10 isoprenoids (monoterpenes) [PR0102]]
2614	0.027651	-1.971	341.108884	342.116161	-0.149462	45	Cb	C12H22O11	Sucrose; Cane sugar; Saccharose; 1-α-D-Glucopyranosyl-2-β-D-fructofuranoside	Cellobiose; 1-β-D-Glucopyranosyl-4-D-glucopyranose	Maltose; Malt sugar; α-D-Glucopyranosyl-(1->4)-D-glucopyranose

3775	0.045960	-1.871	382.100408	383.107685	-0.072174	1	Nu	C14H17N5O8	Succinyladenosine; (2S)-2-[[9-[(2S,3R,4S,5R)-3,4-dihydroxy-5-(hydroxymethyl)oxolan-2-yl]purin-6-yl]amino]butanedioic acid [primary alcohol]		
1864	0.018181	-1.860	311.098334	312.105610	-0.117249	6	Cb	C11H20O10	Vicianose; O-α-L-Arabinopyranosyl-(1-6)-D-glucopyranose	Arabino-galactose	6-O-β-D-Xylopyranosyl-D-glucose; Primeverose
8272	0.003918	-1.639	697.481468	698.488745	0.127128	14	Lp	C39H71O8P	PA(14:1(9Z)/22:2(13Z,16Z)); 1-(9Z-tetradecenoyl)-2-(13Z,16Z-docosadienoyl)-glycero-3-phosphate [Diacylglycerophosphates [GP1001]]	PA(16:1(9Z)/20:2(11Z,14Z)); 1-(9Z-hexadecenoyl)-2-(11Z,14Z-eicosadienoyl)-glycero-3-phosphate [Diacylglycerophosphates [GP1001]]	PA(17:2(9Z,12Z)/19:1(9Z)); 1-(9Z,12Z-heptadecadienoyl)-2-(9Z-nonadecenoyl)-glycero-3-phosphate [Diacylglycerophosphates [GP1001]]
8293	0.021010	-1.567	699.497125	700.504401	0.135904	20	Lp	C39H73O8P	PA(18:/18:2(9Z,12Z)) (see KEGG C00416); 1-octadecanoyl-2-(9Z,12Z-octadecadienoyl)-sn-glycero-3-phosphate [carboxylic acid ester]	PA(18:1(11Z)/18:1(11Z)) (see KEGG C00416); 1,2-di(11Z-octadecenoyl)-rac-glycero-3-phosphate [carboxylic acid ester]	PA(18:1(11Z)/18:1(9Z)) (see KEGG C00416); 1-(11Z-octadecenoyl)-2-(9Z-octadecenoyl)-sn-glycero-3-phosphate [carboxylic acid ester]
1194	0.036655	-1.551	274.056804	275.064080	-0.131780	1	Ph	C10H13NO8	3-β-D-Glucopyranuronosyloxy-5-methylisoxazole		
3101	0.018617	-1.520	357.119069	358.126345	-0.103262	3	Cb	C16H22O9	Tarennoside	10-Deoxygeniposidic acid	Sweroside
414	0.005905	-1.488	239.056069	240.063345	-0.178947	1	Ph	C11H12O6	(1R,6R)-6-Hydroxy-2-succinylcyclohexa-2,4-diene-1-carboxylate; (1R,6R)-2-Succinyl-6-hydroxy-2,4-cyclohexadiene-1-carboxylate; SHCHC		
3454	0.001419	-1.415	369.082692	370.089969	-0.075560	1	Ph	C16H18O10	Fraxin		
5069	0.000031	-1.191	445.368738	446.376015	0.043363	4	Lp	C29H50O3	13'-hydroxy-α-tocopherol [primary alcohol]	Nebrosteroid M; 4α-methyl-24-methylene-cholestan-3β,8β,11β-triol [Ergosterols and C24-methyl derivatives [ST0103]]	24-hydroperoxy-24-vinylcholesterol; 24-hydroperoxy-24-vinyl-cholest-5-en-3β-ol [Stigmasterols and C24-ethyl derivatives [ST0104]]
2373	0.027657	-0.876	331.067028	332.074304	-0.128768	1	Cb	C13H16O10	1-O-Galloyl-β-D-glucose; 1-Galloyl-β-glucose; β-Glucogallin; 1-Galloyl-β-D-glucose		
2668	0.040378	-0.818	343.067029	344.074305	-0.120136	1	Ph	C14H16O10	Theogallin		
2222	0.036017	-0.753	325.113981	326.121258	-0.120525	6	Cb	C12H22O10	α-L-Fucosyl-1,2-β-D-galactoside; Glycoprotein α-L-fucosyl-(1,2)-D-galactose; Glycolipid α-L-fucosyl-1,2-β-D-galactose; α-L-Fucosyl-(1->2)-D-galactosyl-R	2-O-α-L-Rhamnopyranosyl-D-glucopyranose; Neohesperidose	Robinobiose
1219	0.024786	-0.494	275.092466	276.099742	-0.114667	5	Ph	C15H16O5	Lactucin	Vernolepin	Vernomenin
4380	0.034810	-0.351	407.098363	408.105640	-0.017139	1	Ph	C19H20O10	Khellol glucoside; Khelloside		
2127	0.023288	-0.294	321.134320	322.141596	-0.131584	2	Ph	C17H22O6	Tetraneurin A	epi-Tulipinolide diepoxide	

f) List of TOP 80 identified HS *Ds vs* NI (non-inoculated ascontrol) from the significant 538 *m/z* at time A selected with T-test (FDR=0.01 and S0=1). Total: 128 annotated compounds (121-/7+): Total 128 functional category counts: Ph:52; Lp:9; Cb:14; Pp:0; AmS:1; Nu:1; NM:3. The most specific compounds selected with higher stringency (FDR=0.005 and S0=1) are indicated in bolded lines. TOPs 80 functional category counts: Ph:73; Lp:15; Cb:29; Pp:2; AmS:3; Nu:1; NM:5.

ID	T-test *p-value*	HS *Ds*-NI	*m/z*	Mass	error (ppm)	n	Cat.	Formula	Identifications		
4953	0.024454	+2.652	439.124589	440.131866	0.009877	2	Ph	C20H24O11	Ginkgolide C; BN 52022	Ginkgolide C (see KEGG C07603) [C20 isoprenoids (diterpenes) [PR0104]]	
4380	0.038096	+2.449	407.098363	408.105640	-0.017139	1	Ph	C19H20O10	Khellol glucoside; Khelloside		
1219	0.030303	+2.380	275.092466	276.099742	-0.114667	5	Ph	C15H16O5	Lactucin	Vernolepin	Vernomenin
1788	0.027959	+2.052	307.118678	308.125954	-0.110488	3	Ph	C16H20O6	14-Dihydroxycornestin; Cornexistin	Monocerin	
**2467**	**0.001702**	**+2.033**	**335.040834**	**336.048111**	**-0.990151**	**1**	**Nu**	**C11H15NO9P**	**Nicotinate D-ribonucleotide; β-Nicotinate D-ribonucleotide; Nicotinate ribonucleotide; Nicotinic acid ribonucleotide**		
1918	0.058023	-2.620	313.147866	314.155142	0.528679	1	NM	C19H23ClN2	Clomipramine		
**1677**	**0.071333**	**-2.589**	**303.050993**	**304.058270**	**-0.108829**	**7**	**Ph**	**C15H12O7**	**Taxifolin; Dihydroquercetin; (+)-Dihydroquercetin; (+)-Taxifolin; *trans*-Dihydroquercetin**	**Pentahydroxyflavanone; Dihydrotricetin**	***cis*-Dihydroquercetin**
**5431**	**0.039450**	**-2.508**	**465.103882**	**466.111159**	**0.069908**	**6**	**Ph**	**C21H22O12**	**Quercetin 7-O-glucoside**	**Plantagoside**	**3,4,5,2',4',6'-Hexahydroxychalcone 2'-glucoside [Chalcones and dihydrochalcones [PK1212]]**
1590	*0.031800*	-2.490	299.113594	300.120870	-0.109047	1	Cb	C14H20O7	Salidroside; p-Hydroxyphenethyl alcohol 1-O-β-D-glucoside; Tyrosol glucoside		
2057	0.046867	-2.427	319.045905	320.053181	-0.113324	3	Ph	C15H12O8	Dihydromyricetin; Ampelopsin; (+)-Dihydromyricetin; (+)-Ampelopsin	2,3-Dihydrogossypetin	Amaronol A; (+)- 2,4,6-Trihydroxy-2-[(3,4,5-trihydroxyphenyl)methyl]-3(2H)-benzofuranone [Aurone flavonoids [PK1213]]
**478**	**0.006533**	**-2.395**	**245.081895**	**246.089171**	**-0.153334**	**6**	**Ph**	**C14H14O4**	**Columbianetin**	**Decursinol**	**Marmesin; (+)-Marmesin**
1409	0.065922	-2.383	289.071731	290.079007	-0.106598	35	Ph	C15H14O6	*cis*-3,4-Leucopelargonidin	Luteoforol; 3-Deoxyleucocyanidin	(+)-Catechin; D-Catechin; Cyanidanol; (2R-*trans*)-2-(3,4-dihydroxyphenyl)-3,4-dihydro-2H-1-Benzopyran-3,5,7-triol; (2R,3S)-Catechin; (2R,3S)-(+)-Catechin; Cianidanol
**5133**	**0.069413**	**-2.371**	**449.108952**	**450.116229**	**0.038519**	**25**	**Ph**	**C21H22O11**	**Neoastilbin; (2S,3S)-Taxifolin 3-rhamnoside**	**2',3,4,4',6'-Peptahydroxychalcone 4'-O-glucoside; PHC 4'-O-glucoside**	**Astilbin**
4847	0.088631	-2.358	433.114041	434.121318	0.048061	32	Ph	C21H22O10	Naringenin 7-O-β-D-glucoside; Prunin	Phlorizin chalcone; Chalcone 2'-O-glucoside	2',4,4',6'-Tetrahydroxychalcone 4'-O-glucoside; THC 4'-glucoside
1186	0.069386	-2.356	273.076829	274.084105	-0.067655	26	Ph	C15H14O5	Phloretin	α-Pyrufuran	β-Pyrufuran
5493	0.031666	-2.297	469.135176	470.142452	0.055729	1	Ph	C21H26O12	Plumieride		

2347	0.016412	-2.288	330.119400	331.126676	-0.122722	1	AmS	C14H21NO8	5'-O-β-D-Glucosylpyridoxine		
2277	0.070602	-2.285	327.163515	328.170791	-0.119791	1	Lp	C17H28O4S	Steryl sulfate		
1158	0.039929	-2.272	271.061181	272.068458	-0.058348	37	Ph	C15H12O5	Naringenin; 4',5,7-Trihydroxyflavanone	(6aS,11aS)-3,6a,9-Trihydroxypterocarpan; 3,6,9-Trihydroxypterocarpan; (-)-Glycinol	Licodione
1346	0.05955	-2.212	285.040436	286.047712	-0.089823	34	Ph	C15H10O6	Luteolin; 3',4',5,7-Tetrahydroxyflavone; 5,7,3',4'-Tetrahydroxyflavone	Kaempferol; 3,4',5,7-Tetrahydroxyflavone; 4H-1-Benzopyran-4-one,3,5,7-trihydroxy-2-(4-hydroxyphenyl)-5,7,4'-Trihydroxyflavonol; C.I. 75640; Indigo yellow; Kaempherol; Kempferol; Nimbecetin; Pelargidenolon; Populnetin; Rhamnolutein; Rhamnolutin; Robigenin; Swartziol; Trifolitin	Aureusidin
7597	0.053253	-2.207	623.161749	624.169025	-0.015889	60	Ph	C28H32O16	Tectorigenin 7-O-gentiobioside [Isoflavonoids [PK1205]]	Isorhamnetin 3-neohesperidoside [Flavones and Flavonols [PK1211]]	Narcissin [Flavones and Flavonols [PK1211]]
3049	0.030753	-2.183	355.103413	356.110690	-0.119119	3	Ph	C16H20O9	Gentiopicrin; Gentiopicroside	1-O-Feruloyl-β-D-glucose; 1-Feruloyl-D-glucose	Gentiopicrin (see KEGG C09782) [C10 isoprenoids (monoterpenes) [PR0102]]
4887	0.064083	-2.146	435.129686	436.136963	0.036110	16	Ph	C21H24O10	Phlorizin; Phlorhizin; Phloridzin	Epiafzelechin 3-O-β-D-allopyranoside [Flavans, Flavanols and Leucoanthocyanidins [PK1202]]	Arthromerin B [Flavans, Flavanols and Leucoanthocyanidins [PK1202]]
1376	0.049557	-2.130	287.056089	288.063365	-0.079954	33	Ph	C15H12O6	Dihydrokaempferol; Aromadendrin; (+)-Dihydrokaempferol; (+)-Aromadendrin	Fustin; 2,3-Dihydrofisetin	Eriodictyol
5691	0.065446	-2.127	481.098785	482.106061	0.042399	1	Ph	C21H22O13	3,4,2',3',4',6',α-Heptahydroxychalcone 2'-glucoside [Chalcones and dihydrochalcones [PK1212]]		
**2893**	**0.055781**	**-2.104**	**350.306446**	**351.313723**	**-0.019531**	**1**	**Lp**	**C22H41NO2**	**Anandamide (20:2, n-6); N-(11Z,14Z-eicosadienoyl)-ethanolamine [N-acyl ethanolamines (endocannabinoids) [FA0804]]**		
**4627**	**0.000164**	**-2.091**	**421.114055**	**422.121331**	**0.081091**	**7**	**Ph**	**C20H22O10**	**Catechin 7-O-β-D-xyloside**	**Plicatic acid**	**Catechin 7-O-β-D-xyloside [Flavans, Flavanols and Leucoanthocyanidins [PK1202]]**
2745	0.004778	-2.084	345.119057	346.126333	-0.142585	4	Lp	C15H22O9	Aucubin	Deutzioside	Aucubin (see KEGG C09771) [C10 isoprenoids (monoterpenes) [PR0102]]
3088	0.046723	-2.079	357.061579	358.068856	-0.032332	10	Ph	C18H14O8	5,3'-Dihydroxy-4',5'-dimethoxy-6,7-methylenedioxyisoflavone [Isoflavonoids [PK1205]]	Dalpalatin [Isoflavonoids [PK1205]]	Luteolin 7-lactate [Flavones and Flavonols [PK1211]]

**5064**	**0.032395**	**-2.048**	**445.190118**	**446.197394**	**-0.100135**	**1**	**Lp**	**C21H34O8S**	**Ptilosteroid A; 5α-pregn-20-en-2β,3β,4β,15β,16β-pentol 2-sulfate [C21 steroids (gluco/mineralocorticoids, progestogins) and derivatives [ST0203]]**		
3153	0.075825	-2.043	359.134719	360.141996	-0.102120	2	Ph	C16H24O9	7-Deoxyloganate	8-Epideoxyloganic acid	
2579	0.067408	-2.037	339.129643	340.136919	-0.081507	1	Cb	C13H24O10	Methyl-2-α-L-fucopyranosyl-β-D-galactoside		
3154	0.025590	-2.025	359.149987	360.157263	-0.070751	11	Ph	C20H24O6	Antheridiogen-An	Triptolide	Euponin
2111	0.002536	-2.019	321.061550	322.068827	-0.126928	3	Ph	C15H14O8	Leucodelphinidin	Elephantorrhizol [Flavans, Flavanols and Leucoanthocyanidins [PK1202]]	Gallocatechin-4β-ol [Flavans, Flavanols and Leucoanthocyanidins [PK1202]]
3857	0.048409	-1.998	385.113996	386.121273	-0.063172	2	Ph	C17H22O10	1-O-Sinapoyl-β-D-glucose; 1-O-Sinapoyl β-D-glucoside	4-O-β-D-Glucosyl-sinapate	
328	0.000022	-1.992	227.071321	228.078597	-0.205916	10	Ph	C14H12O3	3,4',5-Trihydroxystilbene; Resveratrol	Seselin	Trioxsalen; Trioxysalen; 4,8,5'-Trimethylpsoralen
1722	0.003721	-1.980	305.066640	306.073916	-0.120233	11	Ph	C15H14O7	Leucocyanidin; 2-(3,4-Dihydroxyphenyl)-3,4-dihydro-2H-1-benzopyran-3,4,5,7-tetrol; 3,3',4,4',5,7-Flavanhexol; 3,4-Cyanidiol; Leucoanthocyanidol; Leucocianidol; Leucocyanidol; Leukocyanidine; Procyanidol; Resivit; 2,3-*trans*-3,4-*cis*-Leucocyanidin	(+)-Gallocatechin; Gallocatechol	(-)-Epigallocatechin
1255	*0.000667*	-1.977	277.217271	278.224548	-0.117541	62	Lp	C18H30O2	(6Z,9Z,12Z)-Octadecatrienoic acid; 6,9,12-Octadecatrienoic acid; γ-Linolenic acid; Gamolenic acid	(9Z,12Z,15Z)-Octadecatrienoic acid; α-Linolenic acid; 9,12,15-Octadecatrienoic acid; Linolenate; α-Linolenate	Crepenynate; (9Z)-Octadec-9-en-12-ynoate; (Z)-9-Octadecen-12-ynoic acid; Crepenynic acid
5457	0.052513	-1.967	467.098394	468.105671	0.051411	2	Ph	C24H20O10	Epigallocatechin 3-O-caffeate [Flavans, Flavanols and Leucoanthocyanidins [PK1202]]	Apocynin A [Flavans, Flavanols and Leucoanthocyanidins [PK1202]]	
51	0.002089	-1.967	173.009145	174.016422	-0.092226	6	Ph	C6H6O6	*cis*-Aconitate; *cis*-Aconitic acid	*trans*-Aconitate; *trans*-Aconitic acid	Dehydroascorbate; Dehydroascorbic acid; L-Dehydroascorbate; L-Dehydroascorbic acid
3101	0.051993	-1.932	357.119069	358.126345	-0.103262	3	Cb	C16H22O9	Tarennoside	10-Deoxygeniposidic acid	Sweroside
1247	0.071058	-1.922	277.092868	278.100145	-0.082506	5	Ph	C11H18O8	Tuliposide A	Isovalerylglucuronide (see KEGG C03033); (2S,3S,4S,5R,6S)-3,4,5-trihydroxy-6-(3-methylbutanoyloxy)oxane-2 carboxylic acid [secondary alcohol]	
**7603**	**0.048851**	**-1.906**	**623.255794**	**624.263070**	**0.216834**	**1**	**Cb**	**C27H44O16**	**Kanokoside D**		
1304	0.040718	-1.898	281.248574	282.255850	-0.106353	58	Lp	C18H34O2	(9Z)-Octadecenoic acid; (Z)-Octadec-9-enoic acid; Oleate; Oleic acid	(9E)-Octadecenoic acid; 9-Octadecenoic acid, (E)-; (E)-Oleic acid; D9-*trans*-Octadecenoic acid; 9-*trans*-Octadecenoic acid; *trans*-D9-	(6Z)-Octadecenoic acid; Petroselinic acid; *cis*-6-Octadecenoic acid

										Octadecenoic acid; *trans*-9-Octadecenoic acid; *trans*-Elaidic acid; *trans*-Oleic acid; Elaidic acid; Acide elaidique; Elaidinsaure	
8583	0.004363	-1.884	743.486985	744.494261	0.169877	18	Lp	C40H73O10P	PG(16:/18:3(6Z,9Z,12Z)); 1-hexadecanoyl-2-(6Z,9Z,12Z-octadecatrienoyl)-sn-glycero-3-phospho-(1'-sn-glycerol) [primary alcohol]	PG(16:/18:3(9Z,12Z,15Z)); 1-hexadecanoyl-2-(9Z,12Z,15Z-octadeatrienoyl)-sn-glycero-3-phospho-(1'-sn-glycerol) [primary alcohol]	PG(16:1(9Z)/18:2(9Z,12Z)); 1-(9Z-hexadecenoyl)-2-(9Z,12Z-octadecadienoyl)-sn-glycero-3-phospho-(1'-sn-glycerol) [primary alcohol]
4814	0.052664	-1.857	431.155905	432.163181	0.044900	1	Cb	C19H28O11	Zizybeoside I		
5036	0.080100	-1.831	443.192275	444.199552	0.009678	1	Cb	C21H32O10	dihydrophaseic acid 4-O-β-D-glucoside; (2Z,4E)-5-[(1R,3S,5R,8S)-3-(β-D-glucopyranosyloxy)-8-hydroxy-1,5-dimethyl-6-oxabicyclo[3.2.1]oct-8-yl]-3-methylpenta-2,4-dienoic acid [C15 isoprenoids (sesquiterpenes) [PR0103]]		
4021	0.027272	-1.828	391.124562	392.131838	-0.059958	1	Ph	C16H24O11	Shanzhiside		
449	0.023042	-1.808	243.066250	244.073527	-0.131492	10	Ph	C14H12O4	3,3',4'5-Tetrahydroxystilbene; Piceatannol	Wyerone acid	Eriobofuran
5171	0.056776	-1.806	451.103475	452.110751	0.041592	6	Ph	C24H20O9	Cinchonain 1a	9,10-Dihydro-10-(3,4-dihydroxyphenyl)-pyrano[2,3-h]catechin-8-one [Flavans, Flavanols and Leucoanthocyanidins [PK1202]]	Cinchonain Ib [Flavans, Flavanols and Leucoanthocyanidins [PK1202]]
4027	*0.047126*	*-1.795*	*391.160971*	*392.168248*	*0.453424*	*1*	*NM*	*C21H27ClN2O3*	*Cl37 isotope of Compound II(R/S); 5-(7-(6-Chloro-4-(5-hydro-4-methyl-2-oxazolyl)phenoxy)heptyl)-3-methylisoxazole*		
2219	0.012638	-1.754	325.092849	326.100126	-0.128228	8	Ph	C15H18O8	4-O-β-D-Glucosyl-4-hydroxycinnamate	*trans*-β-D-Glucosyl-2-hydroxycinnamate; β-D-Glucosyl-2-coumarate	*cis*-β-D-Glucosyl-2-hydroxycinnamate; β-D-Glucosyl-2-coumarinate
**6042**	**0.001942**	**-1.751**	**499.291301**	**500.298577**	**0.088261**	**1**	**NM**	**C26H44O9**	**Mupirocin**		
4022	*0.001195*	*-1.742*	*391.128449*	*392.135726*	*0.030165*	*5*	*Ph*	*C20H22O8*	*O18 isotope of Piceid; Polydatin; 3,4,5-Trihydroxystilbene-3-β-monoglucoside*	*O18 isotope of Populin*	*O18 isotope of Apigeniflavan 5-O-xyloside [Flavans, Flavanols and Leucoanthocyanidins [PK1202]]*
2179	0.049689	-1.738	323.098329	324.105606	-0.127735	2	Cb	C12H20O10	Bis-D-fructose 2',1:2,1'-dianhydride; Difructose anhydride I; Di-D-fructose 1,2'; 2,1' dianhydride	D-Fructofuranose 1,2':2,3'-dianhydride	
4636	0.050494	-1.720	421.207924	422.215201	0.008100	1	Cb	C19H34O10	1-Octen-3-ol-3-o-β-D-xylopyranosyl(1->6)-β-D-glucopyranoside		
6367	0.048713	-1.714	519.187226	520.194503	0.078545	2	Ph	C26H32O11	Brusatol	(-)-Pinoresinol glucoside	
11	0.079341	-1.706	161.045532	162.052809	-0.091525	30	Cb	C6H10O5	Lichenin; Lichenan	(R)-3,3-Dimethylmalate	3-Ethylmalate

4393	0.077835	-1.694	407.171139	408.178415	-0.005805	2	Ph	C21H28O8	Vernoflexuoside		
4594	0.003211	-1.690	419.134784	420.142061	0.067492	10	Ph	C21H24O9	Melampodin A	Glycyphyllin; Phloretin 2'-O-rhamnoside	Isorhapontin
2276	0.028060	-1.688	327.144911	328.152188	-0.047159	3	Ph	C16H24O7	Rhododendrin	Tetranor-PGEM; 11R-hydroxy-9,15-dioxo-2,3,4,5-tetranor-prostan-1,20-dioic acid [Prostaglandins [FA0301]]	Prostaglandin M; 7R-hydroxy-5,11-dioxotetranorprostane-1,16-dioic acid [Prostaglandins [FA0301]]
4292	0.052111	-1.672	403.139861	404.147137	0.048967	4	Ph	C21H24O8	Mallotophenone	Koaburanin [Flavans, Flavanols and Leucoanthocyanidins [PK1202]]	Orientanol A [Pterocarpans [PK1207]]
6989	0.000813	-1.658	563.140743	564.148019	0.201658	52	Ph	C26H28O14	Apigenin 7-O-[β-D-apiosyl-(1->2)-β-D-glucoside]; 7-O-(β-D-Apiofuranosyl-1,2-β-D-glucosyl)-5,7,4'-trihydroxyflavone; Apiin; 7-[(2-O-D-Apio-β-D-furanosyl-β-D-glucopyranosyl)oxy]-5-hydroxy-2-(4-hydroxy-phenyl)-4H-1-benzopyran-4-one; Apioside	Neoschaftoside	Schaftoside
3591	0.049406	-1.657	375.129648	376.136925	-0.059642	3	Ph	C16H24O10	Loganate; Loganic acid	7-Epiloganic acid	
2539	*0.038221*	*-1.650*	*337.113983*	*338.121259*	*0.392818*	*1*	*Ph*	*C12H20N2O9*	*O18 isotope of 3-Epihydroxymugineic acid*		
3972	0.000701	-1.622	389.181684	390.188961	-0.055764	1	Lp	C18H30O9	(-)-11-hydroxy-9,10-dihydrojasmonic acid 11-β-D-glucoside; {(1R,2R)-2-[4-(β-D-glucopyranosyloxy)pentyl]-3-oxocyclopentyl}acetic acid [Jasmonic acids [FA0202]]		
7153	0.006694	-1.619	577.135234	578.142510	0.145859	12 2	Ph	C30H26O12	Epicatechin-(4β->8)-ent-epicatechin	Procyanidin B4; Catechin-(4α->8)-epicatechin	Procyanidin B2; Procyanidol B2
3967	0.00036	-1.597	389.124168	390.131444	-0.060382	5	Ph	C20H22O8	Piceid; Polydatin; 3,4,5-Trihydroxystilbene-3-β-monoglucoside	Populin	Apigeniflavan 5-O-xyloside [Flavans, Flavanols and Leucoanthocyanidins [PK1202]]
1480	0.046372	-1.569	293.030257	294.037533	-0.115504	1	Ph	C13H10O8	Tricrozarin A		
1303	0.050254	-1.544	281.139425	282.146702	-0.078970	6	Ph	C15H22O5	Hymenoxon	Qing Hau Sau; Artemisinin	Dihydrophaseic acid; (-)-Dihydrophaseic acid
80	0.006086	-1.538	179.056091	180.063368	-0.114407	69	Cb	C6H12O6	D-Glucose; Grape sugar; Dextrose	D-Fructose; Levulose; Fruit sugar; D-arabino-Hexulose	D-Galactose
1351	0.000212	-1.532	285.097947	286.105223	-0.104355	4	Cb	C13H18O7	Salicin; Salicoside	Gastrodin; 4-(Hydroxymethyl)phenyl-β-D-glucopyranoside	Methylarbutin
5778	*7.93E-08*	*-1.528*	*485.082492*	*486.089768*	*0.446498*	*1*	*Cb*	*C20H20O14*	*O18 isotope of 1-O,6-O-Digalloyl-β-D-glucose*		
3922	0.035977	-1.497	387.166034	388.173310	-0.057526	2	Lp	C18H28O9	Tuberonic acid glucoside	Tuberonic acid glucoside (see KEGG C08558); (1R,2S)-3-oxo-2-(5'-O-β-D-glucopyranosyloxy-2'Z-pentenyl)-cyclopentaneacetic acid [Fatty acyl glycosides of	

										mono- and disaccharides [FA1301]]	
6824	0.011486	-1.451	549.182543	550.189820	0.089857	1	Ph	C23H34O15	Genipin 1-β-gentiobioside		
6097	0.005081	-1.407	503.161797	504.169074	0.077257	21	Cb	C18H32O16	Raffinose; Melitose; Melitriose; Gossypose; 6G-α-D-galactosylsucrose	Panose	Galactomannan; Galactomannoglycan
7699	0.011577	-1.389	633.073451	634.080728	0.180016	1	Ph	C27H22O18	Corilagin		
1586	0.023494	-1.363	299.077209	300.084485	-0.108396	1	Ph	C13H16O8	4-(β-D-Glucosyloxy)benzoate		
4335	0.082859	-1.358	405.119098	406.126375	-0.018591	7	Ph	C20H22O9	Astringin	Trichocarpin	Anadanthoside [Flavans, Flavanols and Leucoanthocyanidins [PK1202]]
5732	0.068787	-1.325	483.078040	484.085316	0.022519	1	Cb	C20H20O14	1-O,6-O-Digalloyl-β-D-glucose		

g) List of TOP 51 identified WSHS *Ds vs* NI (non-inoculated as control) from the significant 802 *m/z* at time A selected with T-test (FDR=0.005 and S0=2). Total: 174 annotated compounds (173-/1+): Total 174 functional category counts: Ph:31; Lp:8; Cb:8; Pp:1; AmS:2; Nu:0; NM:1. The most specific compounds selected with higher stringency (FDR=0.001 and S0=2) are indicated in bolded lines. TOPs 51 functional category counts: Ph:91; Lp:26; Cb:37; Pp:6; AmS:4; Nu:0; NM:10.

**ID**	**T-test *p-value***	**WSHS *Ds*-NI**	***m/z***	**Mass**	**error (ppm)**	**n**	**Cat.**	**Formula**	**Identifications**		
**5781**	**0.023383**	**-2.818**	**485.108961**	**486.116238**	**0.054370**	**2**	**Ph**	**C24H22O11**	**Flavonol 3-O-(6-O-malonyl-β-D-glucoside)**	**Epigallocatechin 3-O-(3,5-di-O-methylgallate) [Flavans, Flavanols and Leucoanthocyanidins [PK1202]]**	
1087	***0.116299***	***-2.766***	***265.144542***	***266.151818***	***0.677275***	***6***	***Pp***	***C14H20N2O3***	***O18 isotope of Subaphyllin; Feruloylputrescine***	***Mukaadial***	***Rugosal***
**5566**	**0.021056**	**-2.662**	**473.363676**	**474.370953**	**0.090535**	**15**	**Ph**	**C30H50O4**	**Sapelin A**	**Bryodulcosigenin**	**Soyasapogenol A**
4717	0.000313	-2.655	425.087827	426.095103	0.049636	2	Ph	C22H18O9	Epiafzelechin 3-O-gallate [Flavans, Flavanols and Leucoanthocyanidins [PK1202]]	Epigallocatechin 3-O-(4-hydroxybenzoate) [Flavans, Flavanols and Leucoanthocyanidins [PK1202]]	
**1130**	**0.038652**	**-2.584**	**269.045564**	**270.052841**	**0.064429**	**42**	**Ph**	**C15H10O5**	**Apigenin; 4',5,7-Trihydroxyflavone; 5,7,4'-Trihydroxyflavone**	**2'-Hydroxydaidzein**	**Genistein; 5,7,4'-Trihydroxyisoflavone**
7699	0.068581	-2.575	633.073451	634.080728	0.180016	1	Ph	C27H22O18	Corilagin		
5278	0.081839	-2.563	457.077691	458.084967	0.122575	7	Ph	C22H18O11	Epigallocatechin 3-gallate	8-Demethyltetracenomycin C	Epigallocatechin gallate (see KEGG C09731); [(2R,3R)-5,7-dihydroxy-2-(3,4,5-trihydroxyphenyl)chroman-3-yl] 3,4,5-trihydroxybenzoate [phenol or hydroxyhetarene]
**233**	**0.034641**	**-2.480**	**211.133944**	**212.141220**	**-0.115861**	**15**	**Lp**	**C12H20O3**	**Cucurbic acid**	**Putaminoxin**	**Traumatin; (10E)-12-Oxododecenoic acid**
5457	0.036977	-2.465	467.098394	468.105671	0.051411	2	Ph	C24H20O10	Epigallocatechin 3-O-caffeate [Flavans, Flavanols and Leucoanthocyanidins [PK1202]]	Apocynin A [Flavans, Flavanols and Leucoanthocyanidins [PK1202]]	
3970	0.029056	-2.462	389.160552	390.167829	-0.062778	2	Ph	C21H26O7	Erioflorin acetate		

5778	*0.056400*	*-2.458*	*485.082492*	*486.089768*	*0.446498*	*1*	*Cb*	*C20H20O14*	*O18 isotope of 1-O,6-O-Digalloyl-β-D-glucose*		
**132**	**0.116164**	**-2.410**	**191.034952**	**192.042228**	**-0.159394**	**10**	**Ph**	**C10H8O4**	**Scopoletin**	**Naphthazarin; 5,8-Dihydroxy-1,4-naphthoquinone**	**3,4-Dehydro-6-hydroxymellein**
3099	***0.000109***	***-2.369***	***357.107697***	***358.114974***	***-0.013607***	***4***	***Ph***	***C16H20O9***	***O18 isotope of Gentiopicrin; Gentiopicroside***	***O18 isotope of 1-O-Feruloyl-β-D-glucose; 1-Feruloyl-D-glucose***	***O18 isotope of Gentiopicrin (see KEGG C09782) [C10 isoprenoids (monoterpenes) [PR0102]]***
3831	0.000344	-2.353	384.145333	385.152609	0.187448	1	Ph	C21H23NO6	Polycarpine		
6042	0.076656	-2.337	499.291301	500.298577	0.088261	1	NM	C26H44O9	Mupirocin		
**1843**	**0.058426**	**-2.335**	**310.093192**	**311.100468**	**-0.108516**	**4**	**Ph**	**C14H17NO7**	**Taxiphyllin; (R)-4-Hydroxymandelonitrile β-D-glucoside**	**Dhurrin; (S)-4-Hydroxymandelonitrile β-D-glucoside**	**p-Glucosyloxymandelonitrile**
**4635**	**0.018222**	**-2.320**	**421.186827**	**422.194104**	**0.084740**	**4**	**Ph**	**C22H30O8**	**Valtratum; Valtrate**	**Isovaltrate**	**Picrasin F**
**3088**	**0.022141**	**-2.319**	**357.061579**	**358.068856**	**-0.032332**	**10**	**Ph**	**C18H14O8**	**5,3'-Dihydroxy-4',5'-dimethoxy-6,7-methylenedioxyisoflavone [Isoflavonoids [PK1205]]**	**Dalpalatin [Isoflavonoids [PK1205]]**	**Luteolin 7-lactate [Flavones and Flavonols [PK1211]]**
5262	0.023223	-2.317	455.353108	456.360385	0.086530	20	Ph	C30H48O3	Betulinic acid	α-Elemolic acid	Ursolic acid
**4511**	**0.029686**	**-2.304**	**413.218120**	**414.225397**	**0.069673**	**3**	**Lp**	**C21H34O8**	**methyl 5-hydroperoxy-6,8,9,11-bisepidioxy-12,14-eicosadienoate [Hydroperoxy fatty acids [FA0104]]**	**methyl 9,11-epidioxy-12,15-dihydroperoxy-5,7,13-eicosatrienoate [Hydroxy/hydroperoxyeicosatrienoic acids [FA0305]]**	**methyl 6,8-epidioxy-5,15-dihydroperoxy-9,11,13-eicosatrienoate [Hydroxy/hydroperoxyeicosatrienoic acids [FA0305]]**
4985	0.045130	-2.295	441.082837	442.090114	0.264769	10	Ph	C22H18O10	Robinetinidol 3-O-gallate [Flavans, Flavanols and Leucoanthocyanidins [PK1202]]	ent-Robinetinidol 3-O-gallate [Flavans, Flavanols and Leucoanthocyanidins [PK1202]]	Catechin 3-O-gallate [Flavans, Flavanols and Leucoanthocyanidins [PK1202]]
2893	0.072813	-2.258	350.306446	351.313723	-0.019531	1	Lp	C22H41NO2	Anandamide (20:2, n-6); N-(11Z,14Z-eicosadienoyl)-ethanolamine [N-acyl ethanolamines (endocannabinoids) [FA0804]]		
3591	0.076360	-2.248	375.129648	376.136925	-0.059642	3	Ph	C16H24O10	Loganate; Loganic acid	7-Epiloganic acid	
4688	0.013266	-2.246	423.326893	424.334170	0.092124	4	Lp	C29H44O2	α-Tocotrienol	4,4'-Methylenebis(2,6-di-tert-butylphenol)	α-Tocotrienol (see KEGG C14153); (2R)-2,5,7,8-tetramethyl-2-[(3E,7E)-4,8,12-trimethyltrideca-3,7,11-trienyl]chroman-6-ol [phenol or hydroxyhetarene]
**4730**	**0.023538**	**-2.237**	**425.181737**	**426.189014**	**0.073384**	**2**	**Ph**	**C21H30O9**	**Abscisic acid glucose ester; abscisic acid-β-D-glucopyranosyl ester**	**γ-CEHC Glc; γ-carboxyethyl-hydroxychroman β-D-glucoside [Vitamin E [PR0202]]**	
**2684**	**0.022418**	**-2.216**	**343.124543**	**344.131820**	**-0.121434**	**4**	**Cb**	**C12H24O11**	**Melibiitol; 6-O-α-D-Galactosyl-D-glucitol**	**Clusianose**	**Maltitol; 4-[3,4,5-trihydroxy-6-(hydroxymethyl)oxan-2-yl]oxyhexane-1,2,3,5,6-pentol [acetal]**
5171	0.006168	-2.211	451.103475	452.110751	0.041592	6	Ph	C24H20O9	Cinchonain 1a	9,10-Dihydro-10-(3,4-dihydroxyphenyl)-pyrano[2,3-h]catechin-8-one [Flavans,	Cinchonain Ib [Flavans, Flavanols and Leucoanthocyanidins [PK1202]]

										Flavanols and Leucoanthocyanidins [PK1202]]	
4636	0.008933	-2.209	421.207924	422.215201	0.008100	1	Cb	C19H34O10	1-Octen-3-ol-3-o-β-D-xylopyranosyl(1->6)-β-D-glucopyranoside		
**306**	**0.033012**	**-2.204**	**223.133928**	**224.141204**	**-0.180408**	**8**	**Ph**	**C13H20O3**	**Vomifoliol; (6S,9R)-6-hydroxy-3-oxo-α-ionol**	**(+/-)-6-Hydroxy-3-oxo-α-ionol**	**Methyl jasmonate; (-)-Methyl jasmonate**
928	0.084980	-2.200	263.128852	264.136128	-0.116493	33	Ph	C15H20O4	Abscisate; Abscisic acid; (+)-Abscisic acid	Amaralin	Artemisiifolin
1583	0.055928	-2.176	299.056112	300.063389	0.002400	77	Ph	C16H12O6	5,7,4'-Trihydroxy-3'-methoxyflavone; 3'-O-Methylluteolin; Chrysoeriol	3-Methoxyapigenin; 3-O-Methylkaempferol	Leptosidin
**6299**	**0.003170**	**-2.172**	**515.286219**	**516.293495**	**0.092665**	**2**	**Lp**	**C26H44O10**	**L-Olivosyl-oleandolide**	**L-Olivosyl-oleandolide (see KEGG C11991) [Macrolides and lactone polyketides [PK04]]**	
**1247**	**0.008038**	**-2.143**	**277.092868**	**278.100145**	**-0.082506**	**5**	**Ph**	**C11H18O8**	**Tuliposide A**	**Isovalerylglucuronide (see KEGG C03033); (2S,3S,4S,5R,6S)-3,4,5-trihydroxy-6-(3-methylbutanoyloxy)oxane-2 carboxylic acid [secondary alcohol]**	
2506	0.010083	-2.134	336.129968	337.137244	-0.110462	3	AmS	C13H23NO9	Streptobiosamine		
5460	0.026091	-2.126	467.119547	468.126824	0.101485	3	Ph	C21H24O12	Catechin-4-ol 3-O-β-D-galactopyranoside [Flavans, Flavanols and Leucoanthocyanidins [PK1202]]	Catechin-4-ol 3-O-β-D-glycopyranoside [Flavans, Flavanols and Leucoanthocyanidins [PK1202]]	Leucodelphinidin 3-O-α-L-rhamnopyranoside [Flavans, Flavanols and Leucoanthocyanidins [PK1202]]
6097	0.002255	-2.126	503.161797	504.169074	0.077257	21	Cb	C18H32O16	Raffinose; Melitose; Melitriose; Gossypose; 6G-α-D-galactosylsucrose	Panose	Galactomannan; Galactomannoglycan
**1303**	**0.013100**	**-2.121**	**281.139425**	**282.146702**	**-0.078970**	**6**	**Ph**	**C15H22O5**	**Hymenoxon**	**Qing Hau Sau; Artemisinin**	**Dihydrophaseic acid; (-)-Dihydrophaseic acid**
3153	0.069033	-2.095	359.134719	360.141996	-0.102120	2	Ph	C16H24O9	7-Deoxyloganate	8-Epideoxyloganic acid	
3540	0.038126	-2.086	373.113993	374.121269	-0.074217	4	Ph	C16H22O10	Secologanate	Swertiamarin	Geniposidic acid
**4590**	**0.022306**	**-2.077**	**419.098382**	**420.105659**	**0.027948**	**7**	**Ph**	**C20H20O10**	**5,3',5'-Trihydroxy-3,6,7,8,4'-pentamethoxyflavone [Flavones and Flavonols [PK1211]]**	**5,7,4'-Trihydroxy-3,6,8,3',5'-pentamethoxyflavone [Flavones and Flavonols [PK1211]]**	**5,7,3'-Trihydroxy-3,6,8,4',5'-pentamethoxyflavone [Flavones and Flavonols [PK1211]]**
**3355**	**0.001644**	**-2.075**	**366.140542**	**367.147819**	**-0.074144**	**3**	**AmS**	**C14H25NO10**	**N-Acetyl-6-O-L-fucosyl-D-glucosamine [aldehyde]**	**2-Acetamido-2-deoxy-6-O-a-L-fucopyranosyl-D-glucose [aldehyde]**	**3-O-fucopyranosyl-2-acetamido-2-deoxyglucopyranose; N-[(2R,3R,4R,5R)-4,5,6-trihydroxy-1-oxo-3-[(2R,3S,4R,5S,6S)-3,4,5-trihydroxy-6-methyl-oxan-2-yl]oxy-hexan-2-yl]acetamide [aldehyde]**
5493	0.050417	-2.050	469.135176	470.142452	0.055729	1	Ph	C21H26O12	Plumieride		
80	0.028026	-2.026	179.056091	180.063368	-0.114407	69	Cb	C6H12O6	D-Glucose; Grape sugar; Dextrose	D-Fructose; Levulose; Fruit sugar; D-arabino-Hexulose	D-Galactose

2222	0.013499	-2.026	325.113981	326.121258	-0.120525	6	Cb	C12H22O10	α-L-Fucosyl-1,2-β-D-galactoside; Glycoprotein α-L-fucosyl-(1,2)-D-galactose; Glycolipid α-L-fucosyl-1,2-β-D-galactose; α-L-Fucosyl-(1->2)-D-galactosyl-R	2-O-α-L-Rhamnopyranosyl-D-glucopyranose; Neohesperidose	Robinobiose
4022	***0.003224***	***-2.017***	***391.128449***	***392.135726***	***0.030165***	***5***	***Ph***	***C20H22O8***	***O18 isotope of Piceid; Polydatin; 3,4,5-Trihydroxystilbene-3-β-monoglucoside***	***O18 isotope of Populin***	***O18 isotope of Apigeniflavan 5-O-xyloside [Flavans, Flavanols and Leucoanthocyanidins [PK1202]]***
3544	0.008113	-2.003	373.150383	374.157659	-0.062393	4	Ph	C17H26O9	Deoxyloganin; 7-Deoxyloganin	8-Epideoxyloganin	Deoxyloganin (see KEGG C06071) [C10 isoprenoids (monoterpenes) [PR0102]]
3927	0.049133	-1.972	387.238811	388.246087	-0.041617	1	Lp	C20H36O7	5,6-Dihydroxyprostaglandin F1a (see KEGG C06475) [secondary alcohol]		
2179	0.008813	-1.911	323.098329	324.105606	-0.127735	2	Cb	C12H20O10	Bis-D-fructose 2',1:2,1'-dianhydride; Difructose anhydride I; Di-D-fructose 1,2'; 2,1' dianhydride	D-Fructofuranose 1,2':2,3'-dianhydride	
1864	0.088433	-1.896	311.098334	312.105610	-0.117249	6	Cb	C11H20O10	Vicianose; O-α-L-Arabinopyranosyl-(1-6)-D-glucopyranose	Arabino-galactose	6-O-β-D-Xylopyranosyl-D-glucose; Primeverose
3972	0.029020	-1.878	389.181684	390.188961	-0.055764	1	Lp	C18H30O9	(-)-11-hydroxy-9,10-dihydrojasmonic acid 11-β-D-glucoside; {(1R,2R)-2-[4-(β-D-glucopyranosyloxy)pentyl]-3-oxocyclopentyl}acetic acid [Jasmonic acids [FA0202]]		
170	0.004956	-1.761	199.170322	200.177598	-0.159280	13	Lp	C12H24O2	Dodecanoic acid; Dodecanoate; Dodecylcarboxylate; Lauric acid	Dodecanoic acid (see KEGG C02679); Dodecanoic acid [carboxylic acid]	Lauric acid (see KEGG C02679); dodecanoic acid [Straight chain fatty acids [FA0101]]

TABLES of COMMON *Ds* and *Np* DOWN-ACCUMULATED COMPOUNDS

Table lists of common annotated down-accumulated significant *m/z* between fungus *Neofusicoccum parvum* and *Diplodia seriata* (*Np* or *Ds*) inoculated-vines and non-inoculated ones under abiotic stresses: none (NAS), water (WS), heat (HS) or both stresses (WSHS). Lists of down-accumulated significant *m/z* in inoculated vines were compared per stress at Time-points A or B. Each compound is identified with an ID number and level of down-regulation is expressed by the difference versus non-infected samples by Perseus software (Student T-test FDR < 0.05, S0=0). Measured *m/z* and calculated KEGG theoretical Mass and error (ppm) are indicated and the number of possible MassTrix identifications is indicated in the "n" column but only the first three ones are listed. Functional categories (Cat.) were determined according to KEGG annotations: Carbohydrates (Cb); Lipids (Lp); Phytochemicals (Ph); Peptides (Pp); Amino sugars (AmS); Nucleotides (Nu) and no match (NM). Down-accumulated (-) compounds are colored in red gradient. Identified isotopic compounds are indicated in italic characters.

h) List of the 15 identified compounds from the 105 common significant *m/z* of no abiotic stress (NAS) samples at time B. Functional category counts: Ph:10; Lp:1; Cb:2; Pp:0; AmS:1; Nu:1; NM:0.

ID	NAS *Ds*- NI	NAS *Np*- NI	Cat.	*m/z*	Mass	error (ppm)	Formula	n	Identification		
7463	-2.686	-2.882	Ph	609.188462	610.195738	0.997565	C34H30N2O9	1	Atalanine		
3591	-2.367	-1.771	Ph	375.129648	376.136925	-0.059642	C16H24O10	3	Loganate; Loganic acid	7-Epiloganic acid	
5540	-2.302	-2.815	Ph	472.146076	473.153353	0.058057	C20H27NO12	1	Proteacin		
3851	-2.301	-1.480	Ph	385.077611	386.084888	-0.061436	C16H18O11	1	O-Feruloylgalactarate; O-Feruloylgalactaric acid; 2'-(E)-O-Feruloylgalactaric acid		
2347	-2.287	-2.287	AmS	330.119400	331.126676	-0.122722	C14H21NO8	1	5'-O-β-D-Glucosylpyridoxine		
3153	-2.279	-2.118	Ph	359.134719	360.141996	-0.102120	C16H24O9	2	7-Deoxyloganate	8-Epideoxyloganic acid	
1590	-2.233	-2.706	Cb	299.113594	300.120870	-0.109047	C14H20O7	1	Salidroside; p-Hydroxyphenethyl alcohol 1-O-β-D-glucoside; Tyrosol glucoside		
2745	-2.156	-1.828	Lp	345.119057	346.126333	-0.142585	C15H22O9	4	Aucubin	Deutzioside	Aucubin (see KEGG C09771) [C10 isoprenoids (monoterpenes) [PR0102]]
3775	-1.871	-1.846	Nu	382.100408	383.107685	-0.072174	C14H17N5O8	1	Succinyladenosine; (2S)-2-[[9-[(2S,3R,4S,5R)-3,4-dihydroxy-5-(hydroxymethyl)oxolan-2-yl]purin-6-yl]amino]butanedioic acid [primary alcohol]		
1864	-1.860	-1.826	Cb	311.098334	312.105610	-0.117249	C11H20O10	6	Vicianose; O-α-L-Arabinopyranosyl-(1-6)-D-glucopyranose	Arabino-galactose	6-O-β-D-Xylopyranosyl-D-glucose; Primeverose
1194	-1.551	-2.456	Ph	274.056804	275.064080	-0.131780	C10H13NO8	1	3-β-D-Glucopyranuronosyloxy-5-methylisoxazole		
3454	-1.415	-1.838	Ph	369.082692	370.089969	-0.075560	C16H18O10	1	Fraxin		
2668	-0.818	-1.101	Ph	343.067029	344.074305	-0.120136	C14H16O10	1	Theogallin		
1219	-0.494	-0.349	Ph	275.092466	276.099742	-0.114667	C15H16O5	5	Lactucin	Vernolepin	Vernomenin
2127	-0.294	-0.433	Ph	321.134320	322.141596	-0.131584	C17H22O6	2	Tetraneurin A	epi-Tulipinolide diepoxide	

i) List of the 106 identified compounds from the 486 common significant *m/z* of HS stress samples at time A. Structure category counts: Ph:56; Lp:13; Cb:28; Pp:2; AmS:3; Nu:0; NM:4

ID	HS *Ds*-NI	HS *Np*-NI	Cat.	*m/z*	Mass	error (ppm)	Formula	n	Identification		
1918	-2.620	-2.307	NM	313.147866	314.155142	0.528679	C19H23ClN2	1	Clomipramine		
1677	-2.589	-2.438	Ph	303.050993	304.058270	-0.108829	C15H12O7	7	Taxifolin; Dihydroquercetin; (+)-Dihydroquercetin; (+)-Taxifolin; *trans*-Dihydroquercetin	Pentahydroxyflavanone; Dihydrotricetin	*cis*-Dihydroquercetin
5431	-2.508	-2.144	Ph	465.103882	466.111159	0.069908	C21H22O12	6	Quercetin 7-O-glucoside	Plantagoside	3,4,5,2',4',6'-Hexahydroxychalcone 2'-glucoside [Chalcones and dihydrochalcones [PK1212]]
1590	-2.490	-2.126	Cb	299.113594	300.120870	-0.109047	C14H20O7	1	Salidroside; p-Hydroxyphenethyl alcohol 1-O-β-D-glucoside; Tyrosol glucoside		

2057	-2.427	-1.793	Ph	319.045905	320.053181	-0.113324	C15H12O8	3	Dihydromyricetin; Ampelopsin; (+)-Dihydromyricetin; (+)-Ampelopsin	2,3-Dihydrogossypetin	Amaronol A; (+)- 2,4,6-Trihydroxy-2-[(3,4,5-trihydroxyphenyl)methyl]-3(2H)-benzofuranone [Aurone flavonoids [PK1213]]
1409	-2.383	-1.745	Ph	289.071731	290.079007	-0.106598	C15H14O6	35	*cis*-3,4-Leucopelargonidin	Luteoforol; 3-Deoxyleucocyanidin	(+)-Catechin; D-Catechin; Cyanidanol; (2R-*trans*)-2-(3,4-dihydroxyphenyl)-3,4-dihydro-2H-1-Benzopyran-3,5,7-triol; (2R,3S)-Catechin; (2R,3S)-(+)-Catechin; Cianidanol
5133	-2.371	-2.027	Ph	449.108952	450.116229	0.038519	C21H22O11	25	Neoastilbin; (2S,3S)-Taxifolin 3-rhamnoside	2',3,4,4',6'-Peptahydroxychalcone 4'-O-glucoside; PHC 4'-O-glucoside	Astilbin
4847	-2.358	-2.075	Ph	433.114041	434.121318	0.048061	C21H22O10	32	Naringenin 7-O-β-D-glucoside; Prunin	Phlorizin chalcone; Chalcone 2'-O-glucoside	2',4,4',6'-Tetrahydroxychalcone 4'-O-glucoside; THC 4'-glucoside
2347	-2.288	-2.354	AmS	330.119400	331.126676	-0.122722	C14H21NO8	1	5'-O-β-D-Glucosylpyridoxine		
2277	-2.285	-2.081	Lp	327.163515	328.170791	-0.119791	C17H28O4S	1	Steryl sulfate		
1158	-2.272	-2.272	Ph	271.061181	272.068458	-0.058348	C15H12O5	37	Naringenin; 4',5,7-Trihydroxyflavanone	(6aS,11aS)-3,6a,9-Trihydroxypterocarpan; 3,6,9-Trihydroxypterocarpan; (-)-Glycinol	Licodione
1346	-2.212	-2.212	Ph	285.040436	286.047712	-0.089823	C15H10O6	34	Luteolin; 3',4',5,7-Tetrahydroxyflavone; 5,7,3',4'-Tetrahydroxyflavone	Kaempferol; 3,4',5,7-Tetrahydroxyflavone; 4H-1-Benzopyran-4-one,3,5,7-trihydroxy-2-(4-hydroxyphenyl)-5,7,4'-Trihydroxyflavonol; C,I, 75640; Indigo yellow; Kaempherol; Kempferol; Nimbecetin; Pelargidenolon; Populnetin; Rhamnolutein; Rhamnolutin; Robigenin; Swartziol; Trifolitin	Aureusidin
7597	-2.207	-2.207	Ph	623.161749	624.169025	-0.015889	C28H32O16	60	Tectorigenin 7-O-gentiobioside [Isoflavonoids [PK1205]]	Isorhamnetin 3-neohesperidoside [Flavones and Flavonols [PK1211]]	Narcissin [Flavones and Flavonols [PK1211]]
3049	-2.183	-2.081	Ph	355.103413	356.110690	-0.119119	C16H20O9	3	Gentiopicrin; Gentiopicroside	1-O-Feruloyl-β-D-glucose; 1-Feruloyl-D-glucose	Gentiopicrin (see KEGG C09782) [C10 isoprenoids (monoterpenes) [PR0102]]
4887	-2.146	-2.028	Ph	435.129686	436.136963	0.036110	C21H24O10	16	Phlorizin; Phlorhizin; Phloridzin	Epiafzelechin 3-O-β-D-allopyranoside [Flavans, Flavanols and Leucoanthocyanidins [PK1202]]	Arthromerin B [Flavans, Flavanols and Leucoanthocyanidins [PK1202]]
1376	-2.130	-1.998	Ph	287.056089	288.063365	-0.079954	C15H12O6	33	Dihydrokaempferol; Aromadendrin; (+)-Dihydrokaempferol; (+)-Aromadendrin	Fustin; 2,3-Dihydrofisetin	Eriodictyol
5691	-2.127	-1.739	Ph	481.098785	482.106061	0.042399	C21H22O13	1	3,4,2',3',4',6',α-Heptahydroxychalcone 2'-		

									glucoside [Chalcones and dihydrochalcones [PK1212]]		
2893	-2.104	-2.104	Lp	350.306446	351.313723	-0.019531	C22H41NO2	1	Anandamide (20:2, n-6); N-(11Z,14Z-eicosadienoyl)-ethanolamine [N-acyl ethanolamines (endocannabinoids) [FA0804]]		
4627	-2.091	-2.091	Ph	421.114055	422.121331	0.081091	C20H22O10	7	Catechin 7-O-β-D-xyloside	Plicatic acid	Catechin 7-O-β-D-xyloside [Flavans, Flavanols and Leucoanthocyanidins [PK1202]]
2745	-2.084	-1.822	Lp	345.119057	346.126333	-0.142585	C15H22O9	4	Aucubin	Deutzioside	Aucubin (see KEGG C09771) [C10 isoprenoids (monoterpenes) [PR0102]]
3088	-2.079	-2.079	Ph	357.061579	358.068856	-0.032332	C18H14O8	10	5,3'-Dihydroxy-4',5'-dimethoxy-6,7-methylenedioxyisoflavone [Isoflavonoids [PK1205]]	Dalpalatin [Isoflavonoids [PK1205]]	Luteolin 7-lactate [Flavones and Flavonols [PK1211]]
5064	-2.048	-2.048	Lp	445.190118	446.197394	-0.100135	C21H34O8S	1	Ptilosteroid A; 5α-pregn-20-en-2β,3β,4β,15β,16β-pentol 2-sulfate [C21 steroids (gluco/mineralocorticoids, progestogins) and derivatives [ST0203]]		
3153	-2.043	-2.052	Ph	359.134719	360.141996	-0.102120	C16H24O9	2	7-Deoxyloganate	8-Epideoxyloganic acid	
2579	-2.037	-2.455	Cb	339.129643	340.136919	-0.081507	C13H24O10	1	Methyl-2-α-L-fucopyranosyl-β-D-galactoside		
3154	-2.025	-1.682	Ph	359.149987	360.157263	-0.070751	C20H24O6	11	Antheridiogen-An	Triptolide	Euponin
3857	-1.998	-1.469	Ph	385.113996	386.121273	-0.063172	C17H22O10	2	1-O-Sinapoyl-β-D-glucose; 1-O-Sinapoyl β-D-glucoside	4-O-β-D-Glucosyl-sinapate	
328	-1.992	-1.482	Ph	227.071321	228.078597	-0.205916	C14H12O3	10	3,4',5-Trihydroxystilbene; Resveratrol	Seselin	Trioxsalen; Trioxysalen; 4,8,5'-Trimethylpsoralen
1255	-1.977	-2.051	Lp	277.217271	278.224548	-0.117541	C18H30O2	62	(6Z,9Z,12Z)-Octadecatrienoic acid; 6,9,12-Octadecatrienoic acid; γ-Linolenic acid; Gamolenic acid	(9Z,12Z,15Z)-Octadecatrienoic acid; α-Linolenic acid; 9,12,15-Octadecatrienoic acid; Linolenate; α-Linolenate	Crepenynate; (9Z)-Octadec-9-en-12-ynoate; (Z)-9-Octadecen-12-ynoic acid; Crepenynic acid
5457	-1.967	-1.760	Ph	467.098394	468.105671	0.051411	C24H20O10	2	Epigallocatechin 3-O-caffeate [Flavans, Flavanols and Leucoanthocyanidins [PK1202]]	Apocynin A [Flavans, Flavanols and Leucoanthocyanidins [PK1202]]	
51	-1.967	-2.107	Ph	173.009145	174.016422	-0.092226	C6H6O6	6	*cis*-Aconitate; *cis*-Aconitic acid	*trans*-Aconitate; *trans*-Aconitic acid	Dehydroascorbate; Dehydroascorbic acid; L-Dehydroascorbate; L-Dehydroascorbic acid
3101	-1.932	-1.938	Cb	357.119069	358.126345	-0.103262	C16H22O9	3	Tarennoside	10-Deoxygeniposidic acid	Sweroside
1247	-1.922	-1.922	Ph	277.092868	278.100145	-0.082506	C11H18O8	5	Tuliposide A	Isovalerylglucuronide (see KEGG C03033); (2S,3S,4S,5R,6S)-3,4,5-trihydroxy-6-(3-methylbutanoyloxy)oxane-2 carboxylic acid [secondary alcohol]	
7603	-1.906	-1.906	Cb	623.255794	624.263070	0.216834	C27H44O16	1	Kanokoside D		

1304	-1.898	-2.205	Lp	281.248574	282.255850	-0.106353	C18H34O2	58	(9Z)-Octadecenoic acid; (Z)-Octadec-9-enoic acid; Oleate; Oleic acid	(9E)-Octadecenoic acid; 9-Octadecenoic acid, (E)-; (E)-Oleic acid; D9-*trans*-Octadecenoic acid; 9-*trans*-Octadecenoic acid; *trans*-D9-Octadecenoic acid; *trans*-9-Octadecenoic acid; *trans*-Elaidic acid; *trans*-Oleic acid; Elaidic acid; Acide elaidique; Elaidinsaure	(6Z)-Octadecenoic acid; Petroselinic acid; *cis*-6-Octadecenoic acid
8583	-1.884	-1.348	Lp	743.486985	744.494261	0.169877	C40H73O10P	18	PG(16:0/18:3(6Z,9Z,12Z)); 1-hexadecanoyl-2-(6Z,9Z,12Z-octadecatrienoyl)-sn-glycero-3-phospho-(1'-sn-glycerol) [primary alcohol]	PG(16:0/18:3(9Z,12Z,15Z)); 1-hexadecanoyl-2-(9Z,12Z,15Z-octadeatrienoyl)-sn-glycero-3-phospho-(1'-sn-glycerol) [primary alcohol]	PG(16:1(9Z)/18:2(9Z,12Z)); 1-(9Z-hexadecenoyl)-2-(9Z,12Z-octadecadienoyl)-sn-glycero-3-phospho-(1'-sn-glycerol) [primary alcohol]
4814	-1.857	-2.258	Cb	431.155905	432.163181	0.044900	C19H28O11	1	Zizybeoside I		
5036	-1.831	-2.007	Cb	443.192275	444.199552	0.009678	C21H32O10	1	dihydrophaseic acid 4-O-β-D-glucoside; (2Z,4E)-5-[(1R,3S,5R,8S)-3-(β-D-glucopyranosyloxy)-8-hydroxy-1,5-dimethyl-6-oxabicyclo[3,2,1]oct-8-yl]-3-methylpenta-2,4-dienoic acid [C15 isoprenoids (sesquiterpenes) [PR0103]]		
4021	-1.828	-1.828	Ph	391.124562	392.131838	-0.059958	C16H24O11	1	Shanzhiside		
449	-1.808	-1.808	Ph	243.066250	244.073527	-0.131492	C14H12O4	10	3,3',4'5-Tetrahydroxystilbene; Piceatannol	Wyerone acid	Eriobofuran
5171	-1.806	-1.648	Ph	451.103475	452.110751	0.041592	C24H20O9	6	Cinchonain 1a	9,10-Dihydro-10-(3,4-dihydroxyphenyl)-pyrano[2,3-h]catechin-8-one [Flavans, Flavanols and Leucoanthocyanidins [PK1202]]	Cinchonain Ib [Flavans, Flavanols and Leucoanthocyanidins [PK1202]]
4599	-1.804	-1.804	Ph	419.171164	420.178440	0.053946	C22H28O8	6	Eupacunolin	Eupacunoxin	Eupaformosanin
4027	*-1.795*	*-2.088*	NM	*391.160971*	*392.168248*	*0.453424*	*C21H27ClN2O3*	*1*	*Cl37 isotope of Compound II(R/S); 5-(7-(6-Chloro-4-(5-hydro-4-methyl-2-oxazolyl)phenoxy)heptyl)-3-methylisoxazole*		
6042	-1.751	-1.751	NM	499.291301	500.298577	0.088261	C26H44O9	1	Mupirocin		
4022	*-1.742*	*-1.742*	Ph	*391.128449*	*392.135726*	*0.030165*	*C20H22O8*	*5*	*O18 isotope of Piceid; Polydatin; 3,4,5-Trihydroxystilbene-3-β-monoglucoside*	*O18 isotope of Populin*	*O18 isotope of Apigeniflavan 5-O-xyloside [Flavans, Flavanols and Leucoanthocyanidins [PK1202]]*
2179	-1.738	-1.506	Cb	323.098329	324.105606	-0.127735	C12H20O10	2	Bis-D-fructose 2',1:2,1'-dianhydride; Difructose anhydride I; Di-D-fructose 1,2'; 2,1' dianhydride	D-Fructofuranose 1,2':2,3'-dianhydride	

4636	-1.720	-1.525	Cb	421.207924	422.215201	0.008100	C19H34O10	1	1-Octen-3-ol-3-o-β-D-xylopyranosyl(1->6)-β-D-glucopyranoside		
6367	-1.714	-1.879	Ph	519.187226	520.194503	0.078545	C26H32O11	2	Brusatol	(-)-Pinoresinol glucoside	
3918	-1.713	-2.081	Cb	387.129645	388.136921	-0.066108	C17H24O10	5	Secologanin; (-)-Secologanin	Geniposide	Verbenalin; Cornin (glycoside)
3969	*-1.713*	*-1.783*	NM	*389.145306*	*390.152582*	*0.415595*	*C21H25ClN2O3*	*4*	*Cl37 isotope of Cetirizine*	*Cl37 isotope of Cetirizine (see KEGG C07778); 2-[2-[4-[(4-chlorophenyl)-phenyl-methyl]piperazin-1-yl]ethoxy]acetic acid [dialkyl ether]*	*Loganin*
11	-1.706	-2.180	Cb	161.045532	162.052809	-0.091525	C6H10O5	30	Lichenin; Lichenan	(R)-3,3-Dimethylmalate	3-Ethylmalate
1843	-1.705	-1.839	Ph	310.093192	311.100468	-0.108516	C14H17NO7	4	Taxiphyllin; (R)-4-Hydroxymandelonitrile β-D-glucoside	Dhurrin; (S)-4-Hydroxymandelonitrile β-D-glucoside	p-Glucosyloxymandelonitrile
2811	-1.696	-1.429	Cb	347.171101	348.178377	-0.116791	C16H28O8	1	Schizonepetoside E		
4393	-1.694	-1.526	Ph	407.171139	408.178415	-0.005805	C21H28O8	2	Vernoflexuoside		
4594	-1.690	-1.690	Ph	419.134784	420.142061	0.067492	C21H24O9	10	Melampodin A	Glycyphyllin; Phloretin 2'-O-rhamnoside	Isorhapontin
2276	-1.688	-1.688	Ph	327.144911	328.152188	-0.047159	C16H24O7	3	Rhododendrin	Tetranor-PGEM; 11R-hydroxy-9,15-dioxo-2,3,4,5-tetranor-prostan-1,20-dioic acid [Prostaglandins [FA0301]]	Prostaglandin M; 7R-hydroxy-5,11-dioxotetranorprostane-1,16-dioic acid [Prostaglandins [FA0301]]
4985	-1.682	-1.799	Ph	441.082837	442.090114	0.264769	C22H18O10	10	Robinetinidol 3-O-gallate [Flavans, Flavanols and Leucoanthocyanidins [PK1202]]	ent-Robinetinidol 3-O-gallate [Flavans, Flavanols and Leucoanthocyanidins [PK1202]]	Catechin 3-O-gallate [Flavans, Flavanols and Leucoanthocyanidins [PK1202]]
4292	-1.672	-1.672	Ph	403.139861	404.147137	0.048967	C21H24O8	4	Mallotophenone	Koaburanin [Flavans, Flavanols and Leucoanthocyanidins [PK1202]]	Orientanol A [Pterocarpans [PK1207]]
4338	-1.666	-1.699	Ph	405.140233	406.147509	-0.005430	C17H26O11	4	Ipolamiide	10-Hydroxyloganin	Morroniside
6989	-1.658	-1.658	Ph	563.140743	564.148019	0.201658	C26H28O14	52	Apigenin 7-O-[β-D-apiosyl-(1->2)-β-D-glucoside]; 7-O-(β-D-Apiofuranosyl-1,2-β-D-glucosyl)-5,7,4'-trihydroxyflavone; Apiin; 7-[(2-O-D-Apio-β-D-furanosyl-β-D-glucopyranosyl)oxy]-5-hydroxy-2-(4-hydroxy-phenyl)-4H-1-benzopyran-4-one; Apioside	Neoschaftoside	Schaftoside
3591	-1.657	-1.724	Ph	375.129648	376.136925	-0.059642	C16H24O10	3	Loganate; Loganic acid	7-Epiloganic acid	
3500	-1.657	-1.646	Ph	371.134722	372.141998	-0.092343	C17H24O9	1	Syringin; Eleutheroside B		
3072	-1.653	-1.633	AmS	356.098668	357.105944	-0.103781	C15H19NO9	1	cyclo-Dopa 5-O-glucoside		
2539	*-1.650*	*-1.757*	Ph	*337.113983*	*338.121259*	*0.392818*	*C12H20N2O9*	*1*	*O18 isotope of 3-Epihydroxymugineic acid*		
4597	-1.648	-1.696	Ph	419.155889	420.163166	0.009385	C18H28O11	2	Lamioside	Lamioside (see KEGG C11645) [C10 isoprenoids (monoterpenes) [PR0102]]	
3972	-1.622	-1.830	Lp	389.181684	390.188961	-0.055764	C18H30O9	1	(-)-11-hydroxy-9,10-dihydrojasmonic acid 11-β-D-		

									glucoside; {(1R,2R)-2-[4-(β-D-glucopyranosyloxy)pentyl]-3-oxocyclopentyl}acetic acid [Jasmonic acids [FA0202]]		
7153	-1.619	-1.227	Ph	577.135234	578.142510	0.145859	C30H26O12	22	Epicatechin-(4β->8)-ent-epicatechin	Procyanidin B4; Catechin-(4α->8)-epicatechin	Procyanidin B2; Procyanidol B2
1733	*-1.603*	*-1.800*	Ph	*305.124160*	*306.131437*	*0.457167*	*C12H20N2O7*	*1*	*O18 isotope of 2'-Deoxymugineic acid*		
3967	-1.597	-1.371	Ph	389.124168	390.131444	-0.060382	C20H22O8	5	Piceid; Polydatin; 3,4,5-Trihydroxystilbene-3-β-monoglucoside	Populin	Apigeniflavan 5-O-xyloside [Flavans, Flavanols and Leucoanthocyanidins [PK1202]]
3651	-1.590	-1.643	Ph	377.160554	378.167830	-0.061522	C20H26O7	5	Chaparrinone	Cnicin	Eupahyssopin
4289	-1.586	-1.765	Ph	403.124570	404.131847	-0.036646	C17H24O11	2	Gardenoside	Scandoside methyl ester	
4511	-1.585	-1.967	Lp	413.218120	414.225397	0.069673	C21H34O8	3	methyl 5-hydroperoxy-6,8,9,11-bisepidioxy-12,14-eicosadienoate [Hydroperoxy fatty acids [FA0104]]	methyl 9,11-epidioxy-12,15-dihydroperoxy-5,7,13-eicosatrienoate [Hydroxy/hydroperoxyeicosatrienoic acids [FA0305]]	methyl 6,8-epidioxy-5,15-dihydroperoxy-9,11,13-eicosatrienoate [Hydroxy/hydroperoxyeicosatrienoic acids [FA0305]]
2807	-1.560	-1.481	Cb	347.134705	348.141981	-0.146980	C15H24O9	1	Leonuridine		
1303	-1.544	-1.835	Ph	281.139425	282.146702	-0.078970	C15H22O5	6	Hymenoxon	Qing Hau Sau; Artemisinin	Dihydrophaseic acid; (-)-Dihydrophaseic acid
4441	-1.541	-1.531	Ph	409.150412	410.157688	0.014166	C20H26O9	2	Bruceine D	C20H26O9	
80	-1.538	-1.984	Cb	179.056091	180.063368	-0.114407	C6H12O6	69	D-Glucose; Grape sugar; Dextrose	D-Fructose; Levulose; Fruit sugar; D-arabino-Hexulose	D-Galactose
1351	-1.532	-1.627	Cb	285.097947	286.105223	-0.104355	C13H18O7	4	Salicin; Salicoside	Gastrodin; 4-(Hydroxymethyl)phenyl-β-D-glucopyranoside	Methylarbutin
3540	-1.529	-1.619	Ph	373.113993	374.121269	-0.074217	C16H22O10	4	Secologanate	Swertiamarin	Geniposidic acid
5778	*-1.528*	*-1.528*	Cb	*485.082492*	*486.089768*	*0.446498*	*C20H20O14*	*1*	*O18 isotope of 1-O,6-O-Digalloyl-β-D-glucose*		
2684	-1.519	-1.787	Cb	343.124543	344.131820	-0.121434	C12H24O11	4	Melibiitol; 6-O-α-D-Galactosyl-D-glucitol	Clusianose	Maltitol; 4-[3,4,5-trihydroxy-6-(hydroxymethyl)oxan-2-yl]oxyhexane-1,2,3,5,6-pentol [acetal]
2222	-1.506	-1.601	Cb	325.113981	326.121258	-0.120525	C12H22O10	6	α-L-Fucosyl-1,2-β-D-galactoside; Glycoprotein α-L-fucosyl-(1,2)-D-galactose; Glycolipid α-L-fucosyl-1,2-β-D-galactose; α-L-Fucosyl-(1->2)-D-galactosyl-R	2-O-α-L-Rhamnopyranosyl-D-glucopyranose; Neohesperidose	Robinobiose
6404	-1.504	-1.603	Ph	521.202869	522.210145	0.064409	C26H34O11	2	Isobrucein A	Quassimarin	
3922	-1.497	-2.042	Lp	387.166034	388.173310	-0.057526	C18H28O9	2	Tuberonic acid glucoside	Tuberonic acid glucoside (see KEGG C08558); (1R,2S)-3-oxo-2-(5'-O-β-D-glucopyranosyloxy-2'Z-pentenyl)-cyclopentaneacetic acid [Fatty acyl glycosides of mono- and disaccharides [FA1301]]	

199	-1.489	-1.629	Ph	205.035328	206.042604	-0.236504	C7H10O7	7	(R)-2-Hydroxybutane-1,2,4-tricarboxylate; Homocitrate; Homocitric acid; 3-Hydroxy-3-carboxyadipic acid; (R)-2-Hydroxy-1,2,4-butanetricarboxylic acid	2-Methylcitrate; 2-Hydroxybutane-1,2,3-tricarboxylate; (2R,3S)-2-Hydroxybutane-1,2,3-tricarboxylate	(2S,3R)-3-Hydroxybutane-1,2,3-tricarboxylate; Methylisocitrate; Methylisocitric acid
2088	-1.470	-1.476	Pp	320.062277	321.069553	-0.132751	C11H15NO10	1	β-Cityl-L-glutamic acid; 3-[[(2S)-1,5-dihydroxy-1, 5-dioxopentan-2-yl]carbamoyl]-3-hydroxypentanedioic acid [tertiary alcohol]		
3977	-1.467	-1.400	Cb	389.218068	390.225344	-0.061128	C19H34O8	2	Rehmaionoside A	Rehmaionoside B	
3598	-1.437	-1.789	Cb	375.202440	376.209716	-0.005151	C18H32O8	1	2,3-di-0-hexanoyl-α-glucopyranose [Other acyl sugars [SL05]]		
3508	-1.433	-1.751	Lp	371.243903	372.251179	-0.025932	C20H36O6	5	8,8a-Deoxyoleandolide	19(R)-hydroxy-PGF1α; 9S,11R,15S,19R-tetrahydroxy-13E-prostaenoic acid [Prostaglandins [FA0301]]	13,14-dihydro-19(R)-hydroxyPGE1; 9-oxo-11R,15S,19R-trihydroxy-prostanoic acid [Prostaglandins [FA0301]]
2074	-1.420	-1.372	Cb	319.139804	320.147080	-0.116812	C14H24O8	2	Valproic acid glucuronide (see KEGG C03033); 3,4,5-trihydroxy-6-(2-propylpentanoyloxy)oxane-2-carboxylic acid [secondary alcohol]	Octanoylglucuronide (see KEGG C03033) [secondary alcohol]	
6097	-1.407	-1.308	Cb	503.161797	504.169074	0.077257	C18H32O16	21	Raffinose; Melitose; Melitriose; Gossypose; 6G-α-D-galactosylsucrose	Panose	Galactomannan; Galactomannoglycan
7699	-1.389	-1.146	Ph	633.073451	634.080728	0.180016	C27H22O18	1	Corilagin		
574	-1.380	-1.420	Lp	255.232918	256.240194	-0.141782	C16H32O2	23	Hexadecanoic acid; Hexadecanoate; Hexadecylic acid; Palmitic acid; Palmitate; Cetylic acid	Palmitic acid (see KEGG C00249); Hexadecanoic acid [carboxylic acid]	Trimethyltridecanoic acid; 4,8,12-trimethyltridecanoic acid [carboxylic acid]
1730	*-1.378*	*-1.467*	Pp	*305.087764*	*306.095041*	*0.422086*	*C11H16N2O8*	*2*	*O18 isotope of N-Acetyl-aspartyl-glutamate; NAAG*	*O18 isotope of N-Acetylaspartylglutamic acid (see KEGG C12270); 2-[(2-acetamido-3-carboxy-propanoyl)amino]pentanedioic acid [carboxylic acid]*	
1586	-1.363	-1.218	Ph	299.077209	300.084485	-0.108396	C13H16O8	1	4-(β-D-Glucosyloxy)benzoate		
4335	-1.358	-1.064	Ph	405.119098	406.126375	-0.018591	C20H22O9	7	Astringin	Trichocarpin	Anadanthoside [Flavans, Flavanols and Leucoanthocyanidins [PK1202]]
7306	-1.352	-1.032	Cb	593.130164	594.137440	0.167509	C30H26O13	16	Tribuloside	Apigenin 7-glucoside-4'-*trans*-caffeate [Flavones and Flavonols [PK1211]]	Apigenin 7-(6"-E-Caffeoylglucoside) [Flavones and Flavonols [PK1211]]
3145	-1.292	-1.382	Cb	359.098337	360.105614	-0.091802	C15H20O10	1	3-Methoxy-4-hydroxyphenylglycol glucuronide (see KEGG C03033);		

									(2S,3S,4S,5R,6R)-3,4,5-trihydroxy-6-[2-hydroxy-2-(4-hydroxy-3-methoxy-phenyl)ethoxy]oxane-2-carboxylic acid [acetal]		
2575	-1.287	-1.307	Cb	339.093245	340.100522	-0.117669	C12H20O11	4	Cellobiono-1,5-lactone; Cellobiose-1,5-lactone	3-Ketolactose	3-Ketosucrose; 3-Dehydro-α-D-glucosyl-β-D-fructofuranoside
291	-1.279	-1.736	Cb	221.066628	222.073904	-0.220142	C8H14O7	4	6-Acetyl-D-glucose	6-Acetyl-β-D-galactoside	Ethyl glucuronide; (2S,3S,4S,5R)-6-ethoxy-3,4,5-trihydroxyoxane-2-carboxylic acid [acetal]
5262	-1.261	-1.114	Ph	455.353108	456.360385	0.086530	C30H48O3	20	Betulinic acid	α-Elemolic acid	Ursolic acid
4329	-1.226	-1.712	Ph	405.082709	406.089985	-0.028041	C19H18O10	7	Lancerin; 4-C-Glucosyl-1,3,7-trihydroxyxanthone	5,7,3',5'-Tetrahydroxy-3,6,8,4'-tetramethoxyflavone [Flavones and Flavonols [PK1211]]	5,7,3',4'-Tetrahydroxy-3,6,8,5'-tetramethoxyflavone [Flavones and Flavonols [PK1211]]
5658	-1.183	-1.001	Cb	479.083140	480.090416	0.053553	C21H20O13	18	Gossypin; Gossypetin 8-O-glucoside	4-o-Galloylbergenin	11-o-Galloylbergenin
224	-1.134	-1.746	Cb	209.066629	210.073905	-0.227478	C7H14O7	5	Sedoheptulose; D-altro-Heptulose; D-Sedoheptulose; Volemulose	α-D-Mannoheptulopyranose; Mannoheptulose	β-D-Sedoheptulopyranose; β-D-Sedoheptulose
2506	-1.060	-2.069	AmS	336.129968	337.137244	-0.110462	C13H23NO9	3	Streptobiosamine		
8856	-1.010	-0.816	Lp	831.502796	832.510072	-0.128394	C43H77O13P	12	PI(14:0/20:3(8Z,11Z,14Z)); 1-tetradecanoyl-2-(8Z,11Z,14Z-eicosatrienoyl)-glycero-3-phospho-(1'-myo-inositol) [Diacylglycerophosphoinositols [GP0601]]	PI(14:1(9Z)/20:2(11Z,14Z)); 1-(9Z-tetradecenoyl)-2-(11Z,14Z-eicosadienoyl)-glycero-3-phospho-(1'-myo-inositol) [Diacylglycerophosphoinositols [GP0601]]	PI(17:1(9Z)/17:2(9Z,12Z)); 1-(9Z-heptadecenoyl)-2-(9Z,12Z-heptadecadienoyl)-glycero-3-phospho-(1'-myo-inositol) [Diacylglycerophosphoinositols [GP0601]]
6992	-0.942	-0.942	Ph	563.177052	564.184328	0.065793	C27H32O13	6	Pinocembrin 7-rhamnosylglucoside	Cascaroside D	Cascaroside C
6072	-0.779	-0.905	Cb	501.146171	502.153448	0.125686	C18H30O16	1	α-L-Rhamnopyranosyl-(1->2)-β-D-galactopyranosyl-(1->2)-β-D-glucuronopyranoside; β-Fabatriose		

j) List of the 165 identified compounds from the 761 common significant *m/z* of combined WSHS stresses samples at time A. Functional category counts: Ph:87; Lp:23; Cb:35; Pp:6; AmS:4; Nu:0; NM:10.

ID	WSHS *Ds*-NI	WSHS *Np*-NI	Cat.	*m/z*	Mass	error (ppm)	Formula	n	Identification		
5781	-2.818	-2.818	Ph	485.108961	486.116238	0.054370	C24H22O11	2	Flavonol 3-O-(6-O-malonyl-β-D-glucoside)	Epigallocatechin 3-O-(3,5-di-O-methylgallate) [Flavans, Flavanols and Leucoanthocyanidins [PK1202]]	
1087	*-2.766*	*-2.766*	Pp	*265.144542*	*266.151818*	*0.677275*	*C14H20N2O3*	*6*	*O18 isotope of Subaphyllin; Feruloylputrescine*	*Mukaadial*	*Rugosal*

5566	-2.662	-2.298	Ph	473.363676	474.370953	0.090535	C30H50O4	15	Sapelin A	Bryodulcosigenin	Soyasapogenol A
4717	-2.655	-2.791	Ph	425.087827	426.095103	0.049636	C22H18O9	2	Epiafzelechin 3-O-gallate [Flavans, Flavanols and Leucoanthocyanidins [PK1202]]	Epigallocatechin 3-O-(4-hydroxybenzoate) [Flavans, Flavanols and Leucoanthocyanidins [PK1202]]	
1130	-2.584	-2.584	Ph	269.045564	270.052841	0.064429	C15H10O5	42	Apigenin; 4',5,7-Trihydroxyflavone; 5,7,4'-Trihydroxyflavone	2'-Hydroxydaidzein	Genistein; 5,7,4'-Trihydroxyisoflavone
7699	-2.575	-2.997	Ph	633.073451	634.080728	0.180016	C27H22O18	1	Corilagin		
5278	-2.563	-2.563	Ph	457.077691	458.084967	0.122575	C22H18O11	7	Epigallocatechin 3-gallate	8-Demethyltetracenomycin C	Epigallocatechin gallate (see KEGG C09731); [(2R,3R)-5,7-dihydroxy-2-(3,4,5-trihydroxyphenyl)chroman-3-yl] 3,4,5-trihydroxybenzoate [phenol or hydroxyhetarene]
233	-2.480	-2.480	Lp	211.133944	212.141220	-0.115861	C12H20O3	15	Cucurbic acid	Putaminoxin	Traumatin; (10E)-12-Oxododecenoic acid
5457	-2.465	-2.645	Ph	467.098394	468.105671	0.051411	C24H20O10	2	Epigallocatechin 3-O-caffeate [Flavans, Flavanols and Leucoanthocyanidins [PK1202]]	Apocynin A [Flavans, Flavanols and Leucoanthocyanidins [PK1202]]	
3970	-2.462	-2.462	Ph	389.160552	390.167829	-0.062778	C21H26O7	2	Erioflorin acetate		
5778	*-2.458*	*-2.458*	Cb	*485.082492*	*486.089768*	*0.446498*	*C20H20O14*	*1*	*O18 isotope of 1-O,6-O-Digalloyl-β-D-glucose*		
132	-2.410	-2.410	Ph	191.034952	192.042228	-0.159394	C10H8O4	10	Scopoletin	Naphthazarin; 5,8-Dihydroxy-1,4-naphthoquinone	3,4-Dehydro-6-hydroxymellein
5733	-2.375	-2.375	Ph	483.093470	484.100746	0.382970	C24H20O11	2	Tetracenomycin X		
3099	*-2.369*	*-2.369*	Ph	*357.107697*	*358.114974*	*-0.013607*	*C16H20O9*	*4*	*O18 isotope of Gentiopicrin; Gentiopicroside*	*O18 isotope of 1-O-Feruloyl-β-D-glucose; 1-Feruloyl-D-glucose*	*O18 isotope of Gentiopicrin (see KEGG C09782) [C10 isoprenoids (monoterpenes) [PR0102]]*
3831	-2.353	-2.353	Ph	384.145333	385.152609	0.187448	C21H23NO6	1	Polycarpine		
6042	-2.337	-2.337	NM	499.291301	500.298577	0.088261	C26H44O9	1	Mupirocin		
1843	-2.335	-2.474	Ph	310.093192	311.100468	-0.108516	C14H17NO7	4	Taxiphyllin; (R)-4-Hydroxymandelonitrile β-D-glucoside	Dhurrin; (S)-4-Hydroxymandelonitrile β-D-glucoside	p-Glucosyloxymandelonitrile
4635	-2.320	-2.320	Ph	421.186827	422.194104	0.084740	C22H30O8	4	Valtratum; Valtrate	Isovaltrate	Picrasin F
3088	-2.319	-2.319	Ph	357.061579	358.068856	-0.032332	C18H14O8	10	5,3'-Dihydroxy-4',5'-dimethoxy-6,7-methylenedioxyisoflavone [Isoflavonoids [PK1205]]	Dalpalatin [Isoflavonoids [PK1205]]	Luteolin 7-lactate [Flavones and Flavonols [PK1211]]
5262	-2.317	-2.187	Ph	455.353108	456.360385	0.086530	C30H48O3	20	Betulinic acid	α-Elemolic acid	Ursolic acid
4511	-2.304	-1.932	Lp	413.218120	414.225397	0.069673	C21H34O8	3	methyl 5-hydroperoxy-6,8,9,11-bisepidioxy-12,14-eicosadienoate [Hydroperoxy fatty acids [FA0104]]	methyl 9,11-epidioxy-12,15-dihydroperoxy-5,7,13-eicosatrienoate [Hydroxy/hydroperoxyeicosatrienoic acids [FA0305]]	methyl 6,8-epidioxy-5,15-dihydroperoxy-9,11,13-eicosatrienoate [Hydroxy/hydroperoxyeicosatrienoic acids [FA0305]]
4985	-2.295	-2.728	Ph	441.082837	442.090114	0.264769	C22H18O10	10	Robinetinidol 3-O-gallate [Flavans, Flavanols and Leucoanthocyanidins [PK1202]]	ent-Robinetinidol 3-O-gallate [Flavans, Flavanols and Leucoanthocyanidins [PK1202]]	Catechin 3-O-gallate [Flavans, Flavanols and Leucoanthocyanidins [PK1202]]
4377	-2.291	-2.291	Ph	407.077314	408.084590	0.179320	C22H16O8	4	Tetracenomycin E	Tetracenomycin B2	Torosaflavone C [Flavones and Flavonols [PK1211]]

2893	-2.258	-2.258	Lp	350.306446	351.313723	-0.019531	$C_{22}H_{41}NO_2$	11	Anandamide (20:2, n-6); N-(11Z,14Z-eicosadienoyl)-ethanolamine [N-acyl ethanolamines (endocannabinoids) [FA0804]]		
3591	-2.248	-2.415	Ph	375.129648	376.136925	-0.059642	$C_{16}H_{24}O_{10}$	3	Loganate; Loganic acid	7-Epiloganic acid	
4688	-2.246	-2.246	Lp	423.326893	424.334170	0.092124	$C_{29}H_{44}O_2$	4	α-Tocotrienol	4,4'-Methylenebis(2,6-di-tert-butylphenol)	α-Tocotrienol (see KEGG C14153); (2R)-2,5,7,8-tetramethyl-2-[(3E,7E)-4,8,12-trimethyltrideca-3,7,11-trienyl]chroman-6-ol [phenol or hydroxyhetarene]
4730	-2.237	-2.237	Ph	425.181737	426.189014	0.073384	$C_{21}H_{30}O_9$	2	Abscisic acid glucose ester; abscisic acid-β-D-glucopyranosyl ester	γ-CEHC Glc; γ-carboxyethyl-hydroxychroman β-D-glucoside [Vitamin E [PR0202]]	
2684	-2.216	-2.383	Cb	343.124543	344.131820	-0.121434	$C_{12}H_{24}O_{11}$	4	Melibiitol; 6-O-α-D-Galactosyl-D-glucitol	Clusianose	Maltitol; 4-[3,4,5-trihydroxy-6-(hydroxymethyl)oxan-2-yl]oxyhexane-1,2,3,5,6-pentol [acetal]
5171	-2.211	-2.372	Ph	451.103475	452.110751	0.041592	$C_{24}H_{20}O_9$	6	Cinchonain 1a	9,10-Dihydro-10-(3,4-dihydroxyphenyl)-pyrano[2,3-h]catechin-8-one [Flavans, Flavanols and Leucoanthocyanidins [PK1202]]	Cinchonain Ib [Flavans, Flavanols and Leucoanthocyanidins [PK1202]]
4599	-2.210	-2.210	Ph	419.171164	420.178440	0.053946	$C_{22}H_{28}O_8$	6	Eupacunolin	Eupacunoxin	Eupaformosanin
4636	-2.209	-2.114	Cb	421.207924	422.215201	0.008100	$C_{19}H_{34}O_{10}$	1	1-Octen-3-ol-3-o-β-D-xylopyranosyl(1->6)-β-D-glucopyranoside		
306	-2.204	-2.204	Ph	223.133928	224.141204	-0.180408	$C_{13}H_{20}O_3$	8	Vomifoliol; (6S,9R)-6-hydroxy-3-oxo-α-ionol	(+/-)-6-Hydroxy-3-oxo-α-ionol	Methyl jasmonate; (-)-Methyl jasmonate
1722	-2.181	-1.809	Ph	305.066640	306.073916	-0.120233	$C_{15}H_{14}O_7$	11	Leucocyanidin; 2-(3,4-Dihydroxyphenyl)-3,4-dihydro-2H-1-benzopyran-3,4,5,7-tetrol; 3,3',4,4',5,7-Flavanhexol; 3,4-Cyanidiol; Leucoanthocyanidol; Leucocianidol; Leucocyanidol; Leukocyanidine; Procyanidol; Resivit; 2,3-*trans*-3,4-*cis*-Leucocyanidin	(+)-Gallocatechin; Gallocatechol	(-)-Epigallocatechin
1583	-2.176	-2.176	Ph	299.056112	300.063389	0.002400	$C_{16}H_{12}O_6$	77	5,7,4'-Trihydroxy-3'-methoxyflavone; 3'-O-Methylluteolin; Chrysoeriol	3-Methoxyapigenin; 3-O-Methylkaempferol	Leptosidin
3926	-2.176	-2.584	Cb	387.202414	388.209691	-0.070880	$C_{19}H_{32}O_8$	5	Rehmaionoside C	methyl 9-hydroperoxy-10,12,13,15-bisepidioxy-16E-octadecenoate [Hydroperoxy fatty acids [FA0104]]	methyl 10,12,13,15-bisepidioxy-16-hydroperoxy-8E-octadecenoate [Hydroperoxy fatty acids [FA0104]]
6299	-2.172	-2.172	Lp	515.286219	516.293495	0.092665	$C_{26}H_{44}O_{10}$	2	L-Olivosyl-oleandolide	L-Olivosyl-oleandolide (see KEGG C11991) [Macrolides and lactone polyketides [PK04]]	

1247	-2.143	-2.143	Ph	277.092868	278.100145	-0.082506	C11H18O8	5	Tuliposide A	Isovalerylglucuronide (see KEGG C03033); (2S,3S,4S,5R,6S)-3,4,5-trihydroxy-6-(3-methylbutanoyloxy)oxane-2 carboxylic acid [secondary alcohol]	
2506	-2.134	-2.071	AmS	336.129968	337.137244	-0.110462	C13H23NO9	3	Streptobiosamine		
5460	-2.126	-2.126	Ph	467.119547	468.126824	0.101485	C21H24O12	3	Catechin-4-ol 3-O-β-D-galactopyranoside [Flavans, Flavanols and Leucoanthocyanidins [PK1202]]	Catechin-4-ol 3-O-β-D-glycopyranoside [Flavans, Flavanols and Leucoanthocyanidins [PK1202]]	Leucodelphinidin 3-O-α-L-rhamnopyranoside [Flavans, Flavanols and Leucoanthocyanidins [PK1202]]
6097	-2.126	-2.102	Cb	503.161797	504.169074	0.077257	C18H32O16	21	Raffinose; Melitose; Melitriose; Gossypose; 6G-α-D-galactosylsucrose	Panose	Galactomannan; Galactomannoglycan
1303	-2.121	-2.121	Ph	281.139425	282.146702	-0.078970	C15H22O5	6	Hymenoxon	Qing Hau Sau; Artemisinin	Dihydrophaseic acid; (-)-Dihydrophaseic acid
4338	-2.116	-2.362	Ph	405.140233	406.147509	-0.005430	C17H26O11	4	Ipolamiide	10-Hydroxyloganin	Morroniside
2347	-2.105	-2.304	AmS	330.119400	331.126676	-0.122722	C14H21NO8	1	5'-O-β-D-Glucosylpyridoxine		
4393	-2.102	-2.795	Ph	407.171139	408.178415	-0.005805	C21H28O8	2	Vernoflexuoside		
3153	-2.095	-2.156	Ph	359.134719	360.141996	-0.102120	C16H24O9	2	7-Deoxyloganate	8-Epideoxyloganic acid	
3540	-2.086	-2.238	Ph	373.113993	374.121269	-0.074217	C16H22O10	4	Secologanate	Swertiamarin	Geniposidic acid
4590	-2.077	-1.202	Ph	419.098382	420.105659	0.027948	C20H20O10	7	5,3',5'-Trihydroxy-3,6,7,8,4'-pentamethoxyflavone [Flavones and Flavonols [PK1211]]	5,7,4'-Trihydroxy-3,6,8,3',5'-pentamethoxyflavone [Flavones and Flavonols [PK1211]]	5,7,3'-Trihydroxy-3,6,8,4',5'-pentamethoxyflavone [Flavones and Flavonols [PK1211]]
3355	-2.075	-1.508	AmS	366.140542	367.147819	-0.074144	C14H25NO10	3	N-Acetyl-6-O-L-fucosyl-D-glucosamine [aldehyde]	2-Acetamido-2-deoxy-6-O-a-L-fucopyranosyl-D-glucose [aldehyde]	3-O-fucopyranosyl-2-acetamido-2-deoxyglucopyranose; N-[(2R,3R,4R,5R)-4,5,6-trihydroxy-1-oxo-3-[(2R,3S,4R,5S,6S)-3,4,5-trihydroxy-6-methyl-oxan-2-yl]oxy-hexan-2-yl]acetamide [aldehyde]
1158	-2.056	-2.056	Ph	271.061181	272.068458	-0.058348	C15H12O5	37	Naringenin; 4',5,7-Trihydroxyflavanone	(6aS,11aS)-3,6a,9-Trihydroxypterocarpan; 3,6,9-Trihydroxypterocarpan; (-)-Glycinol	Licodione
5493	-2.050	-1.744	Ph	469.135176	470.142452	0.055729	C21H26O12	(1	Plumieride		
1376	-2.043	-2.159	Ph	287.056089	288.063365	-0.079954	C15H12O6	33	Dihydrokaempferol; Aromadendrin; (+)-Dihydrokaempferol; (+)-Aromadendrin	Fustin; 2,3-Dihydrofisetin	Eriodictyol
80	-2.026	-2.246	Cb	179.056091	180.063368	-0.114407	C6H12O6	69	D-Glucose; Grape sugar; Dextrose	D-Fructose; Levulose; Fruit sugar; D-arabino-Hexulose	D-Galactose
2222	-2.026	-2.601	Cb	325.113981	326.121258	-0.120525	C12H22O10	6	α-L-Fucosyl-1,2-β-D-galactoside; Glycoprotein α-L-fucosyl-(1,2)-D-galactose; Glycolipid α-L-fucosyl-1,2-β-D-galactose; α-L-Fucosyl-(1->2)-D-galactosyl-R	2-O-α-L-Rhamnopyranosyl-D-glucopyranose; Neohesperidose	Robinobiose
3977	-2.025	-2.552	Cb	389.218068	390.225344	-0.061128	C19H34O8	2	Rehmaionoside A	Rehmaionoside B	

4022	*-2.017*	*-2.017*	Ph	*391.128449*	*392.135726*	*0.030165*	*C20H22O8*	*5*	*O18 isotope of Piceid; Polydatin; 3,4,5-Trihydroxystilbene-3-β-monoglucoside*	*O18 isotope of Populin*	*O18 isotope of Apigeniflavan 5-O-xyloside [Flavans, Flavanols and Leucoanthocyanidins [PK1202]]*
328	-2.015	-1.457	Ph	227.071321	228.078597	-0.205916	C14H12O3	10	3,4',5-Trihydroxystilbene; Resveratrol	Seselin	Trioxsalen; Trioxysalen; 4,8,5'-Trimethylpsoralen
3544	-2.003	-2.128	Ph	373.150383	374.157659	-0.062393	C17H26O9	4	Deoxyloganin; 7-Deoxyloganin	8-Epideoxyloganin	Deoxyloganin (see KEGG C06071) [C10 isoprenoids (monoterpenes) [PR0102]]
1409	-1.978	-1.666	Ph	289.071731	290.079007	-0.106598	C15H14O6	35	*cis*-3,4-Leucopelargonidin	Luteoforol; 3-Deoxyleucocyanidin	(+)-Catechin; D-Catechin; Cyanidanol; (2R-*trans*)-2-(3,4-dihydroxyphenyl)-3,4-dihydro-2H-1-Benzopyran-3,5,7-triol; (2R,3S)-Catechin; (2R,3S)-(+)-Catechin; Cianidanol
3927	-1.972	-1.972	Lp	387.238811	388.246087	-0.041617	C20H36O7	1	5,6-Dihydroxyprostaglandin F1a (see KEGG C06475) [secondary alcohol]		
4441	-1.971	-2.412	Ph	409.150412	410.157688	0.014166	C20H26O9	2	Bruceine D		
3594	*-1.966*	*-2.088*	NM	*375.166033*	*376.173310*	*0.410054*	*C21H27ClN2O2*	*1*	*Cl37 isotope of Hydroxyzine*		
1677	-1.963	-2.020	Ph	303.050993	304.058270	-0.108829	C15H12O7	7)	Taxifolin; Dihydroquercetin; (+)-Dihydroquercetin; (+)-Taxifolin; *trans*-Dihydroquercetin	Pentahydroxyflavanone; Dihydrotricetin	*cis*-Dihydroquercetin
7306	-1.938	-1.894	Cb	593.130164	594.137440	0.167509	C30H26O13	16	Tribuloside	Apigenin 7-glucoside-4'-*trans*-caffeate [Flavones and Flavonols [PK1211]]	Apigenin 7-(6"-E-Caffeoylglucoside) [Flavones and Flavonols [PK1211]]
4779	-1.915	-1.915	Ph	429.082814	430.090090	0.218359	C21H18O10	4	Coumestrol 3-O-glucoside [Coumestan flavonoids [PK1209]]	Chrysin 7-glucuronide [Flavones and Flavonols [PK1211]]	Derhamnosylmaysin [Flavones and Flavonols [PK1211]]
2179	-1.911	-1.962	Cb	323.098329	324.105606	-0.127735	C12H20O10	2	Bis-D-fructose 2',1:2,1'-dianhydride; Difructose anhydride I; Di-D-fructose 1,2'; 2,1' dianhydride	D-Fructofuranose 1,2':2,3'-dianhydride	
4289	-1.902	-2.539	Ph	403.124570	404.131847	-0.036646	C17H24O11	2	Gardenoside	Scandoside methyl ester	
1864	-1.896	-2.139	Cb	311.098334	312.105610	-0.117249	C11H20O10	6	Vicianose; O-α-L-Arabinopyranosyl-(1-6)-D-glucopyranose	Arabino-galactose	6-O-β-D-Xylopyranosyl-D-glucose; Primeverose
3972	-1.878	-2.124	Lp	389.181684	390.188961	-0.055764	C18H30O9	1	(-)-11-hydroxy-9,10-dihydrojasmonic acid 11-β-D-glucoside; {(1R,2R)-2-[4-(β-D-glucopyranosyloxy)pentyl]-3-oxocyclopentyl}acetic acid [Jasmonic acids [FA0202]]		
191	-1.857	-2.203	Pp	203.082553	204.089830	-0.236798	C11H12N2O2	10	L-Tryptophan; Tryptophan; (S)-α-Amino-β-(3-indolyl)-propionic acid	D-Tryptophan	Tryptophan; α-Amino-β-(3-indolyl)-propionic acid
8565	-1.851	-1.473	Lp	741.471328	742.478604	0.160654	C40H71O10P	18	PG(16:1(9Z)/18:3(6Z,9Z,12Z)); 1-(9Z-hexadecenoyl)-2-(6Z,9Z,12Z-octadecatrienoyl)-sn-	PG(16:1(9Z)/18:3(9Z,12Z,15Z)); 1-(9Z-hexadecenoyl)-2-(9Z,12Z,15Z-octadeatrienoyl)-sn-	PG(18:3(6Z,9Z,12Z)/16:1(9Z)); 1-(6Z,9Z,12Z-octadecatrienoyl)-2-(9Z-hexadecenoyl)-sn-glycero-

									glycero-3-phospho-(1'-sn-glycerol) [primary alcohol]	glycero-3-phospho-(1'-sn-glycerol) [primary alcohol]	3-phospho-(1'-sn-glycerol) [primary alcohol]
7153	-1.835	-1.624	Ph	577.135234	578.142510	0.145859	C30H26O12	22	Epicatechin-(4β->8)-ent-epicatechin	Procyanidin B4; Catechin-(4α->8)-epicatechin	Procyanidin B2; Procyanidol B2
5489	-1.833	-2.011	Ph	469.114066	470.121342	0.096923	C24H22O10	2	Pongamoside B [Flavones and Flavonols [PK1211]]	Pongamoside C [Flavones and Flavonols [PK1211]]	
5001	-1.830	-2.096	Cb	441.176638	442.183914	0.038479	C21H30O10	1	Lusitanicoside		
2057	-1.817	-1.992	Ph	319.045905	320.053181	-0.113324	C15H12O8	3	Dihydromyricetin; Ampelopsin; (+)-Dihydromyricetin; (+)-Ampelopsin	2,3-Dihydrogossypetin	Amaronol A; (+)- 2,4,6-Trihydroxy-2-[(3,4,5-trihydroxyphenyl)methyl]-3(2H)-benzofuranone [Aurone flavonoids [PK1213]]
6216	-1.816	-2.313	Ph	511.218562	512.225838	0.149097	C25H36O11	1	gibberellin A2 O-β-D-glucoside [C20 isoprenoids (diterpenes) [PR0104]]		
1135	-1.814	-2.546	Lp	269.081904	270.089181	-0.104475	C16H14O4	35	(+)-Medicarpin; 3-Hydroxy-9-methoxypterocarpan	Vignafuran	Alloimperatorin
1730	*-1.813*	*-1.593*	Pp	*305.087764*	*306.095041*	*0.422086*	*C11H16N2O8*	2	*O18 isotope of N-Acetyl-aspartyl-glutamate; NAAG*	*O18 isotope of N-Acetylaspartylglutamic acid (see KEGG C12270); 2-[(2-acetamido-3-carboxy-propanoyl)amino]pentanedioic acid [carboxylic acid]*	
3969	*-1.809*	*-2.378*	NM	*389.145306*	*390.152582*	*0.415595*	*C21H25ClN2O3*	4	*Cl37 isotope of Cetirizine*	*Cl37 isotope of Cetirizine (see KEGG C07778); 2-[2-[4-[(4-chlorophenyl)-phenyl-methyl]piperazin-1-yl]ethoxy]acetic acid [dialkyl ether]*	*Loganin*
4794	-1.809	-1.706	Lp	429.373813	430.381089	0.020016	C29H50O2	11	α-Tocopherol; Vitamin E	4β-Hydroxymethyl-4α-methyl-5α-cholest-7-en-3β-ol	α-Tocopherol (see KEGG C02477); (2R)-2,5,7,8-tetramethyl-2-[(4R,8R)-4,8,12-trimethyltridecyl]chroman-6-ol [phenol or hydroxyhetarene]
8383	-1.801	-1.703	Lp	712.492329	713.499605	0.070622	C39H72NO8P	30	PE(14:0/20:3(5Z,8Z,11Z)) (see KEGG C00350); 1-tetradecanoyl-2-(5Z,8Z,11Z-eicosatrienoyl)-sn-glycero-3-phosphoethanolamine [primary amine]	PE(14:0/20:3(8Z,11Z,14Z)) (see KEGG C00350); 1-tetradecanoyl-2-(8Z,11Z,14Z-eicosatrienoyl)-sn-glycero-3-phosphoethanolamine [primary amine]	PE(14:1(9Z)/20:2(11Z,14Z)) (see KEGG C00350); 1-(9Z-tetradecenoyl)-2-(11Z,14Z-eicosadienoyl)-sn-glycero-3-phosphoethanolamine [primary amine]
291	-1.778	-2.619	Cb	221.066628	222.073904	-0.220142	C8H14O7	4	6-Acetyl-D-glucose	6-Acetyl-β-D-galactoside	Ethyl glucuronide; (2S,3S,4S,5R)-6-ethoxy-3,4,5-trihydroxyoxane-2-carboxylic acid [acetal]
2331	-1.778	-1.733	Ph	329.139407	330.146683	-0.123563	C19H22O5	7	Podolide	Gibberellin A51-catabolite	Gibberellin A7
449	-1.767	-2.125	Ph	243.066250	244.073527	-0.131492	C14H12O4	10	3,3',4'5-Tetrahydroxystilbene; Piceatannol	Wyerone acid	Eriobofuran
170	-1.761	-1.761	Lp	199.170322	200.177598	-0.159280	C12H24O2	13	Dodecanoic acid; Dodecanoate; Dodecylcarboxylate; Lauric acid	Dodecanoic acid (see KEGG C02679); Dodecanoic acid [carboxylic acid]	Lauric acid (see KEGG C02679); dodecanoic acid [Straight chain fatty acids [FA0101]]

6989	-1.754	-1.754	Ph	563.140743	564.148019	0.201658	C26H28O14	52	Apigenin 7-O-[β-D-apiosyl-(1->2)-β-D-glucoside]; 7-O-(β-D-Apiofuranosyl-1,2-β-D-glucosyl)-5,7,4'-trihydroxyflavone; Apiin; 7-[(2-O-D-Apio-β-D-furanosyl-β-D-glucopyranosyl)oxy]-5-hydroxy-2-(4-hydroxy-phenyl)-4H-1-benzopyran-4-one; Apioside	Neoschaftoside	Schaftoside
4292	-1.751	-1.751	Ph	403.139861	404.147137	0.048967	C21H24O8	4	Mallotophenone	Koaburanin [Flavans, Flavanols and Leucoanthocyanidins [PK1202]]	Orientanol A [Pterocarpans [PK1207]]
6404	-1.747	-1.948	Ph	521.202869	522.210145	0.064409	C26H34O11	2	Isobrucein A	Quassimarin	
11	-1.738	-2.234	Cb	161.045532	162.052809	-0.091525	C6H10O5	30	Lichenin; Lichenan	(R)-3,3-Dimethylmalate	3-Ethylmalate
3598	-1.736	-2.111	Cb	375.202440	376.209716	-0.005151	C18H32O8	1	2,3-di-0-hexanoyl-α-glucopyranose [Other acyl sugars [SL05]]		
5732	-1.716	-1.835	Cb	483.078040	484.085316	0.022519	C20H20O14	1	1-O,6-O-Digalloyl-β-D-glucose		
2539	*-1.714*	*-1.762*	Ph	*337.113983*	*338.121259*	*0.392818*	*C12H20N2O9*	*1*	*O18 isotope of 3-Epihydroxymugineic acid*		
2271	-1.706	-1.789	Ph	327.108499	328.115775	-0.129529	C15H20O8	3	Anisatin	Paeonoside	
3967	-1.697	-1.797	Ph	389.124168	390.131444	-0.060382	C20H22O8	5	Piceid; Polydatin; 3,4,5-Trihydroxystilbene-3-β-monoglucoside	Populin	Apigeniflavan 5-O-xyloside [Flavans, Flavanols and Leucoanthocyanidins [PK1202]]
3918	-1.691	-2.229	Cb	387.129645	388.136921	-0.066108	C17H24O10	5	Secologanin; (-)-Secologanin	Geniposide	Verbenalin; Cornin (glycoside)
224	-1.686	-1.919	Cb	209.066629	210.073905	-0.227478	C7H14O7	5	Sedoheptulose; D-altro-Heptulose; D-Sedoheptulose; Volemulose	α-D-Mannoheptulopyranose; Mannoheptulose	β-D-Sedoheptulopyranose; β-D-Sedoheptulose
865	*-1.662*	*-1.427*	Pp	*263.077210*	*264.084487*	*0.530806*	*C9H14N2O7*	*1*	*O18 isotope of L-β-aspartyl-L-glutamic acid [primary amine]*		
4847	-1.659	-1.690	Ph	433.114041	434.121318	0.048061	C21H22O10	32	Naringenin 7-O-β-D-glucoside; Prunin	Phlorizin chalcone; Chalcone 2'-O-glucoside	2',4,4',6'-Tetrahydroxychalcone 4'-O-glucoside; THC 4'-glucoside
3922	-1.659	-1.817	Lp	387.166034	388.173310	-0.057526	C18H28O9	2)	Tuberonic acid glucoside	Tuberonic acid glucoside (see KEGG C08558); (1R,2S)-3-oxo-2-(5'-O-β-D-glucopyranosyloxy-2'Z-pentenyl)-cyclopentaneacetic acid [Fatty acyl glycosides of mono- and disaccharides [FA1301]]	
4814	-1.654	-1.784	Cb	431.155905	432.163181	0.044900	C19H28O11	1	Zizybeoside I		
4594	-1.646	-1.920	Ph	419.134784	420.142061	0.067492	C21H24O9	10	Melampodin A	Glycyphyllin; Phloretin 2'-O-rhamnoside	Isorhapontin
3508	-1.635	-1.997	Lp	371.243903	372.251179	-0.025932	C20H36O6	5	8,8a-Deoxyoleandolide	19(R)-hydroxy-PGF1α; 9S,11R,15S,19R-tetrahydroxy-13E-prostaenoic acid [Prostaglandins [FA0301]]	13,14-dihydro-19(R)-hydroxyPGE1; 9-oxo-11R,15S,19R-trihydroxy-prostanoic acid [Prostaglandins [FA0301]]
6824	-1.633	-1.298	Ph	549.182543	550.189820	0.089857	C23H34O15	1	Genipin 1-β-gentiobioside		
3072	-1.628	-2.222	AmS	356.098668	357.105944	-0.103781	C15H19NO9	1	cyclo-Dopa 5-O-glucoside		

280	-1.628	-1.354	Ph	219.029852	220.037128	-0.204938	C11H8O5	4	(3E)-4-(2-Carboxyphenyl)-2-oxobut-3-enoate; *trans*-2'-Carboxybenzalpyruvate	Purpurogallin	(3Z)-4-(2-Carboxyphenyl)-2-oxobut-3-enoate; *cis*-2'-Carboxybenzalpyruvate
2614	-1.627	-1.623	Cb	341.108884	342.116161	-0.149462	C12H22O11	45	Sucrose; Cane sugar; Saccharose; 1-α-D-Glucopyranosyl-2-β-D-fructofuranoside	Cellobiose; 1-β-D-Glucopyranosyl-4-D-glucopyranose	Maltose; Malt sugar; α-D-Glucopyranosyl-(1->4)-D-glucopyranose
1733	*-1.595*	*-2.225*	Ph	*305.124160*	*306.131437*	*0.457167*	*C12H20N2O7*	*1*	*O18 isotope of 2'-Deoxymugineic acid*		
1252	-1.589	-2.153	NM	277.144506	278.151783	-0.095744	C16H22O4	7	2-Ethylhexyl phthalate	Dibutyl phthalate; Di-n-butyl phthalate; Dibutyl 1,2-benzenedicarboxylate	Diisobutyl phthalate; DIBP
8856	-1.589	-1.463	Lp	831.502796	832.510072	-0.128394	C43H77O13P	12	PI(14:0/20:3(8Z,11Z,14Z)); 1-tetradecanoyl-2-(8Z,11Z,14Z-eicosatrienoyl)-glycero-3-phospho-(1'-myo-inositol) [Diacylglycerophosphoinositols [GP0601]]	PI(14:1(9Z)/20:2(11Z,14Z)); 1-(9Z-tetradecenoyl)-2-(11Z,14Z-eicosadienoyl)-glycero-3-phospho-(1'-myo-inositol) [Diacylglycerophosphoinositols [GP0601]]	PI(17:1(9Z)/17:2(9Z,12Z)); 1-(9Z-heptadecenoyl)-2-(9Z,12Z-heptadecadienoyl)-glycero-3-phospho-(1'-myo-inositol) [Diacylglycerophosphoinositols [GP0601]]
5133	-1.589	-1.698	Ph	449.108952	450.116229	0.038519	C21H22O11	25	Neoastilbin; (2S,3S)-Taxifolin 3-rhamnoside	2',3,4,4',6'-Peptahydroxychalcone 4'-O-glucoside; PHC 4'-O-glucoside	Astilbin
3500	-1.572	-1.811	Ph	371.134722	372.141998	-0.092343	C17H24O9	1	Syringin; Eleutheroside B		
1977	*-1.563*	*-1.563*	NM	*315.144882*	*316.152158*	*0.418470*	*C19H23ClN2*	*1*	*Cl37 isotope of Clomipramine*		
2745	-1.561	-1.756	Lp	345.119057	346.126333	-0.142585	C15H22O9	4	Aucubin	Deutzioside	Aucubin (see KEGG C09771) [C10 isoprenoids (monoterpenes) [PR0102]]
3101	-1.559	-2.041	Cb	357.119069	358.126345	-0.103262	C16H22O9	3	Tarennoside	10-Deoxygeniposidic acid	Sweroside
3145	-1.552	-1.851	Cb	359.098337	360.105614	-0.091802	C15H20O10	1	3-Methoxy-4-hydroxyphenylglycol glucuronide (see KEGG C03033); (2S,3S,4S,5R,6R)-3,4,5-trihydroxy-6-[2-hydroxy-2-(4-hydroxy-3-methoxy-phenyl)ethoxy]oxane-2-carboxylic acid [acetal]		
4329	-1.550	-1.135	Ph	405.082709	406.089985	-0.028041	C19H18O10	7	Lancerin; 4-C-Glucosyl-1,3,7-trihydroxyxanthone	5,7,3',5'-Tetrahydroxy-3,6,8,4'-tetramethoxyflavone [Flavones and Flavonols [PK1211]]	5,7,3',4'-Tetrahydroxy-3,6,8,5'-tetramethoxyflavone [Flavones and Flavonols [PK1211]]
2867	*-1.547*	*-1.591*	NM	*349.114002*	*350.121279*	*0.454376*	*C18H21ClN2O3*	*1*	*Cl37 isotope of Compound VII; 5-(5-(6-Chloro-4-(4,5-dihydro-2-oxazolyl)phenoxy)pentyl)-3-methylisoxazole*		
5363	-1.544	-1.544	Lp	461.363655	462.370932	0.047187	C29H50O4	1	2α-(3-Hydroxypropyl)-1α,25-dihydroxy-19-norvitamin D3; (5Z,7E)-(1R,2R,3R)-3-(hydroxy-propyl)-19-nor-9,10-seco-5,7-cholestadien-1,3,25-triol [Vitamin D3 and derivatives [ST0302]]		
93	-1.534	-1.586	Ph	181.050603	182.057880	-0.160171	C9H10O4	17	3-(4-Hydroxyphenyl)lactate; 4-Hydroxyphenyllactate; p-Hydroxyphenyllactate; 2-	(R)-3-(4-Hydroxyphenyl)lactate	3-(2,3-Dihydroxyphenyl)propanoate; 2,3-Dihydroxyphenylpropanoate

									Hydroxy-3-(4-hydroxyphenyl)propanoate		
2276	-1.509	-1.978	Ph	327.144911	328.152188	-0.047159	C16H24O7	3	Rhododendrin	Tetranor-PGEM; 11R-hydroxy-9,15-dioxo-2,3,4,5-tetranor-prostan-1,20-dioic acid [Prostaglandins [FA0301]]	Prostaglandin M; 7R-hydroxy-5,11-dioxotetranorprostane-1,16-dioic acid [Prostaglandins [FA0301]]
5695	-1.507	-1.486	Ph	481.135145	482.142422	-0.009034	C22H26O12	1	Catalposide	ZZZ	ZZZ
3049	-1.507	-1.808	Ph	355.103413	356.110690	-0.119119	C16H20O9	3	Gentiopicrin; Gentiopicroside	1-O-Feruloyl-β-D-glucose; 1-Feruloyl-D-glucose	Gentiopicrin (see KEGG C09782) [C10 isoprenoids (monoterpenes) [PR0102]]
3857	-1.506	-1.773	Ph	385.113996	386.121273	-0.063172	C17H22O10	2	1-O-Sinapoyl-β-D-glucose; 1-O-Sinapoyl β-D-glucoside	4-O-β-D-Glucosyl-sinapate	
5431	-1.499	-1.784	Ph	465.103882	466.111159	0.069908	C21H22O12	6	Quercetin 7-O-glucoside	Plantagoside	3,4,5,2',4',6'-Hexahydroxychalcone 2'-glucoside [Chalcones and dihydrochalcones [PK1212]]
1255	-1.481	-1.432	Lp	277.217271	278.224548	-0.117541	C18H30O2	47	(6Z,9Z,12Z)-Octadecatrienoic acid; 6,9,12-Octadecatrienoic acid; γ-Linolenic acid; Gamolenic acid	(9Z,12Z,15Z)-Octadecatrienoic acid; α-Linolenic acid; 9,12,15-Octadecatrienoic acid; Linolenate; α-Linolenate	Crepenynate; (9Z)-Octadec-9-en-12-ynoate; (Z)-9-Octadecen-12-ynoic acid; Crepenynic acid
1918	-1.477	-1.477	NM	313.147866	314.155142	0.528679	C19H23ClN2	1	Clomipramine		
5036	-1.454	-1.557	Cb	443.192275	444.199552	0.009678	C21H32O10	1	dihydrophaseic acid 4-O-β-D-glucoside; (2Z,4E)-5-[(1R,3S,5R,8S)-3-(β-D-glucopyranosyloxy)-8-hydroxy-1,5-dimethyl-6-oxabicyclo[3,2,1]oct-8-yl]-3-methylpenta-2,4-dienoic acid [C15 isoprenoids (sesquiterpenes) [PR0103]]		
4853	-1.449	-1.966	NM	433.156296	434.163573	-0.914956	C23H25F3N2OS	1	*cis*-(Z)-Flupenthixol		
3651	-1.399	-1.920	Ph	377.160554	378.167830	-0.061522	C20H26O7	5	Chaparrinone	Cnicin	Eupahyssopin
3109	-1.373	-1.879	Lp	357.264681	358.271958	0.093990	C20H38O5	6	13,14-Dihydro PGF-1a; 7-[(1R,2R,3R,5S)-3,5-dihydroxy-2-[(3S)-3-hydroxyoctyl]cyclopentyl]heptanoic acid [secondary alcohol]	9,13-dihydroxy-10-ethoxy-11-octadecenoic acid [Hydroxy fatty acids [FA0105]]	9,13-dihydroxy-12-ethoxy-10-octadecenoic acid [Hydroxy fatty acids [FA0105]]
2575	-1.360	-1.552	Cb	339.093245	340.100522	-0.117669	C12H20O11	4	Cellobiono-1,5-lactone; Cellobiose-1,5-lactone	3-Ketolactose	3-Ketosucrose; 3-Dehydro-α-D-glucosyl-β-D-fructofuranoside
1346	-1.313	-1.319	Ph	285.040436	286.047712	-0.089823	C15H10O6	34	Luteolin; 3',4',5,7-Tetrahydroxyflavone; 5,7,3',4'-Tetrahydroxyflavone	Kaempferol; 3,4',5,7-Tetrahydroxyflavone; 4H-1-Benzopyran-4-one,3,5,7-trihydroxy-2-(4-hydroxyphenyl)-5,7,4'-Trihydroxyflavonol; C,I, 75640; Indigo yellow; Kaempherol; Kempferol; Nimbecetin; Pelargidenolon; Populnetin; Rhamnolutein; Rhamnolutin; Robigenin; Swartziol; Trifolitin	Aureusidin

2224	-1.312	-1.899	NM	325.129232	326.136508	-0.138273	C16H22O7	1	Hinokitiol glucoside; β-Thujaplicin 2-O-β-D-glucoside		
1138	-1.291	-2.284	Lp	269.175813	270.183090	-0.072009	C15H26O4	1	(10S)-Juvenile hormone III acid diol		
2111	-1.281	-1.568	Ph	321.061550	322.068827	-0.126928	C15H14O8	3	Leucodelphinidin	Elephantorrhizol [Flavans, Flavanols and Leucoanthocyanidins [PK1202]]	Gallocatechin-4β-ol [Flavans, Flavanols and Leucoanthocyanidins [PK1202]]
1217	-1.275	-2.167	Cb	275.077209	276.084485	-0.116446	C11H16O8	1	Ranunculin		
2579	-1.261	-1.687	Cb	339.129643	340.136919	-0.081507	C13H24O10	1	Methyl-2-α-L-fucopyranosyl-β-D-galactoside		
5691	-1.256	-1.697	Ph	481.098785	482.106061	0.042399	C21H22O13	1	3,4,2',3',4',6',α-Heptahydroxychalcone 2'-glucoside [Chalcones and dihydrochalcones [PK1212]]		
2219	-1.245	-1.191	Ph	325.092849	326.100126	-0.128228	C15H18O8	8	4-O-β-D-Glucosyl-4-hydroxycinnamate	*trans*-β-D-Glucosyl-2-hydroxycinnamate; β-D-Glucosyl-2-coumarate	*cis*-β-D-Glucosyl-2-hydroxycinnamate; β-D-Glucosyl-2-coumarinate
6367	-1.245	-1.604	Ph	519.187226	520.194503	0.078545	C26H32O11	2	Brusatol	(-)-Pinoresinol glucoside	ZZZ
1304	-1.212	-1.257	Lp	281.248574	282.255850	-0.106353	C18H34O2	58	(9Z)-Octadecenoic acid; (Z)-Octadec-9-enoic acid; Oleate; Oleic acid	(9E)-Octadecenoic acid; 9-Octadecenoic acid, (E)-; (E)-Oleic acid; D9-*trans*-Octadecenoic acid; 9-*trans*-Octadecenoic acid; *trans*-D9-Octadecenoic acid; *trans*-9-Octadecenoic acid; *trans*-Elaidic acid; *trans*-Oleic acid; Elaidic acid; Acide elaidique; Elaidinsaure	(6Z)-Octadecenoic acid; Petroselinic acid; *cis*-6-Octadecenoic acid
3121	-1.210	-2.451	Pp	358.114330	359.121606	-0.070431	C15H21NO9	1	Epinephrine glucuronide (see KEGG C03033); (2S,3S,4S,6S)-3,4,5,5-tetrahydroxy-3-[(1R)-1-hydroxy-2-methylaminoethyl]-6-(phenoxy)oxane-2-carboxylic acid [acetal]		
1351	-1.201	-1.738	Cb	285.097947	286.105223	-0.104355	C13H18O7	4	Salicin; Salicoside	Gastrodin; 4-(Hydroxymethyl)phenyl-β-D-glucopyranoside	Methylarbutin
1590	-1.196	-1.342	Cb	299.113594	300.120870	-0.109047	C14H20O7	1	Salidroside; p-Hydroxyphenethyl alcohol 1-O-β-D-glucoside; Tyrosol glucoside		
2074	-1.195	-1.764	Cb	319.139804	320.147080	-0.116812	C14H24O8	2	Valproic acid glucuronide (see KEGG C03033); 3,4,5-trihydroxy-6-(2-propylpentanoyloxy)oxane-2-carboxylic acid [secondary alcohol]	Octanoylglucuronide (see KEGG C03033) [secondary alcohol]	
6826	-1.180	-1.180	Ph	549.197747	550.205023	-0.006175	C27H34O12	1	Eucommin A; Medioresinol 4'-O-β-D-glucopyranoside		

60	-1.134	-1.163	Ph	175.024794	176.032070	-0.100946	C6H8O6	14	Ascorbate; Ascorbic acid; L-Ascorbate; L-Ascorbic acid; Vitamin C	D-Glucuronolactone; Glucurone; Glucofuranurono-6,3-lactone; D-Glucurono-3,6-lactone; D-Glucurone	2-Hydroxy-3-oxoadipate
2088	-1.128	-1.784	Pp	320.062277	321.069553	-0.132751	C11H15NO10	1	B-Citryl-L-glutamic acid; 3-[[(2S)-1,5-dihydroxy-1, 5-dioxopentan-2-yl]carbamoyl]-3-hydroxypentanedioic acid [tertiary alcohol]		
1966	-1.101	-1.967	Ph	315.087373	316.094649	-0.123479	C17H16O6	42	Cajanol	Homoferreirin	2,3-Dihydro-6-hydroxy-2-(4-hydroxyphenyl)-5,7-dimethoxy-4H-1-benzopyran-4-one; 4',6-Dihydroxy-5,7-dimethoxyflavanone
1159	-1.096	-1.875	Cb	271.082290	272.089566	-0.135033	C12H16O7	2	Arbutin; Ursin; Uvasol; Hydroquinone-O-β-D-glucopyranoside	α-Arbutin	ZZZ
5385	-1.075	-1.386	Ph	463.088225	464.095501	0.054352	C21H20O12	52	Quercetin 3-O-glucoside; Isoquercitrin; 2-(3,4-Dihidroxyphenyl)-3-(β-D-glucofuranosyloxy)-5,7-dihydroxy-4H-1-benzopyran-4-one; Isotrifoliin	Gossypetin 8-rhamnoside	Hyperin; Quercetin 3-galactoside
4027	*-1.074*	*-2.182*	NM	*391.160971*	*392.168248*	*0.453424*	*C21H27ClN2O3*	*1*	*Cl37 isotope of Compound II(R/S); 5-(7-(6-Chloro-4-(5-hydro-4-methyl-2-oxazolyl)phenoxy)heptyl)-3-methylisoxazole*		
4887	-1.073	-1.375	Ph	435.129686	436.136963	0.036110	C21H24O10	16	Phlorizin; Phlorhizin; Phloridzin	Epiafzelechin 3-O-β-D-allopyranoside [Flavans, Flavanols and Leucoanthocyanidins [PK1202]]	Arthromerin B [Flavans, Flavanols and Leucoanthocyanidins [PK1202]]
5658	-1.052	-1.601	Cb	479.083140	480.090416	0.053553	C21H20O13	18	Gossypin; Gossypetin 8-O-glucoside	4-o-Galloylbergenin	11-o-Galloylbergenin
557	-0.994	-1.259	Cb	253.092852	254.100128	-0.155851	C9H18O8	4	3-β-D-Galactosyl-sn-glycerol; Galactosylglycerol	2-(β-D-Glucosyl)-sn-glycerol	Galactosylglycerol (see KEGG C05401); (2R,3R,4S,5R,6R)-2-[(2R)-2,3-dihydroxypropoxy]-6-(hydroxymethyl)oxane-3,4,5-triol [acetal]
4335	-0.982	-1.113	Ph	405.119098	406.126375	-0.018591	C20H22O9	7	Astringin	Trichocarpin	Anadanthoside [Flavans, Flavanols and Leucoanthocyanidins [PK1202]]
574	-0.956	-0.981	Lp	255.232918	256.240194	-0.141782	C16H32O2	23	Hexadecanoic acid; Hexadecanoate; Hexadecylic acid; Palmitic acid; Palmitate; Cetylic acid	Palmitic acid (see KEGG C00249); Hexadecanoic acid [carboxylic acid]	Trimethyltridecanoic acid; 4,8,12-trimethyltridecanoic acid [carboxylic acid]
2811	-0.946	-1.998	Cb	347.171101	348.178377	-0.116791	C16H28O8	1	Schizonepetoside E		
51	-0.937	-1.303	Ph	173.009145	174.016422	-0.092226	C6H6O6	6	*cis*-Aconitate; *cis*-Aconitic acid	*trans*-Aconitate; *trans*-Aconitic acid	Dehydroascorbate; Dehydroascorbic acid; L-

											Dehydroascorbate; L-Dehydroascorbic acid
3141	-0.931	-1.474	Ph	359.077202	360.084478	-0.109923	C18H16O8	63	Rosmarinate; Rosmarinic acid	3',4',5-Trihydroxy-3,6,7-trimethoxyflavone	6-Methoxyaromadendrin 3-O-acetate
1329	-0.900	-0.949	Lp	283.264219	284.271495	-0.124815	C18H36O2	16	Octadecanoic acid; Stearate; Stearic acid	Stearic acid (see KEGG C01530); Octadecanoic acid [carboxylic acid]	Stearic acid (see KEGG C01530); octadecanoic acid [Straight chain fatty acids [FA0101]]
4597	-0.870	-2.126	Ph	419.155889	420.163166	0.009385	C18H28O11	2	Lamioside	Lamioside (see KEGG C11645) [C10 isoprenoids (monoterpenes) [PR0102]]	
3093	-0.754	-1.035	Ph	357.086062	358.093339	0.982887	C14H19N2O7P	2	N1-(5-Phospho-α-D-ribosyl)-5,6-dimethylbenzimidazole; α-Ribazole 5'-phosphate	N1-(5-Phospho-a-D-ribosyl)-5,6-dimethylbenzimidazole (see KEGG C04778); [(2R,3R,4R,5S)-5-(5,6-dimethylbenzoimidazol-1-yl)-3,4-dihydroxy-oxolan-2-yl]methoxyphosphonic acid [secondary alcohol]	
6072	-0.725	-0.969	Cb	501.146171	502.153448	0.125686	C18H30O16	1	α-L-Rhamnopyranosyl-(1->2)-β-D-galactopyranosyl-(1->2)-β-D-glucuronopyranoside; β-Fabatriose		
2807	-0.702	-1.973	Cb	347.134705	348.141981	-0.146980	C15H24O9	1	Leonuridine		
1186	-0.699	-1.496	Ph	273.076829	274.084105	-0.067655	C15H14O5	26	Phloretin	α-Pyrufuran	β-Pyrufuran
